# Resistance Exercise Therapy After COVID-19 Infection: A Randomized Clinical Trial

**DOI:** 10.1001/jamanetworkopen.2025.34304

**Published:** 2025-11-10

**Authors:** Colin Berry, Gemma McKinley, Hannah K. Bayes, David Anderson, Chim Choy Lang, Adam Gill, Andrew Morrow, Robert Sykes, Diann Taggart, Anna Kamdar, Paul Welsh, Susan Dawkes, Alex McConnachie, Stuart R. Gray

**Affiliations:** 1 British Heart Foundation Glasgow Cardiovascular Research Centre, School of Cardiovascular and Metabolic Health, University of Glasgow, Glasgow, United Kingdom; 2 Department of Cardiology, Queen Elizabeth University Hospital, NHS Greater Glasgow and Clyde Health Board, Glasgow, United Kingdom; 3 Robertson Centre for Biostatistics, School of Health and Wellbeing, University of Glasgow, Glasgow, United Kingdom; 4 Department of Respiratory Medicine, Royal Infirmary, NHS Greater Glasgow and Clyde Health Board, Glasgow, United Kingdom; 5 Department of Respiratory Medicine, Queen Elizabeth University Hospital, NHS Greater Glasgow and Clyde Health Board, Glasgow, United Kingdom; 6 Division of Cardiovascular Research, University of Dundee, Dundee, United Kingdom; 7 Tuanku Muhriz Royal Chair, National University of Malaysia, Kuala Lumpur, Malaysia; 8 Clinical Research Facility, Queen Elizabeth University Hospital, NHS Greater Glasgow and Clyde Health Board, Glasgow, United Kingdom; 9 NHS Project Management Unit, NHS Greater Glasgow and Clyde Health Board, Glasgow, United Kingdom; 10 School of Health and Social Care, Edinburgh Napier University, Edinburgh, United Kingdom; 11 Institute of Sports Science and Innovation, Lithuanian Sports University, Kaunas, Lithuania

## Abstract

**Importance:**

Long COVID presents an unmet therapeutic need.

**Objective:**

To determine the effects of a resistance exercise intervention on exercise capacity, health status, and safety among adults after COVID-19 infection.

**Design, Setting, and Participants:**

A 2-arm, multicenter, randomized clinical trial including 233 adults with a hospital or community diagnosis of COVID-19 infection in the preceding 12 months was undertaken from June 1, 2021, to April 26, 2024. The intervention group comprised 117 individuals, and the control group comprised 116 individuals. A total of 224 individuals at baseline and 193 individuals at 3 months completed Incremental Shuttle Walk Tests.

**Exposures:**

The intervention group received the personalized resistance exercise intervention for 3 months, and the control group received treatment as usual.

**Main Outcomes and Measures:**

The primary outcome was the distance achieved (in meters) in the Incremental Shuttle Walk Test undertaken 3 months after randomization. Secondary outcome measures included health-related quality of life (measured by the European Quality of Life 5-Dimension 5-Level Instrument [EQ-5D-5L]), anxiety and depression (measured by the Patient Health Questionnaire), and grip strength.

**Results:**

A total of 233 adults (median age, 53.6 years [IQR, 43.8-60.8 years]; 146 women [62.7%]; 91 [39.1%] hospitalized with COVID-19 infection) were randomized (117 [50.2%] to the intervention group and 116 [49.8%] to the control group). The median percentage adherence with the exercise intervention was 71.0% (IQR, 47.8%-96.8%), equivalent to performing the exercises 5 days per week. The mean (SD) distance achieved in the Incremental Shuttle Walk Test was 328 (225) m for 224 individuals at baseline and 389 (249) m for 193 individuals at follow-up. The mean (SD) change in Incremental Shuttle Walk Test distance at 3 months compared with baseline was 83 (118) m in the intervention group (n = 94) and 47 (95) m in the control group (n = 98) (adjusted mean difference, 36.5 m [95% CI, 6.6-66.3 m]; *P* = .02). By 3 months, compared with the control group, greater improvements in the intervention group were also observed for the health-related quality of life utility score (EQ-5D-5L) (0.06 [95% CI, 0.01-0.11]; *P* = .02), Patient Health Questionnaire category (0.5 [95% CI, 0.2-0.8]; *P* = .01), and handgrip strength (2.6 kg [95% CI, 0.9-4.2 kg]; *P* = .002).

**Conclusions and Relevance:**

In this randomized clinical trial, a 3-month program of resistance exercise among adults after COVID-19 infection appeared to improve walking distance, health-related quality of life, anxiety, depression, and grip strength. This pragmatic intervention may be a generalizable therapy for individuals with persisting physical symptoms after COVID-19 infection.

**Trial Registration:**

ClinicalTrials.gov Identifier: NCT04900961

## Key Points

**Question:**

What are the effects of a personalized resistance exercise intervention on physical and psychological function after COVID-19 infection?

**Findings:**

In this randomized clinical trial that included 233 adults after COVID-19 infection, the mean change in Incremental Shuttle Walk Test distance at 3 months compared with baseline was 83 m in the intervention group and 47 m in the control group, a statistically significant difference of 36.5 m. Health-related quality of life, anxiety, depression, and grip strength also improved more in the intervention group than in the control group.

**Meaning:**

This study suggests that resistance exercise in a community and posthospitalized post–COVID-19 population improved physical function and psychological well-being.

## Teaser

This randomized clinical trial assesses the effects of a personalized resistance exercise intervention on exercise capacity, health status, and psychological function among adults after COVID-19 infection.

## Introduction

Symptoms, such as breathlessness and fatigue, lasting longer than 3 months after COVID-19 infection represent long COVID.^1^ The effects of long COVID include persisting physical symptoms and psychological symptoms,^2,3,4,5,6,7^ as well as impairments in exercise capacity and health-related quality of life.^5,6,7^ In 2024, the estimated prevalence of long COVID among adults in the UK was 3.3% (2 million people), and in the US, the prevalence estimate was even higher (6.9% [95% CI, 6.5%-7.2%]).^1,2^ Given the paucity of evidence from randomized clinical trials, long COVID presents an unmet therapeutic need.

Individuals living with long COVID may experience impairments in physical function^7^ and skeletal muscle energetics.^8^ Skeletal muscle mass and function are reduced with physical inactivity^9^ and may increase with resistance exercise.^10^ A systematic review of exercise interventions for individuals with long COVID identified improvements in exercise capacity, dyspnea, and health-related quality of life; however, the certainty of evidence was low, treatment effects may have been overestimated, and data on adverse events were lacking.^11^ Accordingly, a randomized clinical trial of resistance exercise to assess cause-and-effect relationships in long COVID was needed.

Building on previous experience of the limitations of exercise for individuals with long COVID,^6^ the objectives of this study were to determine the effects of a resistance exercise intervention on exercise capacity, health status, and safety among adults after COVID-19 infection.^12^

## Methods

### Study Design

A multicenter, parallel-group, 1:1 randomized clinical trial of resistance exercise among adults who had received a diagnosis of COVID-19 in the preceding 12 months and had persisting symptoms was undertaken in Scotland from June 1, 2021, to April 26, 2024. Ethical approval was granted by the National Health Service Research Ethics Committee (trial protocol in Supplement 1). All participants provided written informed consent. The study was publicly registered before the first participant was randomized (NCT04900961). The protocol schedule and amendments are described in eTables 1 and 2 in Supplement 2, respectively, and the study design has been published.^12^ The study has been reported according to the Consolidated Standards of Reporting Trials (CONSORT) reporting guideline for randomized clinical trials (Figure).^13^

**Figure.**
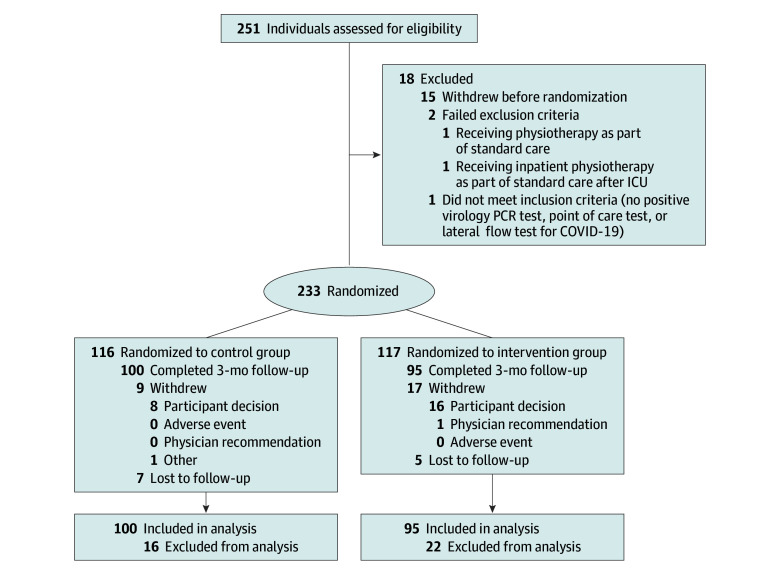
Flow Diagram Recruitment ended when 170 individuals had achieved the primary outcome at 3 months. Other individuals who had given informed consent to participate continued in the study; therefore, 233 individuals were included. ICU indicates intensive care unit; PCR, polymerase chain reaction.

### Setting and Population

The sites were the Queen Elizabeth University Hospital and Royal Infirmary in Glasgow in the west of Scotland and Ninewells Hospital in Dundee in the east of Scotland. Participants were classified according to being not hospitalized due to COVID-19 but as having persisting symptoms for at least 4 weeks leading to medical review (group A); discharged after hospitalization for COVID-19 and with persistent symptoms for at least 4 weeks (group B); or convalescing in the hospital after hospitalization for COVID-19 infection (group C). Participants enrolled in the community were included in groups A and B, while participants enrolled during hospital admission were in group C. Demographic characteristics collected included sex, body mass index, age, self-reported race and ethnicity (Asian [Chinese, Indian, Pakistani, and other Asian], White or not stated, and other [Black African and other ethnic group]), Index of Multiple Deprivation quintile, smoking status, cardiovascular history, COVID-19 history, respiratory history, vital parameters, and COVID-19 diagnosis, severity, and treatment.

### Eligibility Criteria

Patients were eligible for inclusion with a diagnosis of COVID-19 confirmed by (1) virology polymerase chain reaction–positive laboratory diagnosis and/or point-of-care test positive for COVID-19, positive lateral flow test, or positive COVID-19 antibody test; (2) within 12 months of diagnosis; (3) persistent symptoms for at least 4 weeks from symptom onset (groups A and B only); and (4) presentation type (1 of group A, B, or C).

Patients were excluded if they (1) were undergoing inpatient physiotherapy currently part of standard care after an intensive care unit stay, (2) had no expectation of being able to walk within 3 months, (3) were unable to provide informed consent, (4) were unable to comply with the protocol, or (5) had a known pregnancy. On December 14, 2022, given the reduction in incident cases of COVID-19 in the community, the initial eligibility period of 6 months from the diagnosis of COVID-19 (protocol versions 1, 2, and 3) was extended to 12 months (protocol version 4).

### Control Group

The comparator was usual care (treatment as usual) for long COVID, in line with guidelines from the National Institute for Healthcare Excellence guideline,^2^ with no nonroutine contacts from research staff.

### Intervention Group

An exercise program was codesigned by exercise physiologists, physiotherapists, and individuals with lived experience of long COVID. The intervention was developed through discussions with patient groups around the needs of the individual during the exercise intervention, such as staff contact, need for a seated (chair-based) exercise option, and personalization of the exercises, followed by practical exercise sessions involving patients who were hospitalized with COVID-19.

For participants assigned to the intervention group, an instructional pack was provided by research staff supported by an exercise physiologist (S.R.G.). The pack included a guidance document, an exercise log, and links to online videos (eMethods in Supplement 2). The pack was given to participants during an initial face-to-face consultation in which the nurse or therapist helped to select the most suitable category and level of exercise for the participant, demonstrated the exercises, and ensured the participant was comfortable performing the exercise options. Every 2 weeks, each participant was contacted by the research team by telephone or video consultation to provide guidance and support to the participant. If the participant was in the hospital, then the contact was undertaken daily, as needed.

The intervention occurred in the community (groups A and B) or in the hospital and then at home after hospital discharge (group C). Participants were asked to perform the exercises daily. The number of exercise repetitions that led to a validated resistance exercise–specific rating of perceived exertion of 8 to 10 (3-5 of 10 in the first week) was determined.^14^ The use of a rating of perceived exertion to prescribe and titrate resistance exercise is as efficacious as more complex methods but was reported to be better tolerated.^15^ The intervention was tailored according to the preferences of the participant and progress achieved. Additional information is described in eMethods in Supplement 2.

Three exercise categories and guidance were provided according to the status of the participant: (1) confined to bed: lying chest press, lying row, lying plantar flexion, and lying leg press and bridging; (2) able to sit up: seated chest press, seated row, seated lateral raises, seated leg extension, seated plantar flexion, and squats (performed the same as in the ambulatory group); and (3) ambulatory: press ups, standing lateral raises, seated rows, lunges, calf raises, and squats. Participants were asked to perform upper body exercises initially and start the lower body exercises in week 3. The exercise log was intended to be completed by the participant after an episode of exercise activity had been undertaken, whether it was completed and whether any adverse effects occurred.

### Randomization and Blinding

The details of randomization and blinding are described in detail in eMethods in Supplement 2. The statistical analysis plan (Supplement 1) was finalized, and all statistical programs were written and validated prior to database lock and unblinding.

### Outcomes

#### Primary Outcome

The primary outcome was the distance achieved (in meters) during the Incremental Shuttle Walk Test, an externally paced incremental walking test developed as a measure of exercise capacity.^16^ Participants were required to walk around 2 marker cones, 9 m apart, placed 0.5 m from each end point (10-m course) with an initial speed of 0.5 m/seconds, increasing 0.17 m/seconds every minute. Audio cues (beeps) signal the time at which the participant should turn at the marker. The test has 12 levels (walking speeds), and the maximum duration of the test is therefore 12 minutes. No encouragement was given during the test; the only verbal cues provided referred to an impending increase in walking speed.^17^ The Incremental Shuttle Walk Test performance was defined as the distance achieved,^17^ and oxygen saturation and heart rate were measured.^18^

The duration of the Incremental Shuttle Walk Test correlates with peak oxygen consumption (in milliliters per minute per kilogram) and has population reference values for the distance walked (in meters).^19^ The test has been evaluated and validated in several populations, including healthy women,^20^ young men,^21^ individuals with obesity,^22^ and patients with chronic respiratory disease.^14,16,17,18^ The Incremental Shuttle Walk Test is recognized for being safe and responsive to the effects of rehabilitation in populations with chronic respiratory disease,^18^ and stakeholder organizations support the use of this test as an efficacy measure in clinical trials.^19^

#### Secondary Outcomes

The following secondary outcomes were assessed. Respiratory function was assessed using spirometry. Physical function was measured using handgrip strength and the Short Physical Performance Battery.^23^ Patient-reported outcome measures included health-related quality of life (assessed with the European Quality of Life 5-Dimension 5-Level Instrument [EQ-5D-5L]),^24^ the 4-item Patient Health Questionnaire,^25^ illness perception (assessed with the Brief Illness Perception Questionnaire),^26^ the Duke Activity Status Index,^27^ and the short form of the International Physical Activity Questionnaire.^28^ Fatigue was measured using the Medical Research Council dyspnea score.^29^ Frailty was assessed using 5 criteria from the Fried frailty phenotype (weight loss, exhaustion, grip strength, low physical activity, and slow walking pace)^30^ and the Clinical Frailty Scale.^31^ Clinical outcomes and adverse events were episodes of health care and hospitalization for any reason.

#### Additional Prespecified Outcomes

Other outcomes were prespecified. (1) Vital parameters of cardiorespiratory function (eg, oxygen saturation, heart rate, and respiratory rate at baseline and during follow-up) were assessed. (2) Adherence with exercise was assessed in the intervention group. (3) Postexercise adverse events and malaise (adverse events during and after exercise) were assessed in all participants. Following a protocol amendment, the DePaul Symptom Questionnaire (Short Form)^32^ was assessed in a subgroup. (4) Accelerometry (Glasgow site) was used to collect data on acceleration and was calibrated to local gravity,^33,34,35,36,37^ and physical activity levels were quantified using the GGIR package in R, versions 4.3.0 and 4.4.1 (R Project for Statistical Computing), with methods previously described^35^ (eMethods in Supplement 2). (5) COVID-19 serology was determined using the SARS-CoV-2 IgG II Quant assay.^38^

### Bias Minimization and Sample Size Calculation

Bias minimization procedures are described in the eMethods in Supplement 2. A predetermined sample size calculation^16^ was devised by a biostatistician coauthor (A.M.) (eMethods in Supplement 2).

### Statistical Analysis

The primary and secondary outcomes were analyzed using linear regression (continuous outcomes) or proportional odds logistic regression (ordinal outcomes). All models were adjusted for the baseline value of the outcome variable (eMethods in Supplement 2). All *P* values were from 2-sided tests and results were deemed statistically significant at *P* < .05.

## Results

Between June 1, 2021, and April 26, 2024, 250 individuals were screened, and 233 individuals (median age, 53.6 years [IQR, 43.8-60.8 years]; 146 women [62.7%] and 87 men [37.3%]; 14 Asian individuals [6.0%], 217 White individuals [93.1%], and 2 individuals of other race or ethnicity [0.9%]; 58 individuals [25.0%] resided in areas with the most socioeconomic deprivation; and 91 individuals [39.1%] were hospitalized) were randomized (Table 1). All participants had circulating immunoglobulin G (IgG) antibodies to SARS-CoV-2, consistent with prior COVID-19 and/or vaccination. A total of 117 individuals (50.2%) were assigned to the intervention group and 116 individuals (49.8%) were assigned to the control group. Almost two-thirds of the population (145 [62.2%]) experienced symptoms 90 days or more after the diagnosis of COVID-19. The flow diagram is illustrated in the Figure. The participants’ characteristics are described in Table 1 and eTables 3 and 4 in Supplement 2.

**Table 1.** Baseline Characteristics of the Study Population

Characteristic	All (N = 233)	Standard care (n = 116)	Intervention (n = 117)
Demographic characteristics			
Male, No. (%)	87 (37.3)	44 (37.9)	43 (36.8)
Female, No. (%)	146 (62.7)	72 (62.1)	74 (63.2)
Age, median (IQR), y	53.6 (43.8-60.8)	52.1 (43.6-60.1)	54.6 (43.9-60.8)
BMI, median (IQR)	29.8 (25.9-33.7)	30.3 (26.0-34.0)	29.3 (25.8-33.2)
Clinical presentation group, No. (%)^a^			
Group A	145 (62.2)	72 (62.1)	73 (62.4)
Group B	66 (28.3)	33 (28.4)	33 (28.2)
Group C	22 (9.4)	11 (9.5)	11 (9.4)
Hospitalized, No. (%)	91 (39.1)	45 (38.8)	46 (39.3)
Race and ethnicity, No. (%)			
Asian^b^	14 (6.0)	6 (5.2)	8 (6.8)
White or not stated	217 (93.1)	110 (94.8)	107 (91.5)
Other^c^	2 (0.9)	0	2 (1.7)
Index of Multiple Deprivation, quintile, No. (%)			
No. missing	1	0	1
First (most deprived)	58 (25.0)	36 (31.0)	22 (19.0)
Second	39 (16.8)	20 (17.2)	19 (16.4)
Third	30 (12.9)	13 (11.2)	17 (14.7)
Fourth	37 (15.9)	13 (11.2)	24 (20.7)
Fifth (least deprived)	68 (29.3)	34 (29.3)	34 (29.3)
Smoking status, No. (%)			
Never	147 (63.1)	73 (62.9)	74 (63.2)
Former	74 (31.8)	37 (31.9)	37 (31.6)
Current (<10 cigarettes/d)	4 (1.7)	2 (1.7)	2 (1.7)
Current (10-19 cigarettes/d)	2 (0.9)	0	2 (1.7)
Current (≥20 cigarettes/d)	6 (2.6)	4 (3.4)	2 (1.7)
Cardiovascular history, No. (%)			
Hypertension	59 (25.3)	34 (29.3)	25 (21.4)
Angina	13 (5.6)	6 (5.2)	7 (6.0)
Myocardial infarction	8 (3.4)	5 (4.3)	3 (2.6)
Stroke	9 (3.9)	4 (3.4)	5 (4.3)
Atrial fibrillation	9 (3.9)	5 (4.3)	4 (3.4)
COVID-19 previous history, No. (%)			
COVID-19 pneumonia	70 (30.0)	35 (30.2)	35 (29.9)
History of COVID-19 reinfection	44 (18.9)	21 (18.1)	23 (19.7)
History of vaccination for COVID-19	221 (95.3)	113 (97.4)	108 (93.1)
Respiratory history, No. (%)			
Asthma	47 (20.2)	25 (21.6)	22 (18.8)
Chronic obstructive pulmonary disease	12 (5.2)	8 (6.9)	4 (3.4)
Sleep apnea syndrome	6 (2.6)	4 (3.4)	2 (1.7)
Vital parameters			
Systolic blood pressure, median (IQR), mm Hg	129.0 (120.0-140.5)	129.2 (120.0-138.2)	127.8 (118.5-143.1)
Diastolic blood pressure, median (IQR), mm Hg	80.5 (72.5-88.6)	79.8 (72.9-88.6)	81.0 (72.5-88.6)
Heart rate, median (IQR), beats/min	74.0 (66.0-82.2)	76.0 (66.0-84.2)	72.5 (65.8-81.0)
Respiratory rate, median (IQR), breaths/min	16.0 (14.0-17.0)	16.0 (14.0-16.0)	16.0 (14.0-17.2)
Oxygen saturation, median (IQR), %	98.0 (97.0-98.0)	98.0 (97.0-98.0)	98.0 (97.0-98.0)
COVID-19 diagnosis, severity, and treatment			
Days since symptom onset, median (IQR)	144 (72-480)	124 (72-438)	152 (73-480)
Individuals with symptoms ≥90 d, No. (%)	145 (62.2)	72 (62.1)	73 (62.4)
Positive diagnostic test for COVID-19, No. (%)			
PCR test	186 (79.8)	90 (77.6)	96 (82.1)
Serology test	11 (4.7)	8 (6.9)	3 (2.6)
Lateral flow test	96 (41.2)	54 (46.6)	42 (35.9)
Radiology diagnosis, No. (%)			
Chest radiograph	67 (28.8)	31 (26.7)	36 (30.8)
Chest CT scan	41 (17.6)	17 (14.7)	24 (20.5)
COVID-19 diagnosis by virology or radiology test	231 (99.1)	115 (99.1)	115 (99.1)
WHO Clinical Severity scale for acute illness, No. (%)			
No limitation of activities	33 (14.2)	17 (14.7)	16 (13.8)
Limitation of activities	128 (55.2)	66 (56.9)	62 (53.4)
Hospitalized, no oxygen therapy	26 (11.2)	11 (9.5)	15 (12.9)
Noninvasive ventilation of high-flow oxygen	10 (4.3)	6 (5.2)	4 (3.4)
Intubation and mechanical ventilation	1 (0.4)	1 (0.9)	0
Ventilation and additional organ support	0	0	0
Hospitalized, with oxygen therapy	34 (14.7)	15 (12.9)	19 (16.4)
Blood test results			
C-reactive protein, median (IQR), mg/dL	0.2 (0.1-0.6)	0.3 (0.1-0.7)	0.2 (0.1-0.5)
C-reactive protein >0.5 mg/dL, No. (%)	58 (27.6)	34 (31.8)	34 (31.8)
Hemoglobin, median (IQR), g/dL	13.9 (13.0-14.8)	13.9 (12.8-15.1)	13.9 (13.0-14.7)
Hemoglobin <12.0 g/dL (females), No./total No. (%)	11/142 (7.7)	8/71 (11.3)	3/71 (4.2)
Hemoglobin <13.0 g/dL (males), No./total No. (%)	8/85 (9.4)	6/44 (13.6)	2/41 (4.9)
COVID-19 serology at 3 mo, median (IQR), AU/mL	9896 (4516-19 878)	10 494 (5694-21 127)	10 494 (5694-21 127)

Abbreviations: BMI, body mass index (calculated as weight in kilograms divided by height in meters squared); CT, computed tomography; PCR, polymerase chain reaction; WHO, World Health Organization.

SI conversion factors: To convert C-reactive protein to milligrams per liter, multiply by 10.0; and hemoglobin to grams per liter, multiply by 10.0.

^a^ Clinical presentation groups: group A: positive diagnosis with persisting symptoms for at least 4 weeks from onset of symptoms leading to medical review, but not admission; group B: positive diagnosis with postdischarge, persistent symptoms for at least 4 weeks after symptom onset; group C: positive diagnosis, in convalescent phase in hospital.

^b^ Asian included Indian, Pakistani, Chinese, and other Asian.

^c^ Other included Black African and other ethnic group.

### Intervention, Adherence, and Primary Outcome

The median percentage of participants who adhered with the exercise intervention was 71.0% (IQR, 47.8%-96.8%), equivalent to performing the exercises 5 days per week. The mean (SD) distance achieved in the Incremental Shuttle Walk Test at baseline was 328 (225) m for 224 individuals and389 (249) m for 193 individuals at follow-up (eTables 5-8 in Supplement 2).

The overall withdrawal rate was 11.2% (26 of 233). The reasons for stopping the Incremental Shuttle Walk Test and the reasons for the withdrawals are described in eTables 5 to 9 and eFigures 1 and 2 in Supplement 2. Prior to the follow-up assessment, 1 participant in the control group experienced a lower limb injury unrelated to the protocol and did not complete the primary outcome evaluation.

The primary outcome analysis is described in Table 2 and in eTables 5 to 9 in Supplement 2. The mean (SD) change in Incremental Shuttle Walk Test distance at 3 months compared with baseline was 83 (118) m in the intervention group (n = 94) and 47 (95) m in the control group (n = 98) (the adjusted mean difference, 36.5 m [95% CI, 6.6-66.3 m]; *P* = .02). The distribution in the distances achieved during the Incremental Shuttle Walk Test at baseline and 3 months after randomization and the change at 3 months from baseline are shown in eFigure 1 in Supplement 2. The complier mean causal effects analyses for the intervention effect estimates on the primary outcome of distance (in meters) are shown in eTable 8 and eFigure 3 in Supplement 2.

**Table 2.** Primary Outcome Analysis

Outcome	All (N = 233)	Standard care (n = 116)	Intervention (n = 117)
**Baseline ISWT distance, m**			
No. (No. missing)	224 (9)	112 (4)	112 (5)
Median (IQR) [range]	270 (180 to 430) [10 to 1230]	270 (180 to 450) [30 to 1230]	270 (180 to 422) [10 to 1030]
**3-mo Follow-up ISWT distance, m**			
No. (No. missing)	193 (2)	99 (1)	94 (1)
Median (IQR) [range]	340 (200 to 520) [40 to 1290]	340 (195 to 465) [60 to 1290]	350 (200 to 528) [40 to 1080]
**Change in ISWT distance, m**			
No. (No. missing)	192 (3)	98 (2)	94 (1)
Median (IQR) [range]	40 (0 to 110) [−170 to 460]	30 (0 to 90) [−170 to 440]	65 (10 to 140) [−170 to 460]
**Linear regression intervention effect estimate**			
Difference (95% CI), m	NA	NA	36.5 (6.6 to 66.3)
*P* value	NA	NA	.02

Abbreviations: ISWT, Incremental Shuttle Walk Test; NA, not applicable.

There was no statistically significant interactions on the primary outcome analysis by sex, race and ethnicity, clinical presentation group, socioeconomic quintile, age, COVID-19 serology, or COVID-19 symptom onset of 90 days or more (Table 3).

**Table 3.** Interactions Between Treatment Effect on the Primary Outcome Analysis and Predefined Subgroups

Characteristic	No. (missing)	Change in ISWT distance at 3 mo, m	Intervention effect estimate (95% CI)	*P* value	*P* value for interaction
Sex					
Male	73 (14)	50.0 (0.0 to 120.0)	8.2 (−40.6 to 57.1)	.74	.15
Female	119 (27)	40.0 (0.0 to 100.0)	53.6 (15.6 to 91.5)	.009	
Race and ethnicity					
White or not stated	179 (38)	40.0 (0.0 to 110.0)	38.3 (7.2 to 69.4)	.02	.56
Other racial or ethnic group	13 (3)	40.0 (10.0 to 100.0)	17.3 (−103.6 to 138.1)	.78	
Clinical presentation group^a^					
Group A	124 (21)	45 (0 to 122.5)	30.9 (−6.4 to 68.3)	.11	.61
Group B	119 (27)	30.0 (−5.0 to 90.0)	36.1 (−20.2 to 92.5)	.21	
Group C	13 (9)	80.0 (30.0 to 130.0)	90.9 (−24.6 to 206.4)	.12	
Index of Multiple Deprivation quintile					
First (most deprived)	51 (7)	30.0 (−10.0 to 80.0)	56.8 (−2.7 to 116.2)	.06	.75
Second	29 (10)	80.0 (30.0 to 130.0)	63.0 (−16.1 to 142.2)	.12	
Third	25 (5)	30.0 (0.0 to 80.0)	−9.6 (−95.2 to 76.0)	.83	
Fourth	29 (8)	40.0 (−10.0 to 120.0)	31.0 (−50.4 to 112.3)	.46	
Fifth (least deprived)	57 (11)	80.0 (0.0 to 110.0)	42.4 (−13.1 to 97.8)	.13	
Age, y					
Tertile 1: (20.9 to ≤49.1)	65 (13)	70.0 (0.0 to 140.0)	40.2 (−11.7 to 92.2)	.13	.91
Tertile 2: (>49.1 to ≤58.2)	63 (14)	30.0 (−5.0 to 100.0)	26.3 (−26.9 to 79.4)	.33	
Tertile 3: (>58.2 to 83.8)	64 (14)	40.0 (7.5 to 90.0)	40.6 (−11.9 to 93.2)	.13	
COVID-19 serology, AU/mL					
Tertile 1: (69 to ≤5927)	61 (0)	40.0 (0.0 to 120.0)	29.4 (−25.8 to 84.6)	.30	.80
Tertile 2: (>5927 to ≤14 151)	61 (0)	10.0 (−40.0 to 90.0)	25.5 (−29.9 to 80.9)	.37	
Tertile 3: (>14 151 to 354 806)	59 (2)	10.0 (−40.0 to 90.0)	52.8 (−1.7 to 107.2)	.06	
COVID-19 symptom duration					
Onset ≥90 d	117 (28)	61.5 (99.0)	31.08 (−7.60 to 69.76)	.12	.67
Onset <90 d	75 (13)	69.3 (121.7)	44.84 (−3.72 to 93.40)	.07	

Abbreviation: ISWT, Incremental Shuttle Walk Test.

^a^ Clinical presentation groups: group A: positive diagnosis with persisting symptoms for at least 4 weeks from onset of symptoms leading to medical review, but not admission; group B: positive diagnosis with postdischarge, persistent symptoms for at least 4 weeks after symptom onset; group C: positive diagnosis, in convalescent phase in hospital.

### Secondary Outcomes

By 2 months after randomization, compared with the control group, greater improvements in the intervention group were observed for the health-related quality of life utility score (EQ-5D-5L) (0.06 [95% CI, 0.01-0.11]; *P* = .02), the 4-item Patient Health Questionnaire category (0.5 [95% CI, 0.2-0.8]; *P* = .01), and handgrip strength (2.6 kg [95% CI, 0.9-4.2 kg]; *P* = .002) (Table 4; eFigures 4-6 in Supplement 2). No effects on any other outcomes were noted (eTables 9-28 in Supplement 2).

**Table 4.** Secondary Outcomes: Change at 3 Months From Baseline and Effect of the Intervention^a^

Outcome	All (N = 233)	Standard care (n = 116)	Intervention (n = 117)	Effect estimate (95% CI)	*P* value
**Spirometry**					
Change in peak expiratory flow rate, L/mo					
No. (No. missing)	187 (8)	96 (4)	91 (4)	NA	NA
Median (IQR)	−5.00 (−46.00 to 35.50)	3.50 (−41.25 to 35.25)	−8.00 (−50.00 to 34.00)	−0.80 (−23.04 to 21.45)	.94
Change in forced vital capacity, L					
No. (No. missing)	187 (8)	96 (4)	91 (4)	NA	NA
Median (IQR)	0.00 (−0.10 to 0.20)	0.00 (−0.10 to 0.20)	0.00 (−0.10 to 0.20)	0.02 (−0.08 to 0.13)	.70
Change in forced expiratory volume in 1 second, L					
No. (No. missing)	187 (8)	96 (4)	91 (4)	NA	NA
Median (IQR)	0.00 (−0.10 to 0.10)	0.00 (−0.10 to 0.10)	0.00 (−0.10 to 0.15)	0.00 (−0.13 to 0.14)	.95
Change in forced expiratory volume/forced expiratory volume ratio					
No. (No. missing)	187 (8)	96 (4)	91 (4)	NA	NA
Median (IQR)	0.00 (−0.03 to 0.02)	0.00 (−0.02 to 0.02)	0.00 (−0.03 to 0.02)	−0.01 (−0.04 to 0.02)	.54
**Physical function**					
Change in handgrip strength, kg					
No. (No. missing)	193 (2)	99 (1)	94 (1)	NA	NA
Median (IQR)	1.7 (−1.9 to 4.6)	0.9 (−3.0 to 3.5)	2.5 (−0.8 to 5.8)	2.6 (0.9 to 4.2)	.002
Change in SPPB score category					
No. (No. missing)	174 (21)	86 (14)	88 (7)	NA	NA
No. (%) improvement	38 (21.8)	13 (15.1)	25 (28.4)	NA	NA
No. (%) no change	124 (71.3)	69 (80.2)	55 (62.5)	NA	NA
No. (%) deterioration	12 (6.9)	4 (4.7)	8 (9.1)	1.7 (0.7 to 3.8)	.23
Change in Duke Activity Status Index					
No. (No. missing)	190 (5)	97 (3)	93 (2)	NA	NA
Median (IQR)	3.35 (0.00 to 9.88)	3.50 (−2.70 to 9.00)	2.75 (0.00 to 10.70)	0.92 (−2.05 to 3.89)	.54
Change in Duke Activity Status Index predicted maximum VO_2_, O_2_ mL/min/kg of body weight					
No. (No. missing)	190 (5)	97 (3)	93 (2)	NA	NA
Median (IQR)	1.44 (0.00 to 4.25)	1.50 (−1.16 to 3.87)	1.18 (0.00 to 4.60)	0.40 (−0.88 to 1.67)	.54
Change in International Physical Activity Questionnaire category					
No. (No. missing)	195 (0)	100 (0)	95 (0)	NA	NA
No. (%) improvement	49 (25.1)	22 (22.0)	27 (28.4)	NA	NA
No. (%) no change	110 (56.4)	61 (61.0)	49 (51.6)	NA	NA
No. (%) deterioration	36 (18.5)	17 (17.0)	19 (20.0)	1.1 (0.7 to 2.0)	.65
**Patient-reported outcome measures**					
Change in EQ-5D-5L Health Utility score					
No. (No. missing)	195 (0)	100 (0)	95 (0)	NA	NA
Median (IQR)	0.00 (−0.05 to 0.14)	0.00 (−0.07 to 0.07)	0.01 (−0.02 to 0.21)	0.06 (0.01 to 0.11)	.02
Change in EQ-5D-5L Visual Analogue scale					
No. (No. missing)	195 (0)	100 (0)	95 (0)	NA	NA
Median (IQR)	5.0 (−2.5 to 14.5)	0.0 (−3.5 to 10.0)	5.0 (0.0 to 15.0)	3.6 (−0.7 to 8.0)	.10
Change in PHQ-4 score category					
No. (No. missing)	193 (2)	99 (1)	94 (1)	NA	NA
No. (%) improvement	52 (26.9)	18 (18.2)	34 (36.2)	NA	NA
No. (%) no change	107 (55.4)	61 (61.6)	46 (48.9)	NA	NA
No. (%) deterioration	34 (17.6)	20 (20.2)	14 (14.9)	0.5 (0.2 to 0.8)	.01
Change in Brief Illness Perception score					
No. (No. missing)	194 (1)	100 (0)	94 (1)	NA	NA
Median (IQR)	−4.00 (−12.00 to 3.00)	−2.50 (−9.25 to 3.25)	−6.00 (−13.75 to 1.00)	−3.52 (−7.25 to 0.22)	.07
**Fatigue and breathlessness**					
Change in Fatigue Severity score					
No. (No. missing)	194 (1)	100 (0)	94 (1)	NA	NA
Median (IQR)	−2 (−10 to 2)	0 (−7 to 3)	−4 (−13 to 1)	−2.60 (−6.38 to 1.18)	.18
Fatigue Visual Analogue scale					
No. (No. missing)	192 (3)	98 (2)	94 (1)	NA	NA
No. (%) improvement	91 (47.4)	48 (49.0)	43 (45.7)	NA	NA
No. (%) no change	30 (15.6)	16 (16.3)	14 (14.9)	NA	NA
No. (%) deterioration	71 (37.0)	34 (34.7)	37 (39.4)	0.9 (0.6 to 1.6)	.83
Change in MRC Dyspnea score					
No. (No. missing)	195 (0)	100 (0)	95 (0)	NA	NA
No. (%) improvement	59 (30.3)	28 (28.0)	31 (32.6)	NA	NA
No. (%) no change	107 (54.9)	57 (57.0)	50 (52.6)	NA	NA
No. (%) deterioration	29 (14.9)	15 (15.0)	14 (14.7)	0.9 (0.5 to 1.5)	.64
Change in Fried frailty					
No. (No. missing)	195 (0)	100 (0)	95 (0)	NA	NA
No. (%) improvement	56 (28.7)	28 (28.0)	28 (29.5)	NA	NA
No. (%) no change	123 (63.1)	65 (65.0)	58 (61.1)	NA	NA
No. (%) deterioration	16 (8.2)	7 (7.0)	9 (9.5)	0.8 (0.4 to 1.5)	.41
Change in current clinical frailty					
No. (No. missing)	191 (4)	98 (2)	93 (2)	NA	NA
No. (%) improvement	77 (40.3)	37 (37.8)	40 (43.0)	NA	NA
No. (%) no change	77 (40.3)	39 (39.8)	38 (40.9)	NA	NA
No. (%) deterioration	37 (19.4)	22 (22.4)	15 (16.1)	0.6 (0.4 to 1.0)	.07

Abbreviations: EQ-5D-5L, European Quality of Life 5-Dimension 5-Level Instrument; MRC, Medical Research Council; NA, not applicable; PHQ-4, 4-item Patient Health Questionnaire; SPPB, Short Physical Performance Battery; VO_2_, volume of oxygen consumption.

^a^ For continuous outcomes, a linear regression intervention effect estimate is provided for the follow-up value. This estimate is adjusted for the baseline value and randomized group, as well as clinical presentation, history of COVID-19 pneumonia, age, sex, and site. A similar approach is taken for ordinal variables, and an ordinal regression intervention effect estimate is provided.

### Safety

#### Adverse Events

No deaths occurred. Ten hospitalizations occurred (9 hospitalizations for 5 individuals in the control group and 1 hospitalization in the intervention group, deemed unrelated to the intervention) (Fisher exact test, *P* = .12; eTable 30 in Supplement 2)

#### Postexercise Malaise

Of 99 individuals who completed the DePaul Symptom Questionnaire, 40 of 48 (83.3%) in the intervention group and 42 of 51 (82.4%) in the control group experienced postexertional malaise at 3-month follow-up (eTables 25-28 in Supplement 2).

#### Rehabilitation in Standard Care

Visits involving physiotherapy and rehabilitation as per standard care in the community are described in eTable 30 in Supplement 2.

## Discussion

In this randomized clinical trial, a program of personalized resistance exercise for community-dwelling and posthospitalized individuals after COVID-19 infection improved exercise capacity. There was no evidence of intervention effect heterogeneity across prespecified groups, including by age, sex, race and ethnicity, socioeconomic quintile, clinical presentation group, COVID-19 serology titer, and COVID-19 symptom onset of 90 days or more (including for individuals with long COVID). In addition, health-related quality of life, anxiety, depression, and grip strength were also improved. Adherence with resistance exercise was reasonably high, and postexercise malaise and adverse events were not increased with the exercise intervention. Participants in both groups received rehabilitation as per standard care.

The median age of the participants was 54 years, two-thirds were female, and one-fourth resided in areas with the most socioeconomic deprivation. Two-thirds of individuals reported persisting symptoms 90 days or more after the diagnosis of COVID-19, and 2 in 5 individuals had received hospital care for COVID-19. Laboratory testing of immune serology in a blood sample provided by each participant at 3 months confirmed evidence of circulating IgG antibodies to SARS-CoV-2 in all of the participants, consistent with prior COVID-19 infection and/or vaccination.

Overall, the withdrawal rate was relatively low (11.2% overall), adherence with the intervention was relatively high (median, 71.0%; equivalent to performing the exercises 5 days/week), and no adverse events or serious adverse events occurred in relation to the intervention. The exercise program was personalized according to the needs and preferences of the individuals. The resistance exercise intervention was unsupervised; hence, this participant-led intervention had minimal or no dependency on health care staff. The results indicate that the intervention may be generalizable in primary and secondary care.

Improvements were observed in some of the prespecified secondary outcomes. Health-related quality of life, psychological well-being, and grip strength improved with the intervention, with the magnitude of these improvements reflecting moderate potential benefits in health and well-being and physical strength. Most participants performed well in the Short Physical Performance Battery at baseline, which may explain the lack of any improvement during follow-up. Illness perception also improved, but the change was not statistically significant. Measures of physical activity, including accelerometry and patient-reported fatigue or perception of frailty, did not improve. The reasons may reflect the participants’ interactions with their environment, a lack of statistical power, and/or a true lack of effect on these outcomes.

The Incremental Shuttle Walk Test is an established measure of functional capacity for rehabilitation among people with respiratory disease. Muscle strength is a determinant of Incremental Shuttle Walk Test performance,^39^ and a decrease in muscle strength after COVID-19 is associated with decreased Incremental Shuttle Walk Test performance.^40^ Muscle strength may be impaired after COVID-19^41^; hence, muscle strength after COVID-19 represented a target for the resistance exercise intervention in this trial, with the overall aim of improving functional capacity. For these reasons, our trial tested resistance exercise as a method to improve Incremental Shuttle Walk Test performance. Prior mechanistic studies indicate that resistance exercise interventions may enhance muscle strength and aerobic fitness,^42,43^ which are determinants of functional capacity. The mechanisms may involve improvements in type II muscle fiber recruitment,^44^ flow-mediated vasodilatation,^45^ and ventricular stroke volume.^46,47,48^ There are other clinical trials of exercise interventions in long COVID,^11,49,50^ and the design and results of our trial in relation to these prior studies are discussed in the eDiscussion in Supplement 2.

### Limitations

This study has some limitations. The Incremental Shuttle Walk Test and interviews were conducted by study personnel who were aware of the treatment allocation. Although the intervention was not masked, the statistical analysis was undertaken blinded to the randomized group. There was no statistically significant evidence of interactions on the primary analysis; however, this analysis was not powered for tests of interactions. Exercise was unsupervised, and adherence was self-reported. Participants in the intervention group received more contact with site staff compared with participants in the control group. The reasons for being lost to follow-up were not available.

These limitations are relevant but should not undermine the overall validity of this trial, which involved a pragmatic intervention and a multicenter design with broad inclusion criteria. Most of the participants were female. Because there was no evidence of intervention effect heterogeneity across prespecified groups, the results may be considered generalizable.

## Conclusions

In this randomized clinical trial, a program of resistance exercise for 3 months among adults after COVID-19 infection appeared to improve walking distance, health-related quality of life, anxiety, depression, and grip strength. The intervention did not increase adverse events or postexertional malaise. This pragmatic intervention may be a generalizable therapy for individuals with persisting physical symptoms after COVID-19 infection.

## Notes

Supplement 1.
Trial Protocol and Statistical Analysis Plan

Supplement 2. **eAppendix 1.** Abbreviations **eAppendix 2.** Protocol Amendments
eMethods.

eDiscussion.
**eTable 1.** Schedule of Enrollment, Interventions and Assessments **eTable 2.** Number of Participants Randomized Under Each Protocol Version at Each Site **eTable 3.** Baseline Characteristics: COVID-19 Treatment **eTable 4.** COVID-19 Reinfection and Vaccination **eTable 5.** Primary Outcome: Incremental Shuttle Walk Test Distance (m) at Baseline **eTable 6.** Primary Outcome: Incremental Shuttle Walk Test Distance (m) at 3 Months **eTable 7.** Primary Outcome Analysis: Incremental Shuttle Walk Test Distance (m) With Imputed Data **eTable 8.** Primary Outcome - Causal Effect Analysis at Various Compliance Levels **eTable 9.** Primary Outcome: Incremental Shuttle Walk Test in subjects With Good Exercise Adherence (>70%, Intervention Group Only) by Clinical Presentation Group **eTable 10.** Secondary Outcome Analysis: Spirometry - Peak Expiratory Flow Rate (L/m) **eTable 11.** Secondary Outcome Analysis: Spirometry - Forced Vital Capacity (L) **eTable 12.** Secondary Outcome Analysis: Spirometry - Forced Expiratory Volume in One Second (L) **eTable 13.** Secondary Outcome Analysis: Spirometry - FEV1/FVC Ratio **eTable 14.** Secondary Outcome Analysis: Handgrip Strength (kg) **eTable 15.** Secondary Outcome Analysis: Short Physical Performance Battery Score Category **eTable 16.** Secondary Outcome Analysis: EQ-5D-5L Utility Score (UK Crosswalk Value Set) **eTable 17.** Secondary Outcome Analysis: EQ-5D-5L Visual Analogue Scale **eTable 18.** Secondary Outcome Analysis: Patient Health Questionnaire Category **eTable 19.** Secondary Outcome Analysis: Brief Illness Perception Questionnaire Score **eTable 20.** Secondary Outcome Analysis: Duke Activity Status Index Score **eTable 21.** Secondary Outcome Analysis: Duke Activity Status Index Predicted VO2max **eTable 22.** Secondary Outcome Analysis: International Physical Activity Questionnaire (Short Form) **eTable 23.** Secondary Outcome Analysis: Fatigue Severity Score **eTable 24.** Secondary Outcome Analysis: MRC Dyspnea Score **eTable 25.** Secondary Outcome Analysis: Fried Frailty (Sum of Phenotypes) **eTable 26.** Post-Exertional Malaise at 3-Months, Part 1 (DePaul Symptom Questionnaire, Short Form) **eTable 27.** Post-Exertional Malaise at 3-Months, Part 2 (DePaul Symptom Questionnaire, Short Form) Additional Questions **eTable 28.** Post-Exertional Malaise at 3-Months, Part 3 (DePaul Symptom Questionnaire, Short Form) Composite Measures **eTable 29.** 3-Month Follow-up Data: Secondary Outcomes: Accelerometry - Changes From Baseline **eTable 30.** Secondary Outcome Analysis: hospitalization for any Reason (as Recorded in SAEs) **eTable 31.** Visits Involving Physiotherapy and Rehabilitation in the Community **eFigure 1.** The Distribution in the Distances (m) Achieved During the Incremental Shuttle Walk Test at Baseline, 3-Months Post-Randomization, and the Change at 3-Months From Baseline **eFigure 2.** Cohen’s D Forest Plot Depicting Standardized Intervention Effect Estimates and 95% Confidence Intervals for Primary and Secondary Outcome Measures **eFigure 3.** Primary Outcome - Incremental Shuttle Walk Test Distance Intervention Effect estimates From Complier Average Causal Effects Analyses **eFigure 4.** EuroQol-5D-5L Life Utility Score (EQ-5D-5L) **eFigure 5.** Patient Health Questionnaire-4 (PHQ-4) Category **eFigure 6.** Handgrip Strength

Supplement 3.
Data Sharing Statement
