# Supplementary material for: Resistance Exercise Therapy After COVID-19 Infection: A Randomized Clinical Trial
Source: JAMA Netw Open. 2025 Nov 10;8(11):e2534304. doi: 10.1001/jamanetworkopen.2025.34304 (PMC12603858; doi:10.1001/jamanetworkopen.2025.34304)
Supplement: Supplement 1. — Trial Protocol and Statistical Analysis Plan [file jamanetwopen-e2534304-s001.pdf]

Full Title: Prevention and early treatment of the long-term physical effects of COVID-19: a randomised clinical trial of resistance exercise.

Short title: CISCO-21 - Treat and Prevent Long COVID-19

**PROTOCOL VERSION NUMBER AND DATE:**	**Version 4.0; 14.12.2022**
**IRAS Number:**	**294299**
**ISRCTN Number / Clinical trials.gov Number:**	**NCT04900961**
**Sponsors Number:**	**GN20CA537**

This protocol has regard for the HRA guidance and order of content

SIGNATURE PAGE

The undersigned confirm that the following protocol has been agreed and accepted and that the Chief Investigator agrees to conduct the trial in compliance with the approved protocol and will adhere to the principles outlined in the Good Clinical Practice (GCP) guidelines, the Sponsor's (Standard Operating Procedures (SOPs), and other regulatory requirements as amended.

I agree to ensure that the confidential information contained in this document will not be used for any other purpose other than the evaluation or conduct of the clinical investigation without the prior written consent of the Sponsor

I also confirm that I will make the findings of the study publicly available through publication or other dissemination tools without any unnecessary delay and that an honest accurate and transparent account of the study will be given; and that any discrepancies from the study as planned in this protocol will be explained.

**Chief Investigator:**		
Signature:		Date: 14.12.2022
Name: (please print): COLIN BERRY		
Position: PROFESSOR OF CARDIOLOGY AND IMAGING		
**For and on behalf of the Study Sponsor (NHS Greater Glasgow and Clyde):**		
Signature:		Date: 14.12.2022
Name (please print): MAUREEN TRAVERS		
Position: NHS GG&C Research Coordinator		

KEY TRIAL CONTACTS

**Chief Investigator**	**Professor Colin Berry**<br>Imaging and Cardiology<br>BHF Glasgow Cardiovascular Research Centre<br>126 University Place<br>University of Glasgow<br>Glasgow G12 8TA<br>Office: +441413303325<br>Cell: +447831698479<br>Email: colin.berry@glasgow.ac.uk<br><br>Department of Cardiology<br>Queen Elizabeth University Hospital<br>Govan Road, Glasgow
**Trial Co-ordination**	**Project Management Unit**<br><br>Clinical Research and Development<br>Ward 11, Level 1<br>Dykebar Hospital<br>Grahamston Road<br>Paisley, PA2 7DE<br>Contact: Diann Taggart<br>Email: diann.taggart@ggc.scot.nhs.uk
**Sponsor**	**NHS Greater Glasgow & Clyde**<br><br>Clinical Research and Development<br>Ward 11, Level 1<br>Dykebar Hospital<br>Grahamston Road<br>Paisley, PA2 7DE<br><br>Contact: Dr Maureen Travers<br>E-mail: maureen.travers@ggc.scot.nhs.uk
**Data Centre**	**Glasgow Clinical Trials Unit**<br><br>Robertson Centre for Biostatistics<br>University of Glasgow<br>Contact: Sarah Weeden<br>Tel: 0141 330 4744<br>Email: sarah.weeden@glasgow.ac.uk

**Key Protocol Contributors**	**Dr David Anderson**<br>Consultant Respiratory Physician<br>Queen Elizabeth University Hospital,<br>NHS GGC, Glasgow<br>Email: David.Anderson@ggc.scot.nhs.uk<br><br>**Dr Hannah Bayes**<br>Consultant Respiratory Physician<br>Royal Infirmary, NHS GGC<br>Email: Hannah.Bayes@ggc.scot.nhs.uk<br><br>**Mr David Boiskin**<br>Clinical Specialist Physiotherapist<br>Department of Physiotherapy<br>Queen Elizabeth University Hospital<br>Email: David.Boiskin@ggc.scot.nhs.uk<br><br>**Ms Clare Burke**<br>Department of Physiotherapy<br>Queen Elizabeth University Hospital<br>Email: Clare.Burke@ggc.scot.nhs.uk<br><br>**Professor John GF Cleland**<br>Director, Glasgow Clinical Trials Unit<br>Robertson Centre for Biostatistics<br>University of Glasgow<br>Email: John.Cleland@glasgow.ac.uk<br><br>**Professor Carl Goodyear**<br>Professor of Translational Immunology,<br>RB421 Level B4, III - GBRC, University Place, Glasgow G12 8TA<br>Email: Carl.Goodyear@glasgow.ac.uk<br><br>**Dr Stuart Gray**<br>BHF Glasgow Cardiovascular Research Centre<br>126 University Place, Glasgow G12 8TA<br>Email: Stuart.Gray@glasgow.ac.uk<br><br>**Dr Tracy Ibbotson**<br>Room 206, House 1, General Practice & Primary Care, 1 Horselethill Road, Glasgow, G12 9LX.<br>Email: Tracy.Ibbotson@glasgow.ac.uk<br><br>**Professor Chim Lang**<br>Clinical Professor and Consultant Cardiologist<br>Ninewells - Mailbox 2, School of Medicine, University of Dundee, Ninewells Hospital & Medical School DD1 9SY

	Email: C.C.Lang@dundee.ac.uk<br><br>**Professor Alex McConnachie**<br>Robertson Centre for Biostatistics,<br>University of Glasgow, G12 8TA<br>Email: alex.mcconnachie@glasgow.ac.uk<br><br>**Professor Frances Mair**<br>Norie Miller Professor of General Practice and Head of General Practice and Primary Care, University of Glasgow<br>R302A Level 3, General Practice & Primary Care, 1 Horselethill Road, Glasgow G12 9LX<br>Email: Frances.Mair@glasgow.ac.uk<br><br>**Dr Kenneth Mangion**<br>Lecturer in Cardiology, University of Glasgow<br>Queen Elizabeth University Hospital,<br>NHS GGC, Glasgow<br>Email: kenneth.mangion@glasgow.ac.uk<br><br>**Dr Andrew Morrow**<br>MRC Clinical Research Fellow, Institute of Cardiovascular and Medical Sciences, University of Glasgow<br>Queen Elizabeth University Hospital,<br>NHS GGC, Glasgow<br>Email: andrew.morrow@ggc.scot.nhs.uk<br><br>**Professor Naveed Sattar**<br>Institute of Cardiovascular and Medical Sciences,<br>Queen Elizabeth University Hospital,<br>NHS GGC, Glasgow<br>Email: Naveed.sattar@glasgow.ac.uk<br><br>**Dr Robert Sykes**<br>Clinical Research Fellow, Institute of Cardiovascular and Medical Sciences, University of Glasgow<br>Queen Elizabeth University Hospital,<br>NHS GGC, Glasgow<br>Email: robert.sykes@ggc.scot.nhs.uk<br><br>**Professor Rod Taylor**<br>MRC/CSO Social & Public Health Sciences Unit, Institute of Health and Wellbeing, University of Glasgow<br>Email: Rod.Taylor@glasgow.ac.uk

TRIAL SUMMARY

**Study Title**	Prevention and early treatment of the long-term physical effects of COVID-19: a randomised clinical trial of resistance exercise.
**Other Title**	Treat and prevent Long COVID
**Acronym**	CISCO-21
**Study Design**	Randomised, controlled, open-label, parallel group, clinical trial
**Study Participants**	Patients with confirmed COVID-19 recovering in hospital, or having been discharged or not admitted, and with persisting symptoms (4 weeks or more as per NICE guidance).
**Research Question/Aims**	1. To undertake a pragmatic clinical trial of resistance-based exercise in addition to standard care versus standard care only to prevent and treat persisting symptoms post-COVID-19.<br>2. To rapidly translate and disseminate our research findings.<br>3. To develop a national platform for rapid, multicentre clinical trials post-COVID-19.
**Eligibility**	Patients will be classified according by clinical presentation:<br><br>A) Non-hospitalised - Positive diagnosis with persisting symptoms for at least 4 weeks from symptoms onset leading to medical review (A&E, Community COVID Hub) but not admission (Treatment group),<br><br>B) Hospitalised, positive diagnosis, and with post-discharge, persistent symptoms for at least 4 weeks from symptoms onset (Treatment group),<br><br>C) Hospitalised, positive diagnosis, and in convalescent phase in-hospital (Prevention group).<br><br>Groups A & B = target population for *treatment* of persisting symptoms post-COVID i.e. Long-COVID;<br><br>Group C = Target population for *prevention* of Long COVID.<br><br>**Inclusion criteria**<br>1. Virology PCR positive laboratory diagnosis and/or point of care test positive for COVID-19<br>or<br>2. Positive Lateral Flow Test<br>or<br>3. Positive COVID antibody Test<br>4. Within 12months of diagnosis,

	5. Persistent symptoms for at least 4 weeks from symptoms onset (Groups A & B only)<br>6. Presentation type - one of group A, B or C.<br><br>**Exclusion criteria**<br>7. Inpatient physiotherapy currently part of standard care post-ICU<br>8. No expectation of being able to walk within 3 months<br>9. Unable to provide informed consent,<br>10. Unable to comply with the protocol.<br>11. Known pregnancy
**Intervention**	Resistance exercise in addition to standard care
**Control**	Treatment as usual (standard care) without resistance exercise
**Primary outcome**	Incremental shuttle walk test (ISWT) at 3 months post-randomisation
**Secondary outcomes**	1. Spirometry;<br>2. Handgrip strength;<br>3. Short Physical Performance Battery (SPPB)<br>4. EQ5D, PHQ, Illness perception (Brief IPQ), Duke Activity Status Index (DASI), International Physical Activity Questionnaire (IPAQ-SF) short-form, Fatigue questionnaires, MRC dyspnoea score;<br>5. Frailty assessed using 1) the Fried frailty phenotype: five criteria: weight loss; exhaustion; grip strength; low physical activity; and slow walking pace; 2) Clinical Frailty Scale.<br>6. Episodes of care (primary, secondary, physiotherapy, rehabilitation) up to 3 months (Visit 2), and in the longer term<br>7. Hospitalisation for any reason up to 3 months (Visit 2), and in the longer term.
**Exploratory outcomes / sub-studies**	1. Exercise dose achieved (1) daily log, (2) level attained and within subject change from baseline,<br>2. Oxygen saturation, heart rate and respiratory rate at the start and end of the ISWT<br>3. Adherence with exercise (log).<br>4. Accelerometery (Glasgow only)<br>5. Biobank tissues samples for biomedical research.<br>6. Observational sub-study of small vessel function using

	myography in arterioles isolated from gluteal skin biopsies and laboratory analysis of adipose tissue, cells and molecules from the biopsy (optional).
**Planned Sample Size**	220 randomised participants (n=110 per randomised group)<br><br>Stratification of randomisation by clinical categories, A, B and C, history of COVID pneumonia, age, sex and site.
**Planned Trial Period**	24 months<br><br>Pre-start: secure approvals and study set up.<br><br>Week 1: participant enrolment, implementation<br><br>Week 52: mid-point review<br><br>Week 104: final report.<br><br>Record linkage for long term follow up

ROLE OF STUDY SPONSOR AND FUNDER/SUPPORTER

NHS Greater Glasgow & Clyde will be the sponsor of the trial. Sponsor will provide approvals, support regulatory submissions and ensure the requirements of the study are met as per the statement on the signature page of this protocol.

The study is funded through the Chief Scientist Office.

ROLES & RESPONSIBILITIES OF TRIAL MANAGEMENT COMMITTEES/ GROUPS & INDIVIDUALS

Trial Management Group (TMG)

The study will be coordinated from NHS Greater Glasgow & Clyde (NHS GG&C) by the Study Management Group (SMG). The SMG will consist of the chief investigator, project manager and representatives from both NHS GG&C and other organisations relevant to the study. The role of the group is to monitor all aspects of the conduct and progress of the study, ensure that the protocol is adhered to and take appropriate action to safeguard participants and the quality of the trial itself.

Trial Steering Committee (TSC)

The role of the TSC is to provide overall supervision of the trial and ensure that it is being conducted in accordance with the principles of GCP and the relevant regulations. The TSC will:

- review the trial protocol and agree substantial protocol amendments
- provide advice to the investigators on all aspects of the trial
- include an independent chairperson, at least 2 other independent members, and the Co-sponsor and a patient or carer representative

Decisions about continuation or termination of the trial or substantial amendments to the protocol will be the responsibility of the TSC who will advise the sponsor. The TSC will meet at the start of the study, and 6-monthly thereafter. The TSC will have its own charter outlining the role and responsibilities of its members. The TSC may invite other attendees from the trial team to present or participate in discussions on particular topics. These attendees will be non-voting members.

PROTOCOL CONTRIBUTORS

The protocol has been developed by a group with extensive clinical and research experience relevant to this study – our list of contributors can be found on Pages 4 and 5 of this protocol. The team includes internationally leading experts in clinical trials (Berry, Cleland, Lang, Sattar, Taylor), biomarkers (Goodyear, Sattar), pulmonary rehabilitation (Anderson, Bayes), exercise physiology (Gray), primary care (Mair), and biostatistics (McConnachie).

The study will be supported by the Robertson Centre for Biostatistics, University of Glasgow and Clinical Research & Development and the Project Management Unit, NHS GGC.

KEYWORDS

- Coronavirus-19 (COVID-19)
- SARS-CoV-2
- Resistance exercise
- Health status
- Exercise capacity
- Rehabilitation
- Cardiovascular disease
- Clinical trial

LIST OF ABBREVIATIONS

AE	Adverse event
AR	Adverse reaction
BMI	Body Mass Index
CA	Competent authority
CAD	Coronary artery disease
CHI	Community health index
CI	Chief investigator
CRF	Case report form
CRP	C-reactive protein
CT	Computed tomography
CTCAE	Common Terminology Criteria for Adverse Events
CTPA	Computed tomography pulmonary angiography
CTU	Clinical trials unit
CXR	Chest X-ray
DASI	Duke Activity Status Index
ECG	Electrocardiogram
eGFR	Estimated Glomerular Filtration Rate
EQ5D	European quality of life 5-domain
EU	European Union
GCP	Good clinical practice
GDPR	General data protection regulation
GP	General Practitioner
ICAM	Intercellular adhesion molecule-1
ICF	Informed consent form

ISWT	Incremental shuttle walk test
ICMJE	International Committee of Medical Journal Editors
ICH GCP	International Conference on Harmonization of Good Clinical Practice

IPQ	Illness perception questionnaire
ISF	Investigator site file
LPLV	Last Patient Last Visit
LV	Left ventricle
MI	Myocardial infarction
MRC	Medical Research Council
NHS	National Health Service
NHS GGC	National Health Service Greater Glasgow & Clyde
NHS R&D	National Health Service Research & Development
NICE	National Institute for Health and Care Excellence
NT-proBNP	N-terminal pro-brain natriuretic peptide
PCR	Polymerase chain reaction
PHQ-9	Patient health questionnaire - 9
PPI	Patient and Public Involvement
PROMS	Patient reported outcome measures
RCPGP	Royal College of General Practitioners
REC	Research Ethics Committee
SAE	Serious adverse event
SDV	Source data verification
SIGN	Scottish Intercollegiate Guideline Network
SMG	Study management group
SOP	Standard Operating Procedure
SOT	System organ term
SSI	Site specific information
RUSAE	Related Unexpected Serious Adverse Event

SUSAR	Suspected Unexpected Serious Adverse Reaction
TIA	Transient ischaemic attack
TMF	Trial master file
UK	United Kingdom
ULN	Upper limit of normal
WHO	World Health Organisation

TABLE OF CONTENTS

SIGNATURE PAGE 2
TRIAL SUMMARY 6
FLOW DIAGRAM 16
SCHEDULE OF ASSESSMENTS 17
STUDY PROTOCOL 19
1. BACKGROUND 19
2. RATIONALE 21
3. RESEARCH QUESTION / AIM(S) 23
4. STUDY DESIGN 25
5. SETTING 25
6. STUDY POPULATION 26
7. TRIAL PROCEDURES 27
8. SAFETY REPORTING 38
9. Statistical Analysis 42
10. DATA HANDLING 44
11. MONITORING, AUDIT & INSPECTION 44
12. ETHICAL AND REGULATORY CONSIDERATIONS 45
13. DISSEMINATION POLICY 49
14. REFERENCES 50
15. APPENDICES 52

FLOW DIAGRAM

CONSORT
TRANSPARENT REPORTING of TRIALS

CONSORT 2010 Flow Diagram

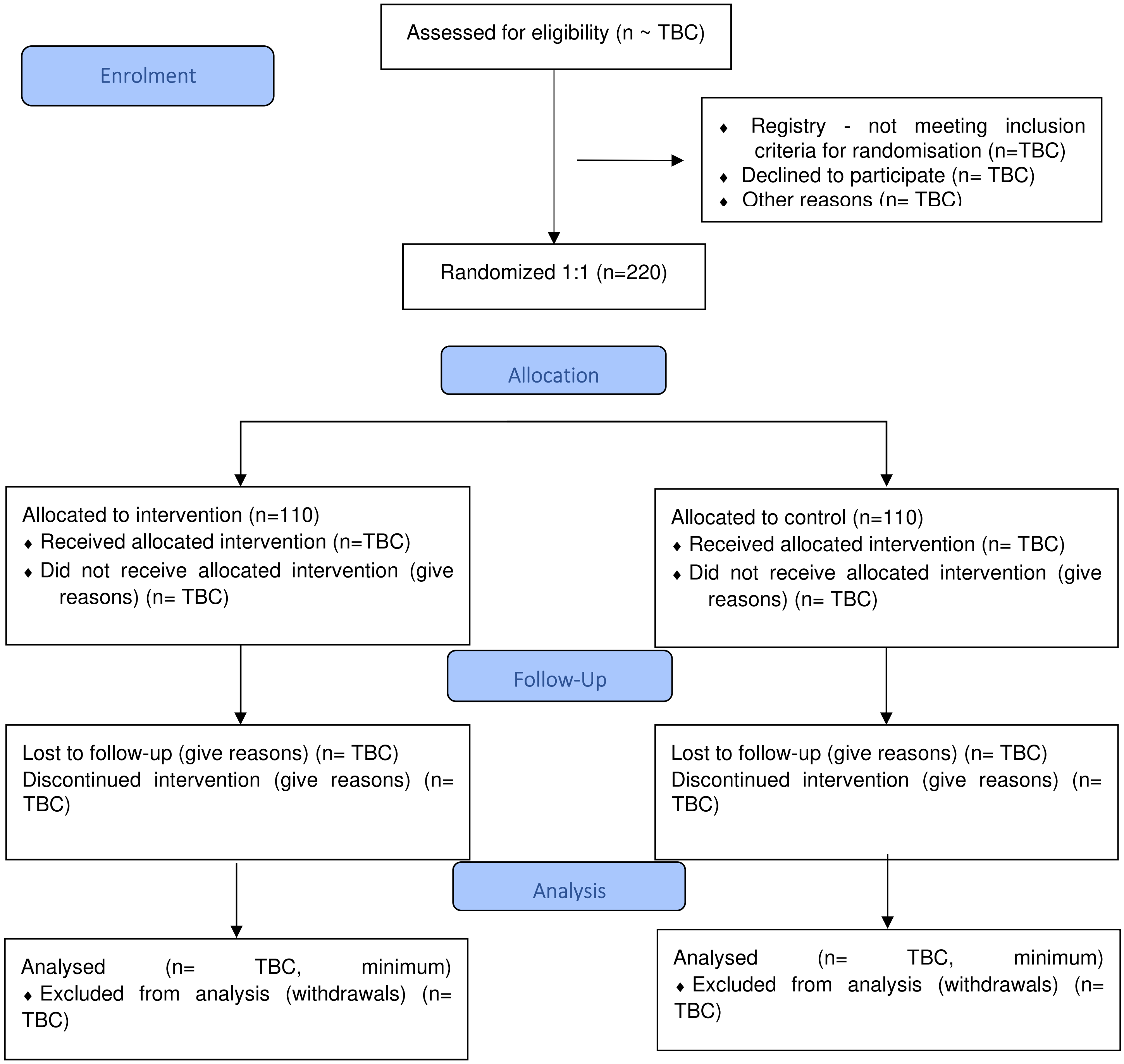

SCHEDULE OF ASSESSMENTS

Visit	1	2	3
Timeline	Day 1	3 months (+6weeks)	E-linkage
Trial Activity<br><br>Setting	Enrolment<br><br>Hospitalised, non-hospitalised COVID-19	Outcome evaluation CRF	1 & 10 years
Screening – Inclusion/Exclusion Criteria including DASI questionnaire	√		
Written Informed Consent	√		
General health status check	√		
Medical History / clinical status[2]	√	√	
Vital signs (heart rate, rhythm, BP, height, weight, waist circumference, oxygen saturation, at baseline and during follow-up)	√	√	
Cardiovascular risk factors, risk score	√	√	
Routine blood samples as per standard of care	√	√	
Spirometry	√	√	
Handgrip strength	√	√	
Short Physical Performance Battery (SPPB)	√	√	
Accelerometer (Glasgow only)	√	√	
PROMS including EQ5D, PHQ, Illness perception (Brief IPQ), Duke Activity Status Index (DASI), International Physical Activity Questionnaire (IPAQ-SF) short-form, Fatigue questionnaires, DSQ Short Form PEM Questionnaire	√	√	
Frailty 1) Fried 5-criteria phenotype, 2) Clinical Frailty Scale	√	√	
Training - Hospital, CRF, on-line, telephone			
Randomisation	√		
Training (resistance exercise)	√		
Incremental shuttle walk test (ISWT)	√	√	

Exercise dose (log, level, adherence)	√	√	
Episodes of care (primary, secondary, physiotherapy, rehabilitation)		√	√
Research blood sample		√	
Gluteal Biopsy[6]		√	
Data collection – clinical endpoints (collected via hospital Portal/Trakcare systems) and e-record linkage			√

All visits should be performed within the documented visit time (e.g. 3 months +6 weeks). The aim is to meet these visit windows but any that are out-with will be dealt with at the analysis stage, as appropriate, and therefore will not be considered protocol deviations. If the visit date is extended beyond 3 months, the participant should continue with the exercise regimen until the day of the visit.

1. Informed consent must be obtained before the patient undertakes any study assessments.
2. Clinical activity is defined as 'Standard Care'(including safety), 'Research', or for the UK NHS, 'NHS Support', 'NHS Treatment'
3. The research blood sample should be undertaken at the 3-month visit.
4. The participants will be contacted by the Research Staff by telephone one week after enrolment, regardless of the randomised group in order to assess patient wellbeing and adherence with the resistance exercise intervention. If there are concerns, then the participant will be invited to the Clinical Research Facility for review.
5. Consent covers the randomised study and participation in an optional follow-up using electronic linkage of NHS and government records for episodes of care and death (and their causes) and medication in the longer term.
6. All participants who have passed the 3 month time point may be contacted to be given the opportunity to participate in the sub-study.

Standard care blood samples e.g. FBC, U&E, HbA1c, D-Dimers, NTproBNP, lipids, CRP, high sensitivity troponin

Research blood samples: Exploratory analyses may include - Haemostasis - Claus Fibrinogen, D-Dimer, FVIII (one stage), VWF antigen, VWF:GP1ba, Antithrombin activity, Protein C activity, Free protein S; Cardiac - NT-proBNP, hs-troponin; Vascular cytokines - ET-1, ICAM, VCAM, p-selectin, IL-6, VWF, Nox3; Vascular cells and microparticles; Metabolic status - HbA1C, lipids; Blood RNA (Tempus™ or PAXgene®) including for RT-PCR of SARS-CoV-2 and RNA for vascular biology; Inflammation – hs-CRP, ST2; Immune response antibodies; Collagen metabolites; DNA - The buffy coat will be stored for DNA analysis e.g. candidate gene SNPs, other variants. Residual blood will be saved for future analyses of interest. These analyses will be undertaken in University laboratories or in laboratories of external collaborators (industry or academic, UK or abroad) by agreement with the Sponsor.

STUDY PROTOCOL

Prevention and early treatment of the long-term physical effects of COVID-19: a randomised clinical trial of resistance exercise.

1. BACKGROUND

The COVID-19 pandemic is having an unprecedented impact on our health and social services, and on the population, society, and the economy [1,2]. COVID-19 is a systemic illness, and complications include pneumonia and pulmonary thromboembolism [3]. In our CSO-funded Cardiovascular Imaging in SARS-CoV-2 (CISCO-19) study [4; pilot data], most hospitalised patients experience persisting health problems after the acute illness. COVID-19 is associated with an increase in cardiovascular risk [3-5], that potentially may be reduced by evidence-based cardiovascular interventions. A report in the BMJ (May 2020) from the ISARIC-4C framework described the results for 17,702 patients admitted to 208 acute hospitals in the UK, including several hospitals in NHS Scotland [3]. A history of cardiac disease was among the most common morbidities (31%, 5469/17702), and a multivariable predictor of all-cause mortality (hazard ratio (95% confidence interval) 1.16 (1.08 to 1.24); $p<0.001$). These results provide a strong link between cardiovascular disease and the prognosis of COVID-19.

Systemic inflammation, direct infection of endothelial cells by SARS-CoV-2 and dysfunction of angiotensin converting enzyme-2 (ACE2), cause endothelial dysfunction (aka 'endothilitis') [4,6,7]. Consequent microvascular dysfunction, thrombosis, and occlusion likely play a key role in multi-organ dysfunction, especially in the lung, heart, and kidney. A key objective of CISCO-19 is to assess coronary microvascular function [4]. We have found that myocardial injury, such as might occur due to myocarditis, is uncommon [4, pilot data]. On the other hand, impairments in blood flow in the heart appear to affect around 1 in 3 patients, and estimated oxygen consumption (VO_{2max}), a measure of aerobic exercise capacity, is persistently reduced during the convalescence phase (1 month) [4]. Bringing these findings together with the current published evidence, COVID-19 patients have a risk of persisting symptoms, coronary and systemic microvascular dysfunction may be an underlying mechanism for persisting exertional breathlessness, chest pain, and fatigue [4-7].

COVID-19 patients may experience a loss of metabolically active tissue [8]. Hospitalisation may be prolonged, and they are at risk of substantial weight loss, including sarcopenia and cachexia [9]. The mechanisms of cachexia are multifactorial and include tissue catabolism secondary to the acute infection, systemic inflammation, cytokine activation, and loss of mobility [10]. These problems may be especially relevant in older patients with COVID-19, and those with pre-existing chronic health problems, of whom there are many. Even a short period of immobilisation, in-hospital or at home, bed rest and the inactivity of sustained quarantine can have clinically relevant effects on muscle and metabolic health. A reduction in physical activity for just 2 weeks can result in significant losses in muscle mass and function [11] and peripheral insulin sensitivity [12]. A systematic review has identified generalised impairments in physical function after COVID-19 [13]. This is important, as muscle mass is a strong predictor of prognosis in many chronic conditions, and frailty, which associates with multimorbidity and socioeconomic deprivation, may be especially relevant [14].

Pharmacological methods to increase muscle mass are few and the main method to improve muscle mass and function is via resistance exercise. We have recently developed a resistance exercise intervention [15] which we have employed in people with type 2 diabetes (see pilot data). On top of its effects on muscle mass and function, which are not seen with other forms of exercise, resistance exercise is pragmatic in COVID-19 as it can be easily adapted to the patients' capabilities and

performed in patients from the spectrum of bed-bound to ambulatory with the need for little equipment. Resistance exercise has similar broad ranging health benefits, such as reducing blood pressure and improving glycaemic control (PMID: 30045740; 30969369) and be associated with a reduction in CVD risk (PMID: 30376511). Importantly for this study, resistance training during periods of reduced activity can attenuate the declines in muscle mass and function (PMID: 26265753).

Many patients who have survived COVID-19 describe persisting health problems. NICE, the Scottish Intercollegiate Guidelines Network (SIGN) and the Royal College of General Practitioners (RCGP) have defined Long COVID as 'signs and symptoms that develop during or following an infection consistent with COVID-19 which continue for more than 12 weeks and are not explained by an alternative diagnosis' [16]. Using clinical data from the over four million people who downloaded the COVID Symptom Study app [17] developed by the health science company, ZOE, Spector and colleagues at Kings College London have provided insights into persisting health problems of COVID-19 patients who were not admitted to hospital. Their preliminary and, arguably, somewhat 'crude' data show that more than 1 in 8 individuals still have problems after one month from the date of diagnosis, around 1 in 20 have persisting symptoms at two months and 1 in 50 have symptoms at 3 months. Given the scale of the COVID-19 pandemic, many thousands of individuals will be affected. These Long COVID sufferers reported a range of symptoms, including up to 20 different symptoms beyond those used for the initial diagnosis of acute infection. These results underline that COVID-19 is a multisystem disease that requires a multidisciplinary approach to treatment.

Since SARS-CoV-2 is a new virus, the initial research focus has been to investigate the acute illness through observational studies and trials of acute therapy. In the CSO Rapid Research programme, we secured funding for the CISCO-19 heart, lung, and kidney imaging study (ClinicalTrials.gov Identifier: NCT04403607) based in the West of Scotland [4]. This on-going study involves recruiting 160 patients in hospital, imaging at 28 days post-discharge and then again at 1 year. The study involves stress perfusion cardiovascular magnetic resonance (CMR) to quantify myocardial blood flow, and CT coronary angiography (CTCA). Patient reported outcome measures (PROMS), covering symptoms, quality of life, and functional status), and blood samples are being collected at each visit. COVID-HEART, a national BHF-NIHR Flagship study, was modelled on CISCO-19. These, and other BHF-NIHR studies, will describe the natural history of COVID-19. ISARIC-4C is a UK-wide, secondary care COVID-19 study [3]. PHOSP is a national consortium funded by the UKRI studying long-term health outcomes in patients who have been hospitalised for COVID-19. PHOSP involves 3 tiers, including routine clinical data (Tier 1), research assessments measured at standard care clinic visits (Tier 2; n=4,000 patients) and de novo, linked studies (Tier 3). The PHOSP Tier 2 assessments may occur at 6 weeks (phone call), 3 months (the first face-to-face consult), and 6 and/or 12 months, where clinically indicated. In the main, these national flagship projects have focused on characterisation of the disease and implementing frameworks to support research. To date, randomised trials have focused on the acute phase of the illness. They include RECOVERY (NCT04381936), TACTIC-E (NCT04393246) and DEFINE (NCT04473053).

Clinical management of patients following COVID-19 in the NHS will generally align with practice guidelines from the British Thoracic Society and other key stakeholders such as NICE, RCPGP and SIGN. Local practice may vary according to circumstances. In NHSGGC, patients who have been admitted with radiological and/or virology confirmed COVID-19 would be invited for a CXR 12 weeks post-discharge and if there are persisting abnormalities on the X-ray, the patient would be invited to a phone consultation. Patients would attend out-patient clinics according to clinical need and logistical considerations.

As we learn more about the natural history of COVID-19, it is clear that many patients (millions worldwide) are left with persisting symptoms such as exercise intolerance, breathlessness, fatigue,

[3,4,17] that may represent persisting dysfunction of the heart, lungs, peripheral tissues, and their microcirculation [3,4]. It is also possible that COVID-19 was just an intercurrent event that interacted with and exposed an underlying health problem (e.g. chronic lung disease, or iron deficiency). A post-viral syndrome that might be anticipated to affect some patients, as is the case with other viral illnesses, is also relevant. NICE, SIGN and RCGP have highlighted the paucity of randomised trial evidence to show whether treatment of the convalescent phase after COVID-19, with or without hospitalisation, reduces longer-term disability [16]. We believe there is a knowledge-gap relating to the prevention and early treatment of longer-term health problems post-COVID-19. The recent CSO call into the long-term effects (LTE) of COVID-19 presents an ideal opportunity to build on CISCO-19. We will develop a clinical trial which in turn will set the framework for additional, linked clinical trials for national implementation across NHS Scotland, and potentially the UK. Our CSO LTE trial proposal complements our parallel application for BHF-NIHR flagship prioritisation for a "COVID-19 Rapid Cardiovascular Trials Platform" (under review, submission October 30, 2020).

2. RATIONALE

Many people have long-lasting symptoms after COVID-19, such as breathlessness, fatigue and chest pain. So far, research studies of treatments for COVID-19 have focused on the life-threatening acute illness; few studies look at treatments to improve long-term health after COVID-19. COVID-19, particularly when this requires a hospital admission, can lead to weight loss and muscle wasting, contributing to worse outcomes. Muscle strengthening (resistance-based) exercise could improve outcomes in the long-term.

The rationale for our study has two main parts. The first (primary) addresses a gap in therapy, notably, for a non-pharmacological intervention. To address this gap, we have set out to develop a lifestyle intervention that may be helpful to patients with persisting symptoms in the recovery (or convalescence) phase after COVID-19. Specifically, we will train participants to undertake a pragmatic resistance-based exercise intervention that they can learn and apply according to their circumstances in-hospital or in the community. The rationale is predicated on providing patients with a personalised therapy option and empowering them in the self-management of their recovery following illness due to COVID-19 infection.

The second area of need is the general lack of evidence-based medicines for patients who have persisting symptoms after COVID-19. To address this gap, there is a need for rapid trials to evaluate multiple therapeutic candidates to urgently provide doctors (and their patients) with clinical evidence to inform treatment decisions. To address this gap, we aim to create a platform for rapid trials of new treatments after COVID-19. We wish to seize the opportunity afforded by this trial to set-up a transferable framework for future trials in COVID-19. The rationale is to provide a low-cost trials resource to facilitate the rapid set-up and cost-efficient delivery of multiple other trials. This will be a parallel work strand during the trial. We will develop a Working Group of stakeholders from across the NHS and Universities in Scotland and our COVID-19 PPI Group (which includes co-applicants and collaborators). Key considerations include repurposing novel therapy, potential for efficacy, safety, endpoint, sample size, feasibility interim analyses, power, MHRA and costs. As part of the plan of work in application (Aim 3), the researchers and PPI Group will scope these potential interventions within the context of a platform trial with a view to identifying candidates to follow-on after the exercise intervention trial. This will cut the costs of doing future trials and allow more patients the opportunity to contribute to medical research that will improve outcomes for people recovering from COVID-19.

Our team is multidisciplinary, multi-ethnic, gender-balanced and drawn from across NHS Scotland, and the University of Glasgow. We are currently leading the CISCO-19 study (ClinicalTrials.gov Identifier: NCT04403607), funded by the CSO, in the West of Scotland, using medical imaging of the hearts, lungs, and kidneys of patients, to understand the impact of COVID-19 [4]. We have consulted with members of the public, including people with COVID-19, in designing this study.

We will also undertake exploratory research into the vascular biology of COVID-19 infection. This work will be undertaken in collaboration with scientists in the University of Glasgow, including ICAMS and the MRC Centre for Virus Research. A blood sample at 3 months will be collected and stored in the NHS Biorepository.

In order to assess the natural history, longer-term follow-up for health outcomes will be undertaken using electronic record linkage to patient records omitting the need for participants to undergo further research visits after the end of the trial. If after initial analysis longer term, follow up via national records is felt to be worthwhile additional funding will be sought for this.

Alignment with other national / international initiatives

Our Tier 3 PHOSP proposal is intended to link with COVID-HEART and other studies in NHS Scotland that include consent to be contacted about future research. Co-enrolment would be desirable to synergise the studies. If funded, our trials programme will bring in new resource to enhance enrolment into existing studies, specifically, by engaging more sites, and more research staff. The primary outcome of the exercise intervention coincides with the Tier 2 visit in PHOSP. Our consortium includes the lead investigators of BHF-NIHR prioritised studies. Prof Berry contributed to the concept and protocol of COVID-HEART. He is PI for CISCO-19, a Trial Steering Committee member of PHOSP and Co-Local PI for CAPACITY-COVID.

Future trials

Our proposal will create a trials framework ready for use in real-time with the potential to extend and develop contingent on future research funding. Having set up the multicentre clinical trial, we wish to make available the framework to support other interventions (see below). They are anticipated to include interventions for which substantial safety data already exist but for which efficacy data are lacking in this patient population. Developing these new trials, from concept through to funding, is a specific aim that is nested within the current proposal. This plan builds on the existing infrastructures already in use for CISCO-19 and other COVID-19-related research developed by clinical researchers at the Universities of Glasgow and Dundee. The proposal will link with existing, national research studies including the BHF-NIHR prioritised COVID-HEART and PHOSP studies in which the CI (Berry) already has leadership roles (Co-Lead, COVID-HEART; TSC Member, PHOSP), and potentially facilitate a UK wide trials platform. Our vision is to provide other researchers with an eCRF that is already immediately available for use. The eCRF platform would then be customised to the needs of the study. This approach should facilitate rapid set up of multiple new trials and greatly reduce costs.

3. RESEARCH QUESTION / AIM(S)

3.1 Objectives

1) To undertake a clinical trial of pragmatic resistance-based exercise in addition to standard care versus standard care only to prevent and treat persisting symptoms post-COVID-19.
2) Rapidly translate and disseminate our research findings.
3) Develop a national platform for rapid, multicentre clinical trials post-COVID-19.

3.2 Questions

1. **Does resistance-exercise intervention improve functional capacity and health status post-COVID-19?**
   Hypothesis: A personalised yet pragmatic approach to resistance-based exercise as an adjunct to standard care improves health status and functional capacity, as compared to standard care without resistance-based exercise.

2. **Do the results of this trial elucidate mechanisms of disease in Long COVID?**
   Hypothesis: Improvements in functional capacity with resistance-based exercise identify ***reversible impairments*** in skeletal muscle function in patients after COVID-19.

3. **Is a national clinical trials platform for post-COVID patients in NHS Scotland feasible?**
   Hypothesis: A clinical trials infrastructure permits the rapid implementation of novel therapeutic interventions in patients with Long COVID.

3.3 Primary outcome

Incremental shuttle walk test (ISWT) at 3 months.

Incremental shuttle walk test (ISWT) [20]. This is a validated measure of functional capacity, with test-retest reliability and evidence of being responsive to rehabilitation interventions. The ISWT is used as a Tier 2 evaluation in the PHOSP-COVID study. By adopting the ISWT as the primary outcome measure, our study will complement PHOSP, align with the protocol (for those patients who have been enrolled), and potentially, enhance co-enrolment into this observational study.

Oxygen saturation, heart rate and respiratory rate will be measured at the start and end of the ISWT. They will be exploratory outcomes.

3.4 Secondary outcomes

1. Spirometry;
2. Handgrip strength;
3. Short Physical Performance Battery (SPPB)
4. EQ5D, PHQ, Illness perception (Brief IPQ), Duke Activity Status Index (DASI), International Physical Activity Questionnaire (IPAQ-SF) short-form, Fatigue questionnaires, MRC dyspnoea score;
5. Frailty assessed using 1) Fried frailty phenotype: five criteria: weight loss; exhaustion; grip strength; low physical activity; and slow walking pace; 2) Clinical Frailty Scale
6. Episodes of care (primary, secondary, physiotherapy, rehabilitation)
7. Hospitalisation for any reason.

The secondary outcomes are intended to align with PHOSP assessments at 3-months.

3.5 Exploratory outcomes

1) Exercise dose achieved (1) daily log, (2) level attained and within subject change from baseline,
2) Vital parameters of cardio-respiratory function e.g. oxygen saturation, heart rate, respiratory rate at baseline and during follow-up
3) Adherence with exercise (log)
4) Accelerometery (Glasgow site)
5) Biobank tissues samples for biomedical research
6) Observational sub-study of small vessel function using myography in arterioles isolated from gluteal skin biopsies and laboratory analysis of adipose tissue, cells and molecules from the biopsy (optional).

Research blood samples: Exploratory analyses may include - Haemostasis - Claus Fibrinogen, D-Dimer, FVIII (one stage), VWF antigen, VWF:GP1ba, Antithrombin activity, Protein C activity, Free protein S; Cardiac - NT-proBNP, hs-troponin; Vascular cytokines - ET-1, ICAM, VCAM, p-selectin, IL-6, VWF, Nox3; Vascular cells and microparticles; Metabolic status - HbA1C, lipids; Blood RNA (Tempus™ or PAXgene®) including for RT-PCR of SARS-CoV-2 and RNA for vascular biology; Inflammation – hs-CRP, ST2; Immune response antibodies; Collagen metabolites; DNA - The buffy coat will be stored for DNA analysis e.g. candidate gene SNPs, other variants. Residual blood will be saved for future analyses of interest.

4. STUDY DESIGN

This trial has a prospective, randomised, open-label, parallel group design. The trial is designed to assess the superiority of resistance-exercise intervention as compared with standard care without this intervention in patients recovering after COVID-19.

5. SETTING

All patients presenting at participating hospitals and Primary Care settings e.g. community assessment centres (CAC), Community Health Centres (CHC) or Community Test Centres (CTC), with COVID-19 can be considered for inclusion. Also, patients enrolled in CISCO-19, OBELIX, RECOVERY and ISARIC studies, and other relevant studies, who fulfil the inclusion/exclusion criteria for CISCO-21, with the permission of the study CI and local PI, may be approached re co-enrolment in the CISCO-21 study. A screening log with the reasons for not being enrolled, will be prospectively completed.
We estimate recruiting participants from existing networks (e.g. Long COVID Scotland groups) from primary care. Recruitment from GP practices will be facilitated by providing information about the study i.e. poster, patient information sheet, consent form, and NRS Primary Care Network using: electronic records search; mail/text invitations; alerts for prospective/opportunistic identification.

Research collaborators include, in the University of Glasgow, the Institute of Cardiovascular and Medical Sciences, the MRC-University of Glasgow Centre for Virus Research, the Robertson Centre for Biostatistics and the Electrocardiography Core Laboratory.

5.1 Standard care

Clinical management of patients following COVID-19 in the NHS will generally align with practice guidelines from the British Thoracic Society and other key stakeholders such as NICE, RCPGP and SIGN. Local practice may vary according to circumstances. In NHSGGC, patients who have been admitted with radiological and/or virology confirmed COVID-19 would be invited for a CXR 12 weeks post-discharge and if there are persisting abnormalities on the X-ray, the patient would be invited to a phone consultation. Options for the evaluation of patients with persisting symptoms include to provide an oxygen saturation monitor, a functional assessment of physical capacity, and/or an invitation to attend an out-patient clinic according to clinical need and logistical considerations.

STUDY POPULATION

6.1 Clinical strata

Since COVID-19 is an acute illness with a recovery period that may be prolonged, we recognise that different approaches are needed to reflect the stage and trajectory of recovery from COVID-19. Patients will be classified according to the clinical management of their illness:

A. Non-hospitalised - Positive diagnosis with persisting symptoms leading to medical review (A&E, Acute Medical Care, Community COVID Hub, General Practice) but not admission,

B. Hospitalised, positive diagnosis, post-discharge, persistent symptoms,

C. Hospitalised, positive diagnosis, convalescent phase in-hospital.

Groups A & B = target population for treatment of persisting symptoms post-COVID i.e. Long-COVID; Group C = Target population for prevention of Long COVID.

6.2 Inclusion criteria

1. Virology PCR positive laboratory diagnosis and/or point of care test positive for COVID-19
2. Positive Lateral Flow Test
3. Positive COVID antibodyTest
4. Within 12 months of diagnosis,
5. Persistent symptoms for at least 4 weeks from symptoms onset (Groups A & B only)
6. Presentation type - one of group A, B or C;

6.3 Exclusion criteria

1. Inpatient physiotherapy currently part of standard care post-ICU*,
2. No expectation of being able to walk within 3 months
3. Unable to provide informed consent,
4. Unable to comply with the protocol.
5. Known pregnancy

NB Since the primary outcome is the ISWT at 3 months, a patient who will not be able to walk during the follow-up period would therefore not be able to complete the primary outcome evaluation. Accordingly, a patient who will be unable to walk should not be enrolled. Patients who are 'bed-bound' may be enrolled provided there is a reasonable belief that they will be able to walk 3 months later.

*Rehabilitation is part of standard NHS care and this is complementary to the study protocol. Active, ongoing physiotherapy for inpatients who have been treated in ICU is an exclusion criterion. Once active, inpatient physiotherapy has completed the patients become eligible to take part.

6.4 Stratification of randomisation

Participants who fulfil eligibility criteria and provide written informed consent will have randomisation stratified by clinical strata groups (A – C), history of COVID-19 pneumonia (yes / no), age, sex and site.

7. TRIAL PROCEDURES

Please see Schedule of Assessments

7.1 Screening and recruitment

Screening will be undertaken in A&E, the Acute Medical Assessment Units and medical wards. Patients who attend Community Assessment Centres, Community Test Centres or Health Centres and have a diagnosis of COVID-19 confirmed may also participate. To this end, we will also search general practice records using the NRS Primary Care Network to identify people with a history of COVID-19 and virology PCR positive laboratory confirmation. Our screening strategy includes the option to contact individuals who have indicated a willingness to be contacted for future research through SHARE (https://www.registerforshare.org/) and Test and Trace. The Test and Trace data are held in the NHS Safe Havens in NHS Scotland. Access to the data is controlled to minimise contact with participants.
There will also be a dedicated webpage/site for the study that patients can use to contact the study team and learn a bit more about the study. There will be a study-specific email address coordinated by the University of Glasgow. The email is intended to serve as a point of contact for individuals interested to learn more about the study and to facilitate communication with the research team. The email address will be included on the website and in communications about the study e.g. Twitter, in e-newsletters e.g. NHS, University, and in the poster.

Patients with a history of COVID-19 who have been discharged following this episode of care may also be sent the PIS and consent form. These centres as well as General Practices may display posters about the study such that the patients may contact the research centre if their symptoms persist during their convalescence. Posters may also be placed in public areas in the hospitals and community health centres.

A screening log will be prospectively recorded and entered into the eCRF.

Patients who are enrolled in observational studies may be eligible to participate. For example, the Cardiovascular Imaging in SARS-CoV-2 (CISCO-19) study is an observational cohort study and patients are invited to give informed consent to be contacted about future studies. Therefore, patients who have been enrolled into CISCO-19 and who fulfil eligibility criteria may be eligible to participate in this next-stage clinical trial.

7.2 Informed consent

Only patients who are sufficiently well to understand the information about the study (including the potential for benefit and known risks), as described by the attending clinical research nurse and/or clinician, would be eligible to participate. Potential participants will be identified and screened using the clinical inclusion and exclusion criteria defined in this protocol, and approached as follows: Patients in

Group A - Non-hospitalised, with symptoms persisting for at least 4 weeks post diagnosis: Potential participants in this patient group will be identified and invited to take part in the study when they attend A&E or Community Assessment Centre, Community Test Centre or Community Health Centre with persistent symptoms. They can be given a PIS at this visit, or agree for their contact details to be passed on the research team and a PIS sent out to them. Alternatively, they may be identified at a later date from review of records by the usual care team and sent a letter of invitation and a PIS at this point. Or such patients may be identified via searching of general practice records by the primary care NRS network who utilize Docmail Services to print, collate and send out invitation letters and patient information leaflets inviting patients to contact the research team directly, either by mail, email or telephone, to express an interest in participating.

Group B- hospitalised with COVID-19, with symptoms persisting for at least 4 weeks post diagnosis, and discharged. Potential participants will be approached by their usual care team re the study pre – discharge, or agree for their contact details to be passed to the research team and a PIS sent out to them. Again. if patients are identified as potentially eligible from a review of records at a later date the can be sent an invitation letter with a PIS for the study with contact details of the research team.

In groups A and B we would also like to be able to follow up the letter with a phone call after 2 weeks to see how patients are and if are interested in joining the study

Group C- hospitalised with COVID-19 and convalescent in hospital: Potential participants would be approached by a member of their usual care team re taking part in the study and if interested asked to agree to speak to a member of the research team about this.

Potential participants for all groups will be given as much time as they need to decide whether to take part in the study.

In all cases clinical staff will be available to discuss the study with the patient and his/her family and friends. If the patient agrees to take part in the study, then the consent form will be signed by the patient and the member of staff obtaining the consent. The decision to include a patient in the study will be documented in the medical notes. No further scheduled study assessments can be performed until written informed consent has been obtained.

Withdrawal criteria: A participant may withdraw from the study at any time. There are no specific withdrawal criteria, although clinicians can withdraw patients as appropriate and record the reasons. All patients will be followed up for clinical outcomes, unless consent is specifically withdrawn.

Participants consenting for the study will be invited to provide consent for long-term follow-up of their electronic medical records (with no additional patient contact). Consent will be sought to use study data in other ways such as in pooled analyses of anonymised data such as for natural history studies, meta-analyses and health outcomes research.

Gluteal biopsy sub-study

The patients who attend Visit 3 will be invited to take part in a sub-study of small blood vessel function. The patients will be given the information sheet at the time of the visit, or if this is omitted e.g. for logistical reasons, then the information sheet and consent form will be sent to their home address. Patients who might be interested to take part will have the opportunity to discuss the sub-study with one of the doctors. This discussion may take place in the Clinical Research Facility or by telephone, according to whatever is most convenient for the individual.

7.3 Recruitment plan

Healthcare staff who participate in this study should adopt a consecutive approach to enrolment in order to minimise selection bias and provide a study cohort that is representative of patients treated during usual care. The results are intended to have external relevance and be transferable to clinical practice. This approach will also facilitate timely delivery of the trial.

The sample size estimate takes account of a degree of participant withdrawals from the study, incomplete data, and problems with data acquisition.

7.4 Clinical evaluation

7.4.1 Clinical status

Information on medical history and COVID-19 will be collected. The information will be collected at baseline and again at 3 months. The information will as far as possible align with the CISCO-19 study (Appendix), and will include:

1. Cardiovascular history
2. Medical history/therapy, including history of COVID-19 pneumonia and physiotherapy
3. Vital signs and anthropometry (heart rate, rhythm, blood pressure, height, weight, waste circumference, oxygen saturation)
3. Comorbidity including the components of the Charlson Index
4. Scottish Index of Multiple Deprivation derived from the post code
5. Cardiovascular risk factors e.g. smoking history, cardiovascular risk scores.
6. Respiratory history – Asthma, bronchiectasis, COPD (including bronchitis and emphysema), chronic rhinosinusitis, hypersensitivity pneumonia, lung cancer, lung fibrosis, chronic pleural disease, pneumoconiosis, pulmonary eosinophilia, pulmonary vascular disease (PTE, pulmonary hypertension, cor pulmonale), sarcoidosis, sleep apnoea syndrome), other *WHO Common Chronic Respiratory disease list.
7. Post-COVID lung disease (convalescence) - fibrosis, pulmonary hypertension, PTE

8. COVID-19 – diagnosis and treatment, vaccination status, reinfection(s) (dates, hospitalisation Y/N)
9. 12-lead ECG (if available)
10. Imaging (if available) – CXR, CT chest / CTPA, cardiac
11. COVID-19 prognostic risk score e.g. ISARIC-4 and WHO severity score.

Smoking status will be defined as per established definitions: https://www.cdc.gov/nchs/nhis/tobacco/tobacco_glossary.htm

7.4.2 Clinical strategy

For the purposes of classifying sub-groups within the target population, participants will be categorised into one of 3 groups

A) Non-hospitalised - Positive diagnosis with persisting symptoms leading to medical review (A&E, SATA, Community COVID Hub) but not admission,

B) Hospitalised, positive diagnosis, post-discharge, persistent symptoms at 4 weeks or longer (PHOSP contact),

C) Hospitalised, positive diagnosis, convalescent phase in-hospital.

Groups A & B = target population for treatment of persisting symptoms post-COVID i.e. Long-COVID; Group C = Target population for prevention of Long COVID.

7.5 Blood Sampling

7.5.1 Sample collection

Samples required for medical management will at all times have priority over samples taken for research. Wherever possible, taking additional research blood samples should be timed to coincide with routine clinical sampling time-points.

Sampling time-points:

Visit 1 - Enrolment (post-consent), standard care bloods (if not available),

if omitted at Visit 1,

Visit 2 – 3 months + 6 weeks following enrolment

Blood samples required at each timepoint: standard care and research samples – see footnote, Schedule of Events.

Blood samples (25 ml) for central laboratory tests of circulating molecules implicated in the pathophysiology acute MI or myocardial injury, potentially complicated by COVID-19. Sample collection kits will be provided for these research bloods, including PAXgene tubes. Samples will be

collected and dealt with as per study Sample Handling Manual. These samples will be batched and analysed in line with available funding.

Routine standard of care bloods will be assayed in local NHS laboratories as per standard procedures irrespective of site. Recent publications describe cardiovascular complications leading to hospital readmissions post-COVID-19 (Ayoubkhani et al (2021); the OpenSAFELY study (Tazare J et al 2021), pointing to significant cardiovascular risk of patients with persisting symptoms.

Research samples will be handled as per instructions in Sample Handling Manual. Samples will be archived at the NHS GGC Biorepository using a LIMS. Initial exploratory analysis will be at University of Glasgow,

Results of routine blood samples will be uploaded to NHS Clinical Portal / Trakcare systems for review by clinicians.

7.5.2 Sample analysis

Analysis of samples for vascular cytokines will be carried out in the Sattar/ Welsh laboratory, University of Glasgow.

The haemostasis and coagulation analyses will be undertaken in the NHS Haemostasis Laboratory (Dr Catherine Bagot), Department of Haematology, Glasgow Royal Infirmary. Immunology will be analysed in the NHS (Prof Rory Gunson) and/or University laboratories e.g. Prof Carl Goodyear, MRC CVR.

Sample collection, shipping, processing and analysis will be as per study sample manual and local SOPs.

7.5.3 Gluteal biopsy for peripheral vascular function and cell biology (ex vivo)

Patients in NHS Greater Glasgow and Clyde may take part in a vascular sub study involving a gluteal biopsy to be undertaken during convalescence. Participants who have passed the 3 month time point and who have not received information about the sub-study may be contacted to give them the opportunity to participate.

The patients who attend Visit 3 will be invited by the research staff. They will be given the information sheet at the time of the visit, or if this is omitted e.g. for logistical reasons, then the information sheet and consent form will be sent to their home address.

The biopsy will be undertaken according to an SOP in the clinical research facility.

The aim of the sub study is to study the vascular biology of the cells from isolated tissue which we will obtain by skin biopsy. Based on research undertaken in the University of Glasgow during the past two years, and by researchers in other institutions, COVID-19 may damage blood vessels. This may limit blood flow around the body and lead to feelings of tiredness and reduced exercise capacity. Our research aims to assess the nature of blood vessel problems with a view to identifying novel treatments that might be helpful to patients with persisting physical symptoms after COVID-19 e.g. Long COVID. The biopsy

tissue will be provided to researchers in the University of Glasgow or scientific collaborators in the UK or abroad with whom we are working

7.6 Oxygen saturation, respiratory rate and heart rate

Oxygen saturation, respiratory rate and heart rate should be measured at each visit, including at the start and end of the shuttle walk test.

7.7 Spirometry

Lung function should be assessed using spirometry at Visits 1 and Visit 2.
The basic measurement will be a peak expiratory flow rate (PEFR), forced vital capacity (FVC) and forced expiratory volume in one second (FEV1)

7.8 Muscle strength

Hand grip strength will be assessed as a measure of physical function at Visits 1 and 2. Handgrip strength will be recorded using a handheld dynamometer. For handgrip strength, measurements are performed until 3 measurements are within 5% of each other. Typically, 3 - 6 manoeuvres in each hand are performed. Responses in the dominant hand will be recorded.

7.9 Short Physical Performance Battery (SPPB)

The SPPB is a series of brief, simple physical tests including of the ability to stand for 10 seconds with the feet in 3 different positions (together side-by-side (score 0 – 1), semi-tandem (score 0 – 1), and tandem (score 0 – 2)), two timed trials of a 3m or 4m walk (fastest recorded) and the time to rise from a chair 5 times. The score ranges from 0 to 12 (higher scores reflect better extremity function). The gait and chair subtests score from 0 to 4.

SPPB will be supported by an SOP that takes account of information, training and standardisation.

7.10 Accelerometer

Accelerometery reflects body motion during daily activities. In a sub study focused to participants in Glasgow (Groups A and B), a portable accelerometer, styled as a watch, will be provided at baseline and again 3 months. An all-comers approach will be adopted with invitation to consecutively presenting patients. The device should be worn for 7 days lead.

GENEActiv accelerometer will be used and supported by a SOP with instructions and contact details if there are any issues. Of the available variables, the following are the parameters that will be obtained using the information collected via these devices:

- Total Physical activity
- Light Intensity Physical Activity
- Moderate Intensity Physical Activity
- Vigorous Intensity Physical Activity

- Sleep duration
- Sleep efficiency

7.11 Questionnaires

Patient reported outcome measures will be collected at each visit in order to assess their associations with the measurements (parameters) of cardiopulmonary injury / function. This would be with the intention to gain insights into the clinical significance of the research findings, and to assess temporal changes over time, assessed at baseline and at 3 months.

- EQ-5D: Health status and quality of life
- Anxiety/depression (PHQ4)
- Illness perception (Brief IPQ): Describes the participants self-reported health burden and perception
- Duke Activity Status Index (DASI): To assess the functional status (exercise capacity); this index is validated against the Bruce treadmill exercise test, and both are prognostically validated.
- International Physical Activity Questionnaire (IPAQ-SF): provides information on current / recent activity levels.
- Fatigue questionnaire including ISARIC fatigue visual analog score.
- MRC Dyspnoea score.
- DSQ Short Form PEM Questionnaire

7.12 Frailty

Frailty will be assessed using two scales, 1) the Fried frailty phenotype, five criteria: weight loss; exhaustion; grip strength; low physical activity; and slow walking pace (Appendix) and 2) the Clinical Frailty Scale. The Fried frailty phenotype includes distinct components whereas the Clinical Frailty Scale is a simple ordinal score that is more straightforward to complete. There is no frailty threshold for inclusion in the study, aside from the eligibility criterion that the patients should be able to walk.

7.13 Randomisation

Participants will be randomly assigned (1:1) to the intervention or control group. The randomisation sequence will be pre-determined before the start of the trial.

Participants who fulfil eligibility criteria and provide written informed consent will have randomisation stratified by group (clinical strata, Group A, B or C; see section 7.4, above).

The randomisation procedure will be undertaken by trained staff will use a web-based randomization tool hosted by the CTU.

7.14 Resistance exercise intervention

Intervention = a resistance-based exercise intervention for patients during the convalescence phase in-hospital through to 3-months post-discharge, a duration reflecting chronic, maintenance treatment studies. To maximise enrolment of eligible patients, the intervention may be initiated in-hospital or in the community post-discharge. Resistance bands may be used according to the exercise guideline.

Patients recovering from COVID-19 may spend many days in-hospital leading up to discharge. Patients will be placed within one of three broad categories of baseline physical capabilities (bed bound, up-to-sit, and ambulant) at the beginning of the intervention. Within the latter 2 categories, there will be progression of effort options and patients will be able to move between (up or down) these three categories.

The exercise regimens are generic and designed not to require expert physiotherapy input, therefore, the availability of NHS physiotherapists, which may be at times limited, will not be a barrier to implementation. If the study was successful it is hoped it could be adopted by ward staff. Progress will be reviewed and changes in the exercise plan will be adjusted according to a standard operating procedure. The participants may progress through the physical capability groups during the recovery phase from their illness.

Peripheral locomotor muscle training

Participants will be invited to train daily. Patients will be invited to perform the number of repetitions that leads to a validated resistance exercise specific Rating of Perceived Exertion (RPE) of 8-10 (PMID: 12569225). The use of RPE to prescribe and progress resistance exercise has been shown to be as efficacious as other more complex methods but reported to be the most tolerable and enjoyable method (PMID: 31107348). Once patients can reach 15-20 repetitions of each exercise they will be advised to move to the next level within each category and once able to do this for the top level within the category, to move to the next category. Similarly, if patients are not able to manage 5 repetitions of each exercise they will move down a level and, if at the bottom level within a category, down a category.

The overall exercise programme pack is provided a single document. The exercise programme has been developed by Dr Stuart Gray (Physiologist, University of Glasgow) with guidance from our PPI group (Dr Tracy Ibbotson, Prof Frances Mair, Primary Care), Drs Hannah Bayes and David Anderson (Rehabilitation, NHSGGC), Prof Rod Taylor (MRC Unit) and Clare Burke and David Boiskin (Physiotherapy, NHSGGC QEUH).

A training package will be provided to the site research staff (research nurses, fellows). The pack will be given to patients during an initial face to face session where the nurse/therapist will help to select the most suitable category and level of exercise for the patient, and to demonstrate these and ensure patients are comfortable performing the exercises. Every 2 weeks a member of the research team will contact the patients to ensure they are happy with the exercises and to help overcome any issues that have arisen. If the patient is in hospital, the contact may be daily, as needed.

Implementation at sites will be overseen and supported by a Working Group that includes these colleagues in an advisor role. The site research staff will be trained on the practical aspects of the exercises. Training will be given through in-person site visit.

Intervention group: An instructional pack (with video links and pictures) and an exercise log will be provided to empower each patient. We have put together a draft of the exercise programme pack for patients. This has been reviewed by our PPI group (4 Nov 2020) and feedback incorporated to reflect co-design of the programme. Based on our experience in the CISCO-19 study, we believe the exercise intervention will be feasible for most patients who have capacity to follow and adhere to verbal and/or written instructions. We aim for a widely generalisable intervention that can be taken up by most post-COVID-19 patients.

The programme is intended for patients in-hospital or at home and allowing for transition between the two. Our exercise intervention is supported by a systematic review of resistance training [18]. Specific protocols [15]: taking account of feedback to ensure simplicity, safety and remove barriers to implementation, household objects e.g. bottle of water, tin of food, which would be available to all participants, or exercise tension bands are also an option, according to patient preference. This demarcates our intervention from Physiotherapy and can be facilitated by other healthcare staff including nursing staff (hospital-based, community) and medical staff (including GPs).

LONG COVID-19 EXERCISE MANUAL (developed by Dr Stuart Gray, Prof Colin Berry and informed by feedback from the NHS GGC Patient Group, October – November 2020),

SUMMARY: Exercises are personalised options according to the circumstances of the individual patient: *Bed-bound, up-to-sit, ambulant.* The resistance exercise options are described in a manual.

Setting: Hospitalised patients, in their room on the ward and continued until post-discharge up to 3 months. Participants may be recruited post-discharge and would then come to hospital for consent and baseline assessment and thereafter would carry out exercises at home.

Timing and duration of interventions: Convalescence phase through to 3 months post-discharge. This time window is intended to follow-on from the acute therapy (and trials). The window focuses on the period when patients are recovering on the medical wards, through to 3 months post-discharge, which is when the first clinical assessment takes place in PHOSP (Tier 2). Our observations from the current landscape of clinical trials in the UK is that this time-period is not fully covered by the existing studies, hence our strategic focus for this enrolment window (post-ICU/HDU through to 3 months), which is a critical time for patients in their paths to recovery. Informed consent into our trial will complement co-enrolment into PHOSP (which is an observational study).

Patients who have been treated in ICU or HDU receive Physiotherapy as part of standard care, therefore, these patients would only be eligible for enrolment if the physiotherapy has completed. This reflects our plan for 'prehabilitation' without overlap with standard care NHS Physiotherapy. The focus for our trial(s) would mainly be during the post-ICU period to post-discharge e.g. 3 months (Tier 2, PHOSP), and options for longer term follow-up assessments, also aligned with PHOSP, will be feasible. Our aim is to synergise with PHOSP (aligning timing and endpoints), enhance enrolment and the compliance with follow-up.

7.15 Incremental shuttle walk test

Primary outcome: Incremental shuttle walk test (ISWT) [20]. This is a validated measure of functional capacity, with test-retest reliability and evidence of being responsive to rehabilitation interventions.

The ISWT is used as a Tier 2 evaluation in PHOSP. By adopting the ISWT as the primary outcome measure, our study will complement PHOSP, align with the protocol (for those patients who have been enrolled), and potentially, enhance co-enrolment into this observational study. Use of ISWT should be registered with the University of Leicester (Prof Sally Singh), including use of the digital timer for the test. Our staff have local experience since the PHOSP-COVID study is running in Dundee and Glasgow.

Oxygen saturation, heart rate and respiratory date at the start and end of the ISWT, and reflect exploratory outcome measurements.

7.16 Post-exercise malaise

Post-exercise issues such as fatigue, dizziness and soreness may occur. If post-exercise malaise arises participants can reduce the level of exercise, undertake the exercise less frequency or take a break until they feel ready to continue again. Post-exercise malaise (PEM) has been considered in the patient and public involvement (PPI) meetings and website information provided by Long Covid Support UK . Their feedback has been taken into account, including asking patients about whether they might experience post-exertional symptom exacerbation, to highlight the study will provide continued monitoring e.g. telephone contacts, and screen for problems with the autonomic nervous system, such as palpitations or light-headedness on standing. Participants will be invited to complete a post-exercise malaise questionnaire (DSQ Short Form PEM Questionnaire), prospectively and retrospectively, and their feedback can be discussed in telephone calls in order to improve the exercise activity for the patient in a personalised way.[23]

7.17 Data Retrieval and eCRF Input

A study clinician and/or delegated member of staff will be responsible for accessing the relevant patient data (A&E admission paperwork, discharge paperwork) and blood sample results on the NHSGG&C hospital systems, including Clinical Portal and/or Trakcare. The data provided on these systems will be considered as source data.

Patients will be allocated a unique patient number at the point of consent, and this is how they will be identified within the eCRF.

The eCRF will be developed and managed by the Robertson Centre for Biostatistics.

7.18 Record linkage

Patient contact is necessary for the study visits which will be minimised by telephone contact during the study period. At the other time-points, patient contact will not be necessary if access to electronic medical records is feasible and the data are clear. When patient contact is unsuccessful the GP may be contacted, and electronic health records will be checked including for the assessment of events, and medication use in the longer term. The follow-up will be by telephone, or by letter or clinic review as clinically appropriate.

In the longer term, additional follow-up of health outcomes, and related health information e.g. primary care attendances, pharmacy prescriptions, will be assessed by electronic case record linkage using the Community Health Index (CHI) number in Scotland.

Episodes in primary and secondary care, death, and causes in primary and secondary diagnostic positions, will be assessed using electronic health records held by the NHS and government / public sector agencies.

8. SAFETY REPORTING

8.1 Definitions

8.1.1 Adverse Event (AE)

Any untoward medical occurrence in a subject to whom a trial specific procedure has been administered, including occurrences which are not necessarily caused by or related to that trial specific procedure.

8.1.2 Serious Adverse Event (SAE)

Any adverse event or adverse reaction that:

a) Results in death
b) Is life threatening
c) Requires hospitalisation or prolongation of existing hospitalisation
d) Results in persistent or significant disability or incapacity
e) Consists of a congenital anomaly or birth defect
f) Is otherwise considered medically significant by the investigator
g) Important adverse events/reactions that are not immediately life-threatening or do not result in death or hospitalisation but may jeopardise the subject or may require intervention to prevent one of the other outcomes listed in the definition above.

8.1.3 Related Unexpected Serious Adverse Event (RUSAE)

Any SAE thought to be related to a trial specific procedure performed on that subject that is thought to be unexpected; that is the event is not listed within the protocol or would not be expected to occur when carrying out the trial specific procedure in normal clinical practise.

8.2 Serious adverse event recording and reporting

Where an SAE requires recording; full details including the nature of the event, start and stop dates, severity, relationship to research product and/or trial procedures, and the outcome of the event will be recorded in the patient's medical notes and CRFs. These events will be monitored and followed up until satisfactory resolution and stabilisation.

Each SAE should be assessed to determine if related to the research specific procedures and assessed for expectedness. Where an event meets the criteria of an SAE and is both:

- **Related:** that is, it resulted from any of the research procedures, **AND**

- **Unexpected**: that is against the procedure events listed below as an expected occurrence.

The SAE meets the criteria of a Related and Unexpected Serious Adverse Event (RUSAE) and is subject to expedited reporting to the Sponsor.

8.3 Expected Events Related to Phlebotomy Procedures

There is little risk to participants taking part in this study; however, there may be complications relating to the phlebotomy procedures such as:

- Haematoma
- Nerve injury
- Arterial puncture - If this occurs, remove the needle and apply direct pressure for at least 10 minutes; if a pulsatile mass persists, consider arterial duplex ultrasonography
- Syncope
- Phlebitis or cellulitis

These events would be considered expected within this study.

8.4 Expected Events related to resistance exercise

Complications of exercise intervention may be soft tissue strain, falls or exhaustion.

8.5 Reporting to the Sponsor

All RUSAEs must be reported to the Pharmacovigilance Office immediately (within 24 hours) using the generic non-CTIMP SAE form which is available from http://www.glasgowctu.org/data/SAE_non-CTIMP.pdf. The SAE form should be completed and signed by appropriately delegated staff. If necessary, a verbal report can be given by contacting the PV Office on 0141 330 4744. This must be followed up as soon as possible with an electronic report.
If all of the required information is not available at the time of initial reporting, the CI (or designee) must ensure that any missing information is forwarded to the PV Office as soon as this becomes available. The report should indicate that this information is follow-up information for a previously reported event.

8.6 Reporting of RUSAEs to the Ethics Committee

The PV office will report all RUSAEs to the ethics committee within 15 days of the PV office becoming aware of the event, via the 'report of serious adverse event form' for non-CTIMPs published on the Health Research Authority web site. http://www.hra.nhs.uk/documents/2015/02/safety-report-form-non-ctimp.docx. The form should be completed in typescript and signed by the Chief Investigator.

8.7 Pregnancy reporting (site responsibilities)

Pregnancy is an exclusion criterion. If after enrolment the participant becomes pregnant, this is not considered an AE or SAE in this trial unless a negative or consequential outcome is recorded for the mother or child/foetus. However, the patient would then be invited to withdraw from the study. If the outcome meets the serious criteria, this would be considered an SAE and must be reported as per SAE reporting procedure above.

8.8 Recording and reporting of SAEs where eCRF access is not possible

If recording in the eCRF is not possible a paper SAE form should be completed:

- The SAE form is downloaded from www.glasgowctu.org, printed off, completed and signed. The form is then faxed to the Glasgow Clinical Trials Unit Pharmacovigilance (PV) Office on +44(0)141 357 5588. If faxing is not possible a copy of the SAE form should be scanned and emailed to: pharmacovig@glasgowctu.org. If this website is unavailable a paper copy of the SAE form is filed in the Investigator Site File at each site.
- If necessary a verbal report can be given by contacting the PV Office on +44(0)141 330 4744. This must be followed up as soon as possible with an electronic or written report.

8.9 Responsibilities for Safety Reporting and Review

This section details the responsibilities for reporting and reviewing safety information arising from the trial.

8.10 Data Centre

The study Data Centre will:

- Provide an eCRF for central data collection of AEs and SAEs;
- Provide reports to the independent oversight committees identified for the trial (Independent Data Monitoring Committee (IDMC) and/or Trial Steering Committee (TSC)).

8.11 Principal Investigator (PI)

- Checking for SAEs when participants attend for treatment / follow-up.
- Ensuring that SAEs are recorded in line with the requirements of the protocol.
- Ensuring that all SAEs are recorded in the eCRF within 24 hours of becoming aware of the event and provide further follow-up information as soon as available.
- Using medical judgement in assigning seriousness, causality, severity and expectedness with reference to the trial protocol and Reference Safety Information.

8.12 Chief Investigator (CI)

- Clinical oversight of the safety of patients participating in the trial, including an ongoing review of the risk / benefit.
- Using medical judgement, confirm seriousness and causality and confirm expectedness of SAEs.
- Immediate review of life-threatening SAEs

8.13 Sponsor

- Verification of data collection, SAEs, according to the trial protocol
- Reporting safety information to the CI or delegate for the ongoing assessment of the risk / benefit
- Preparing in collaboration with the CI the annual safety report to the Research Ethics Committee and until the End of the Study, at which time an End of Study form will be submitted.

8.14 Trial Steering Committee

In accordance with the Charter for the TSC, periodically reviewing recruitment and the overall progress of the trial and liaising with the sponsor regarding safety issues.

9. Statistical Analysis

9.1 Statistical analysis plan

This study will have a comprehensive Statistical Analysis Plan, which will govern all statistical aspects of the study, and will be authored by the Trial Statistician before unblinded data are seen.

9.2 General considerations

The statistical Analysis Plan (SAP) will be based on intention to treat principles in line with CONSORT guidelines. The analysis will focus on estimation of treatment effect differences with 95% CIs and p-values. All pre-specified secondary outcome analyses will be reported in study publications.

9.3 Primary efficacy analysis

Alternative hypothesis: In patients with persisting symptoms during the convalescence phase after COVID-19, resistance exercise increases exercise capacity measured by the incremental shuttle walk test. The null hypothesis reflects no difference in exercise capacity between the groups.

9.3.1 Primary outcome

Incremental shuttle walk test at 3 months

9.3.2 Primary analysis and sample size

The minimum clinically important between-group difference in the ISWT at follow-up (3 months) = 46 m, SD=105 [20], sample size for 80% power, 5% significance, no loss to follow-up (LTFU) = a minimum of 85 / group; allowing for LTFU and incomplete data, the sample size is n=110/group (n=220 total).

9.4 Software and statistical analysis

Continuous outcomes will be analysed where data are approximately normally distributed and where baseline levels are available for adjustment. Where this is the case, two sample t-tests and corresponding confidence intervals will be used. Where data are clearly not normally distributed (e.g. laboratory variables) standard transformations will be applied to achieve approximate normality prior to analysis.

Clinical data will be made available to the data coordinating centre (the Robertson Centre for Biostatistics) through the web-based eCRF. The RCB is UKCRC-registered Clinical Trials Unit (number 16). The statistical software to be used will be SAS and/or R.

All analyses will be governed by a Statistical Analysis plan, to be finalised prior to database lock, and prior to sharing of any summary results. This plan will summarise the types of methods to be used for each study hypothesis, allowing some flexibility in the precise methods to be used, given the exploratory nature of the study.

9.5 Sample size calculation for the vascular function sub-study (myography)

Endpoint: Maximum contractile response (Emax, % relaxation of U46619, a thromboxane analog, or % contraction of KCl).

Cumulative concentration response curves (CCRCs) – Using preliminary vascular reactivity data from gluteal biopsies from post-COVID-19 vs. control subjects, we can assume a meaningful difference between the mean Emax values in the experimental and control groups as 23% (SD of 25). For a significance level of 0.05 and power of 80%, then a sample size of 10 per group is required. The calculations were done using Minitab®.

10. DATA HANDLING

10.1 Case Report Forms / Electronic Data Record

An electronic case report form (eCRF) will be used to collect study data. The eCRF will be developed by investigators in the University of Glasgow and access to the eCRF will be restricted, with only authorised site-specific personnel able to make entries or amendments to their patients' data. It is the investigator's responsibility to ensure completion and to review and approve all data captured in the eCRF.

All data handling procedures will be detailed in a Study Specific Data Management Plan. Data will be validated at the point of entry into the eCRF and at regular intervals during the study. Data discrepancies will be flagged to the study site and any data changes will be recorded in order to maintain a complete audit trail (reason for change, date change made, who made change).

The data management plan will inform sharing and access to bone fide researchers and a standard research agreement. Individuals/centres will be recognised through publications (acknowledgement and named authors in line with their contributions).

10.2 Data Retention

To enable evaluations and/or audits from regulatory authorities, the investigator agrees to keep records, including the identity of all participating subjects (sufficient information to link records), all original signed informed consent forms, serious adverse event forms, source documents, and detailed records of treatment disposition in accordance with EU GCP guidance, local regulations, or as specified in the Clinical Study Agreement, whichever is longer. Data will be retained at the Data Centre for a minimum of 10 years after the end of the study.

11. MONITORING, AUDIT & INSPECTION

NHSGGC conducts monitoring and/or audit of studies on a risk basis and in accordance with local Standard Operating Procedures. The level, frequency and priorities of monitoring and/or audit will be agreed with the NHS Governance Manager.

12. ETHICAL AND REGULATORY CONSIDERATIONS

12.1 Research Ethics Committee (REC) review & reports

Before the start of the trial, approval will be sought from a REC for the trial protocol, informed consent forms and other relevant documents e.g. advertisements and GP information letters.

Substantial amendments that require review by REC will not be implemented until the REC grants a favourable opinion (it is noted that amendments may also need to be reviewed and accepted by the NHS R&D departments before they can be implemented in practice at sites).

All correspondence with the REC will be retained in the Trial Master File/Investigator Site File.

An annual progress report (APR) will be submitted to the REC within 30 days of the anniversary date on which the favourable opinion was given, and annually until the trial is declared ended (this is the Chief Investigator's responsibility).

The Chief Investigator will notify the REC of the end of the study.

If the study is ended prematurely, the Chief Investigator will notify the REC, including the reasons for the premature termination.

Within 6 months after the end of the study, the Chief Investigator will submit a final report with the results, including any publications/abstracts, to the REC.

12.2 Peer review

The study design has benefitted from peer review through the Glasgow CTU, NHSGGC Patient and Public Involvement (PPI) group and the Chief Scientist Office of the Scottish Government (November – December 2020).

12.3 Public and Patient Involvement

Patient and public involvement (PPI) is led by Dr Tracy Ibbotson, of the Scottish Primary Care Research Network, who steers a patient group in NHS Glasgow. This group has been actively involved in the development of our proposal.

We will establish a PPI group of patients who have had lived experience of COVID-19. They will participate in PPI activities including the design of patient packs, linking to appropriate patient groups, and co-producing written reports for patient websites and conferences. Given the level of public interest, we anticipate sharing information about our study through the media e.g. BBC, newspapers with coordination by NHS GGC and University communications teams.

Our PPI group will meet every 3 months to oversee the study and funding is included in our budget. Constituting this group at the outset of the study will also be a deliverable and the reports (minutes) from each of the meetings (scheduled to correspond with the timelines/milestones).

PPI in our research will include a patient representative on the TSC.

12.4 Regulatory Compliance

The trial will not commence until full approval has been granted by the Research Ethics Committee, the R&D Management Office and Sponsor.

For any amendment that will potentially affect a site's NHS permission, the Chief Investigator/ Principal Investigator or designee will confirm with that site's R&D department that NHS permission is ongoing (note that both substantial amendments, and amendments considered to be non-substantial for the purposes of REC may still need to be notified to NHS R&D).

12.5 Protocol compliance

Prospective, planned deviations or waivers to the protocol are not allowed under the UK regulations on Clinical Trials and must not be used e.g. it is not acceptable to enrol a participant if they do not meet the eligibility criteria or restrictions specified in the trial protocol. Accidental protocol deviations can happen at any time. They must be adequately documented on the relevant forms and reported to the Chief Investigator, Sponsor and GCTU immediately. Deviations from the protocol which are found to frequently recur are not acceptable, will require immediate action and could potentially be classified as a serious breach.

12.6 Notification of Serious Breaches to GCP and/or the protocol

A "serious breach" is a breach which is likely to affect to a significant degree –

- the safety or physical or mental integrity of the subjects of the trial; or
- the scientific value of the trial

If any of the above occurs, then the CI and Sponsor will be notified. The sponsor will notify the appropriate authorities in writing of any serious breach in accordance with their standard operating procedures.

12.7 Data protection and patient confidentiality

All investigators and trial site staff must comply with the requirements of applicable data protection legislation with regards to the collection, storage, processing and disclosure of personal information and will uphold the core principles of such legislation.

Personal information will be collected via the eCRF to enable record linkage to be carried out and to provide electronic access to study monitors to a copy of the signed informed consent document. These data items will be encrypted and only those individuals who require to see these data i.e. the person performing the record linkage and site research team staff or the study monitor, as appropriate, will be able to view them. All electronic data will be held securely in accordance with ISO 27001:2013 at the Robertson Centre for Biostatistics, part of the Glasgow Clinical Trials Unit. All Centre staff are required to sign confidentiality agreements and to follow Standard Operating Procedures in accordance with Good Clinical Practice and ISO certification.

The trial data managers, statisticians, or any other staff who will perform data related tasks will only be able to access depersonalised data where the participant's identifying information is replaced by a unique study identifier.

Only those that have been trained and approved will be able to enter or view any data via the web portal. Each site can only see their own patients' data. Patient consent forms will be stored at the study site in a secure location accessible only to study teams.

12.8 Financial and other competing interests for the chief investigator, PIs at each site and committee members for the overall trial management

A log of financial or other competing interests for the CI, PIs and committee members will be held centrally by the Trial Coordinator throughout the trial. The Trial Coordinator will request this information at the site initiation visit and at regular intervals during study conduct, and it will be made available to the Sponsor.

12.9 Indemnity

The NHS Indemnity Scheme will apply to this study to meet the potential legal liability of the sponsor for harm to participants arising from the management of the research.

The University of Glasgow Clinical Trials Insurance will apply to this study to meet the potential legal liability for harm to participants arising from the design of the research.

The sponsor has not made any arrangements for payment of compensation in the event of harm to the research participants where no legal liability arises. University of Glasgow insurance is also relevant.

12.10 Amendments

Any change in the study protocol will require an amendment. Any proposed substantial protocol amendments will be initiated by the CI following discussion with the Sponsor and TSC and any required amendment forms will be submitted to the, ethics committee and Sponsor. The Sponsor will determine whether an amendment is non-substantial or substantial. All amended versions of the protocol will be signed by the CI and Sponsor representative. Following a substantial amendment, favourable opinion/approval must be sought from the original reviewing REC, and Research and Development (R&D) office prior to implementation. The Chief Investigator will be responsible for informing the Trial Management Group of all protocol amendments.

12.11 Post-trial care

At the end of the trial, participants will be returned to usual care as defined by local and national guidelines at that time.

12.13 Access to the final trial dataset

During the trial and in the period prior to publication of the main study results as described in the protocol, only the Glasgow CTU will have access to the full dataset. After that period, the Trial Steering Committee will conduct further data analyses for a period of three years. After that time the Trial Steering Committee will consider requests from external parties for further analyses of the study data. The TSC will review the proposals and provide a view on scientific merit to the Sponsor and CI. Proposals that are scientifically well founded and have an academic basis and where relevant

data extractions and analyses are appropriately funded will not be refused. These will be considered as collaborative exercises where the contributions related to study design, conduct, database creation and maintenance and data analysis will be recognised in authorship of any scientific publication. The approach we will take will be to minimise any possibility of breach of participant confidentiality. Normally this will be achieved by minimising data travel. However, for the purposes of individual patient meta-analysis and other reasons, data may be transferred to other sites. Such transfer will require assurances on information security systems at the sites that data are to be transferred to and will involve a legal data transfer agreement. A log of all data requests and subsequent data transfers will be held at the Glasgow CTU.

12.

13. DISSEMINATION POLICY

13.1 Dissemination policy

The data arising from this study will be owned by the University of Glasgow and NHS Greater Glasgow and Clyde.

Once the study has been completed, a final report will be prepared for publishing purposes, and to feedback research results to both sponsor and REC. This will be provided to sponsor/REC via email and will be made accessible to the wider research community on international study registry websites such as clinicaltrials.gov. The CI will have the right to publish the study data. There are no plans to notify participants of the outcome of the study. Participants will be provided with CI/PI contact details in the Patient Information Sheet. Participants will be advised upon requesting results that these will be made available once data analysis has been completed and/or the final study report has been compiled.

The main study documentation - study protocol and full study report – will be made accessible to the wider research community on international study registry websites such as clinicaltrials.gov within 1 year of study opening.

13.2 Authorship eligibility guidelines and any intended use of professional writers

Key contributors to the protocol and final report will be noted as authors, as will study clinicians who contribute significantly to the running/management of the study at site. All will be provided with a draft copy of the final report for review prior to publication. Criteria for individually named authors or group authorship will adhere to that of The International Committee of Medical Journal Editors.

14. REFERENCES

1. Coronavirus (COVID-19): daily data for Scotland. Scottish Government. https://www.gov.scot/publications/coronavirus-covid-19-daily-data-for-scotland/
2. Ball S, Banerjee A, **Berry C,** et al; CVD-COVID-UK Consortium. Monitoring indirect impact of COVID-19 pandemic on services for CV diseases in the UK. Heart. 2020 Oct 5:heartjnl-2020-317870.
3. Docherty AB, Harrison EM, Green CA, et al. Features of 20 133 UK patients in hospital with covid-19 using the ISARIC WHO Clinical Characterisation Protocol: prospective observational cohort study. BMJ British Medical Journal Publishing Group; 2020;369.
4. **Mangion K,** Morrow A, Bagot C, …**Berry C**. The Chief Scientist Office Cardiovascular and Pulmonary Imaging in SARS Coronavirus disease-19 (CISCO-19) study. Cardiovasc Res. 2020 Jul 23:cvaa209. doi: 10.1093/cvr/cvaa209. Epub ahead of print. PMID: 32702087; PMCID: PMC7454350.
5. **Sattar N,** Ho FK, Gill JM, .., **Berry C**, Pell JP, McMurray JJ, Welsh P. BMI and future risk for COVID-19 infection and death across sex, age and ethnicity: Preliminary findings from UK biobank. Diabetes Metab Syndr. 2020 Sep-Oct;14(5):1149-1151.
6. Guzik TJ, .. **Berry C,** Touyz RM, Kreutz R, Wang DW, Bhella D, Sagliocco O, Crea F, Thomson EC, McInnes IB. COVID-19 and the cardiovascular system: implications for risk assessment, diagnosis, and treatment options. Cardiovasc Res. 2020 Aug 1;116(10):1666-1687.
7. Libby P, Lüscher T. COVID-19 is, in the end, an endothelial disease. Eur Heart J. 2020 Sep 1;41(32):3038-3044. doi: 10.1093/eurheartj/ehaa623. PMID: 32882706; PMCID: PMC7470753.
8. Dweck MR, Bularga A, Hahn RT, et al. Global evaluation of echocardiography in patients with COVID-19. Eur Heart J Cardiovasc Imaging Oxford Academic; 2020;21:949–958.
9. Gualtieri P, Falcone C, Romano L, Macheda S, Correale P, Arciello P, Polimeni N, Lorenzo A. Body Composition Findings by Computed Tomography in SARS-CoV-2 Patients: Increased Risk of Muscle Wasting in Obesity. Int J Mol Sci. 2020 Jun 30;21(13):4670.
10. Morley JE, Kalantar-Zadeh K, Anker SD. COVID-19: a major cause of cachexia and sarcopenia? J Cachexia Sarcopenia Muscle. 2020 Aug;11(4):863-865.
11. Berry C, Clark AL. Catabolism in chronic heart failure. Eur Heart J. 2000 Apr;21(7):521-32. 12. Breen L, Stokes KA, Churchward-Venne TA, et al. Two weeks of reduced activity decreases leg lean mass and induces "anabolic resistance" of myofibrillar protein synthesis in healthy elderly. J Clin Endocrinol Metab. 2013 Jun;98(6):2604-12.
13. Krogh-Madsen R, Thyfault JP, Broholm C, et al. A 2-wk reduction of ambulatory activity attenuates peripheral insulin sensitivity. J Appl Physiol (1985). 2010 May;108(5):1034-40.
14. Rooney S, Webster A, Paul L. Systematic Review of Changes and Recovery in Physical Function and Fitness After Severe Acute Respiratory Syndrome-Related Coronavirus Infection: Implications for COVID-19 Rehabilitation. Phys Ther. 2020 Sep 28;100(10):1717-1729.
15. Al Ozairi E, Alsaeed D, Taliping D, .. **Sattar N**, Gray C, Welsh P, **Gray SR**. Protocol for a randomised controlled trial to investigate the effect of home- and gym-based resistance exercise training on glycaemic control, body composition and muscle strength. Trials. 2020 Jul 15;21(1):650.
16. Long Covid: https://www.bhf.org.uk/informationsupport/heart-matters-magazine/news/coronavirus-and-your-health/long-covid-the-long-term-symptoms-of-coronavirus (21 October 2020)
17. Sudre CH, Murray B, Varsavsky T et al. Attributes and predictors of Long-COVID: analysis of COVID cases and their symptoms collected by the Covid Symptoms Study App. https://www.medrxiv.org/content/10.1101/2020.10.19.20214494v1

18. Hart PD, Buck DJ. The effect of resistance training on health-related quality of life in older adults: Systematic review and meta-analysis. Health Promot Perspect. 2019 Jan 23;9(1):1-12.

19. Singh SJ, Morgan MD, Scott S, Walters D, Hardman AE. Development of a shuttle walking test of disability in patients with chronic airways obstruction. Thorax. 1992 Dec;47(12):1019-24.

20. Puente-Maestu L, et al. Use of exercise testing in the evaluation of interventional efficacy: an official ERS statement. Eur Respir J. 2016 Feb;47(2):429-60.

21. Ayoubkhani D et al. Epidemiology of post-COVID syndrome following hospitalisation with coronavirus: a retrospective cohort study. https://doi.org/10.1101/2021.01.15.21249885

22. The OpenSAFELY Collaborative; John Tazare et al. Rates of serious clinical outcomes in survivors of hospitalisation with COVID-19: a descriptive cohort study within the OpenSAFELY platform. https://doi.org/10.1101/2021.01.22.21250304

23. Clinical management of COVID-19: Living guideline, 15 September 2022 https://www.who.int/publications-detail-redirect/WHO-2019-nCoV-Clinical-2022.2

15. APPENDICES

15.1 Appendix 1 – Amendment history

Amendment No.	Protocol version no.	Date issued	Author(s) of changes	Details of changes made
01	2.0	17.03.22	Colin Berry	Addition of gluteal biopsy as a sub study.

List details of all protocol amendments here whenever a new version of the protocol is produced.

Protocol amendments must be submitted to the Sponsor for approval prior to submission to the REC committee.

15.2 Appendix 2. Demographic, medical and COVID-19 history

Participant details

**CHI – Place Label**	Age …… Years Sex: Male / Female<br>Height _________ Weight _________
Ethnicity	☐Arab ☐Black ☐East Asian ☐South Asian ☐ West Asian ☐ Latin American ☐ White
Date of admission	….. / ….. /
COVID-19 symptom onset date	… /…/
Date of COVID-19 diagnosis	… /…/
Blood pressure at presentation	Systolic / Diastolic (mmHg)
Heart rate at presentation	bpm
Initial troponin, ng/L	
Peak Troponin I, ng/L	
Date of peak troponin I	….. / ….. /
Initial point of care	A&E / AMRU / CCU / Medicine / Care of Elderly / Surgery / ICU
Second point of care	AMRU / CCU / Cardiology / Medicine / Care of Elderly / Surgery / ICU
Third point of care	AMRU / Cardiology / Medicine / Care of Elderly / Surgery / ICU
Date of discharge	….. / ….. /

Co-morbidity

Charlson Comorbidity Index (CCI)

Predicts 10-year survival in patients with multiple comorbidities.

Age

<50 years
0
50–59 years
+1
60–69 years
+2
70–79 years
+3
≥80 years
+4
Myocardial infarction
History of definite or probable MI (ECG changes and/or enzyme changes)
No
0
Yes
+1
CHF
Exertional or paroxysmal nocturnal dyspnoea and has responded to digitalis, diuretics, or afterload reducing agents
No
0
Yes
+1
Peripheral vascular disease
Intermittent claudication or past bypass for chronic arterial insufficiency, history of gangrene or acute arterial insufficiency, or untreated thoracic or abdominal aneurysm (≥6 cm)
No
0
Yes
+1
CVA or TIA
History of a cerebrovascular accident with minor or no residual and transient ischemic attacks
No
0
Yes
+1
Dementia
Chronic cognitive deficit
No
0
Yes
+1
COPD
No
0
Yes
+1
Connective tissue disease
No
0
Yes

+1

Peptic ulcer disease

Any history of treatment for ulcer disease or history of ulcer bleeding

No
0

Yes
+1

Liver disease

Severe = cirrhosis and portal hypertension with variceal bleeding history, moderate = cirrhosis and portal hypertension but no variceal bleeding history, mild = chronic hepatitis (or cirrhosis without portal hypertension)

None
0

Mild
+1

Moderate to severe
+3

Diabetes mellitus

None or diet-controlled
0

Uncomplicated
+1

End-organ damage
+2

Hemiplegia

No
0

Yes
+2

Moderate to severe CKD

Severe = on dialysis, status post kidney transplant, uraemia, moderate = creatinine >3 mg/dL (0.27 mmol/L)

No
0

Yes
+2

Solid tumour

None
0

Localized
+2

Metastatic
+6

Leukaemia

No
0

Yes
+2

Lymphoma

No
0

Yes
+2

AIDS

No
0

Yes
+6

Is this a COVID-19 patient?
For research purposes only; answer does NOT impact results.

Confirmed positive
Suspected
Unlikely
Confirmed negative

Outcome

= CCI index (points)

= % estimated 10-year survival

**COVID Risk score**	
ISARIC-4<br>WHO	

**Cardiovascular symptoms**	
Angina	Canadian Cardiovascular Society angina class: 1 / 2 / 3 / 4
	If angina is reported, then subject to complete angina diary
Function s	New York Heart Association Functional Class 1 / 2 / 3 / 4

**CV Risk scores**	
Heart age score	https://www.bhf.org.uk/informationsupport/risk-factors/check-your-heart-age
QRisk3 Risk score	https://www.qrisk.org/three/index.php

Smoking status, defined as per the internationally established definitions https://www.cdc.gov/nchs/nhis/tobacco/tobacco_glossary.htm

Current smoker:	An adult who has smoked 100 cigarettes in his or her lifetime and who currently smokes cigarettes. Beginning in 1991 this group was divided into "every day" smokers or "some days" smokers.

Never smoker:	An adult who has never smoked, or who has smoked less than 100 cigarettes in his or her lifetime.
Former smoker:	An adult who has smoked at least 100 cigarettes in his or her lifetime but who had quit smoking at the time of interview
Some days smoker:	An adult who has smoked at least 100 cigarettes in his or her lifetime, who smokes now, but does not smoke every day. Previously called an "occasional smoker".
Every day smoker:	An adult who has smoked at least 100 cigarettes in his or her lifetime, and who now smokes every day. Previously called a "regular smoker".

Pre-existing preventive cardiovascular therapy (medication prescribed prior to initial admission)

Aspirin / Clopidogrel / Ticagrelor	ACE-I or ARB
Statin	Mineralocorticoid (spironolactone or eplerenone)
Beta-blocker	
Anticoagulation = warfarin or NOAC (circle)	

Discharge preventive therapy (final discharge medication)

Aspirin / Clopidogrel / Ticagrelor	ACE-I or ARB
Statin	Mineralocorticoid (spironolactone or eplerenone)
Beta-blocker	
Anticoagulation = warfarin or NOAC (circle)	

COVID-19

COVID-19 diagnosis		
Clinical diagnosis	Yes	No
Laboratory test - positive PCR test	Yes	No
Laboratory test – antibody test	Yes	No
Radiology diagnosis - CXR	Yes	No
CXR findings		
Radiology diagnosis – CT scan	Yes	No
CT findings		
C-19 = main/primary cause of admission to hospital i.e. suspected/known on admission<br>(community acquired)	Yes	No
C-19 = developed in hospital (nosocomial)		
Admission diagnosis = non-COVID; i.e. COVID-19 diagnosed occurred in-hospital	Yes	No
Healthcare worker	Yes	No
ICU	Yes	No
COVID-19 treatment		
Supplemental oxygen		
Non-invasive respiratory support e.g. BIPAP, CPAP		
Invasive ventilation		
Extracorporeal membrane oxygenation		
Inotropes		
Haemodialysis		
COVID-19 Research Study: TITLE = ………………………………………………..		
Control – no treatment or placebo		
Antiviral – e.g. Remdesivir		
Antiviral (other) Lopinavir 400mg-Ritonavir 100mg or		
Immune regulator e.g. tocilizumab, sarilumab		
Steroid e.g. Dexamethasone		
Colchicine		

Aspirin		
Plasma		
Blinded study drug		
Other – please specify		

Myocarditis Diagnostic Criteria

Caforio et al. Position statement of the ESC Working Group on Myocardial and Pericardial Diseases, European Heart Journal (2013) 34, 2636–2648 [68].

**Clinical presentations (A) (TICK if present)**	
	Acute chest pain,<br>If yes circle / mark - pericarditic / pseudo-ischaemic (myocarditis) / ischaemic / dyspepsia / pleuritic.
	New-onset (days up to 3 months) or worsening of: dyspnoea at rest or exercise, and/or fatigue, with or without left and/or right heart failure signs
	Subacute/chronic (.3 months) or worsening of: dyspnoea at rest or exercise, and/or fatigue, with or without left and/or right heart failure signs
	Palpitation, and/or unexplained arrhythmia symptoms and/or syncope, and/or aborted sudden cardiac death<br>Unexplained cardiogenic shock
	= TOTAL NUMBER PRESENT
**Diagnostic criteria (TICK if present)**	
	I. ECG/Holter/stress test features - Newly abnormal 12 lead ECG and/or Holter and/or stress testing, any of the following: I to III degree atrioventricular block, or bundle branch block, ST/T wave change (ST elevation or non ST elevation, T wave inversion), sinus arrest, ventricular tachycardia or fibrillation and asystole, atrial fibrillation, reduced R wave height, intraventricular conduction delay (widened QRS complex), abnormal Q waves, low voltage, frequent premature beats, supraventricular tachycardia
	II. Myocardiocytolysis markers – elevated troponin
	III. Functional and structural abnormalities on cardiac imaging (echo/angio/CMR) - New, otherwise unexplained LV and/or RV structure and function abnormality (including incidental finding in apparently asymptomatic subjects): regional wall motion or global systolic or diastolic function abnormality, with or without ventricular dilatation, with or without increased wall thickness, with or without pericardial effusion, with or without endocavity thrombi

	IV. Tissue characterization by CMR – Modified Lake Louise Criteria (T2 map abnormali T1 abnormality (native T1 map, late gadolinium enhancement, extracellular volume (ECV)
	= TOTAL NUMBER PRESENT

Diagnostic criteria

Clinically suspected myocarditis if ≥1 clinical presentation and ≥1 diagnostic criteria from different categories, in the absence of: (1) angiographically detectable coronary artery disease (coronary stenosis ≥ 50%), if known; (2) known pre-existing cardiovascular disease or extra-cardiac causes that could explain the syndrome (e.g. valve disease, congenital heart disease, hyperthyroidism, etc.) Suspicion is higher with higher number of fulfilled criteria.

A - If the patient is asymptomatic ≥2 diagnostic criteria should be met.

Myocarditis should be suspected in the presence of:

1 or more of the clinical presentations, with or without ancillary features (see below), and
1 or more of the diagnostic criteria from different categories (I to IV) in Table 4 or when the patient is asymptomatic, 2 or more diagnostic criteria from different categories (I to IV).

To completed by clinician:

ACS (at any time)

Myocardial injury is defined as a high sensitivity troponin I > upper reference limit (99th centile).

Myocardial injury	Yes / No
Myocardial injury	Acute / chronic
ACS	Yes / No
Initial primary diagnosis	Type 1 MI / Type 2 MI / Type 3 MI / Myocardial Injury (Cardiac / Non-cardiac)*
Initial secondary diagnosis	Type 1 MI / Type 2 MI / Type 3 MI / Myocardial Injury (Cardiac / Non-cardiac)
Final primary diagnosis	Type 1 MI / Type 2 MI / Type 3 MI / Myocardial Injury (Cardiac / Non-cardiac)
Final secondary diagnosis	Type 1 MI / Type 2 MI / Type 3 MI / Myocardial Injury (Cardiac / Non-cardiac)

*For T2MI / myocardial injury, circle either cardiac or non-cardiac

T2MI / Myocardial Injury - Inciting cause or associated (tick all/multiple)

CARDIAC	NON-CARDIAC

Tachyarrhythmia	Anaemia
Bradyarrhythmia	Hypoxia
Coronary embolus/spasm/dissection/microvascular	Renal disease
Heart failure / cardiomyopathy	Surgery (recent)
Hypotension	Pulmonary embolism
Hypertension	Infection – COVID-19
Structural heart disease e.g. valve	Infection – non-COVID: - Chest, Abdomen,<br>UTI, Cellulitis, CNS, other - specify
Infection – endocarditis, myocarditis, pericarditis	COPD
Stress	
Other – specify	

Docusign Envelope ID: AF5020A8-A0EF-446D-839B-4F824F00D5A0

Glasgow Clinical Trials Unit Form

Form number	**10.003A**	Version	**1.0**
Title	**Statistical Analysis Plan (SAP)**		

Prevention and early treatment of the long term physical effects of COVID-19: a randomised clinical trial of resistance exercise. CISCO-21

STATISTICAL ANALYSIS PLAN (SAP)

Study Title:	Prevention and early treatment of the long term physical effects of COVID-19: a randomised clinical trial of resistance exercise.
Short Title:	CISCO-21
IDs:	Sponsor ID: GN20CA537<br>Funder ID: COV/LTE/20/10<br>IRAS number: 294299<br>ISRCTN number: NCT04900961
Sponsor:	NHS Greater Glasgow & Clyde
Funded by:	Chief Scientist Office
Protocol Version:	6.0
SAP Version Number:	1.0
Date:	

		Signature	Date
Prepared by:	Gemma McKinley	DocuSigned by: [signature] D906DFED071F4D5...	07 November 2024
	Junior Biostatistician, Robertson Centre for Biostatistics		
Approved by:	Alex McConnachie	DocuSigned by: [signature] 3463596312F04EA...	07 November 2024
	Director of Biostatistics, Robertson Centre for Biostatistics		
	Colin Berry	[signature]	6 November 2024
	Honorary Consultant Cardiologist, NHS Golden Jubilee Hospital		

Docusign Envelope ID: AF5020A8-A0EF-446D-839B-4F824F00D5A0

CONTENTS

1. Introduction 3
   1.1. Study Background 3
   1.2. Study Objectives 3
   1.3. Study Design 3
   1.4. Randomisation 3
   1.5. Sample Size and Power 4
   1.6. Statistical Analysis Plan (SAP) 4
2. Analysis 5
   2.1. Study Populations 5
   2.2. Study Status 6
   2.3. Baseline Characteristics 6
   2.4. Efficacy Outcomes 7
   2.5. Safety Outcomes 9
3. Data Conventions 10
4. Tables and Figures 10
5. Document History 10

1. Introduction

1.1. Study Background

Many patients who have had COVID-19 describe persisting health problems after their infection. Long COVID has been defined as signs and symptoms which develop during or following a COVID-19 infection, which continue for more than 12 weeks and are not explained by an alternative diagnosis. Symptoms in long COVID patients include breathlessness, fatigue, and chest pain. COVID-19 infection, particularly where hospital admission is required, can lead to weight loss and muscle wasting, which can contribute to worse outcomes. Muscle strengthening (resistance-based) exercise could improve outcomes in the long term. Muscle mass is a strong predictor of prognosis in many chronic conditions, the main method to improve muscle mass is through resistance exercise. This approach can be adapted to the capability of an individual with little equipment required. There is a general lack of evidence-based medicines for patients with persisting symptoms following COVID-19. The CISCO-21 study aims to develop and test a lifestyle intervention which could be helpful to patients with persisting symptoms following COVID-19 infection.

1.2. Study Objectives

The objectives of the study are to:

- Undertake a clinical trial of pragmatic resistance-based exercise in addition to standard care versus standard care only to prevent and treat persisting symptoms following COVID-19 infection.

- Rapidly translate and disseminate research findings.

- Develop a national platform for rapid, multicentre clinical trials post-COVID-19.

The research questions are:

- Does resistance-based exercise intervention improve functional capacity and health status post-COVID-19?

- Do the results of this trial elucidate mechanisms of disease in Long COVID?

- Is a national clinical trials platform for post-COVID patients in NHS Scotland feasible?

1.3. Study Design

A randomised, controlled, open-label, parallel group clinical trial.

1.4. Randomisation

Participants are recruited from three sites: Queen Elizabeth University Hospital (QEUH), Glasgow Royal Infirmary (GRI), and Ninewells Hospital. Patients who met the eligibility criteria and provided written informed consent were randomised to either standard care or

Docusign Envelope ID: AF5020A8-A0EF-446D-839B-4F824F00D5A0

resistance-based exercise in addition to standard care. Randomisation was stratified by clinical strata groups A, B, and C (see study population), history of COVID-19 pneumonia, age, sex, and site.

1.5. Sample Size and Power

The proposed sample size for the main study was 240 randomised participants, with 120 in each randomised group. The minimum clinically important between-group difference in the ISWT at 3 months is 46 metres, standard deviation 105. The sample size for 80% power at a 5% significance level with no loss to follow-up would be a minimum of 85 participants in each randomised group. The proposed sample size of 120 patients per group allows for loss to follow-up and incomplete data.

1.6. Statistical Analysis Plan (SAP)

1.6.1. SAP Objectives

The objective of this SAP is to describe the analyses to be carried out for the CISCO-21 final analysis.

The current version of the protocol at the time of writing is version 6.0 dated 11/01/2024. Future amendments to the protocol will be reviewed for their impact on this SAP, which will be updated only if necessary. This will be documented as part of the Robertson Centre Impact Assessment process.

1.6.2. General Principles

This SAP is based on intention to treat principles. For tables summarising collected data, categorical variables will be presented with a count of observed and missing observations, and a count and percentage for each category. Numerical data will be presented with a count of observed and missing observations, as well as mean, standard deviation, median, Q1, Q3, and minimum and maximum values (unless stated otherwise). The analysis will focus on estimations of treatment effect differences with 95% confidence intervals and p-values. Where data are not Normally distributed, standard transformations will be applied to achieve approximate Normality in the model residuals.

1.6.3. Additional Analyses To Those Specified In Study Protocol

There are currently no plans to carry out any additional analyses. If any additional analyses are to be performed, a log of these will be kept with details of the programs written and results documents produced.

1.6.4. Protocol Deviations

This SAP has been written to be consistent with the protocol. A listing with details of protocol violators will be made available within the study database prior to lock.

Docusign Envelope ID: AF5020A8-A0EF-446D-839B-4F824F00D5A0

1.6.5. Software

Analyses will be conducted using R version 4.2.3 or higher, with the version of R used included in statistical output documents.

2. Analysis

2.1. Study Populations

Potential study participants are patients with confirmed COVID-19 who are either recovering in hospital, have been discharged from hospital, or not admitted to hospital, and who have persisting symptoms for at least 4 weeks. Patients are classified according to their clinical presentation into one of three groups:

A) Non-hospitalised (treatment group): Positive diagnosis with persisting symptoms for at least 4 weeks from onset of symptoms leading to medical review (e.g. at A&E or a community COVID hub), but not admission.

B) Hospitalised (treatment group): Positive diagnosis with post-discharge, persistent symptoms for at least 4 weeks following symptom onset.

C) Hospitalised (prevention group): Positive diagnosis, in convalescent phase in hospital.

Groups A and B are the target population for treatment of Long COVID, while group C is the target population for prevention of Long COVID.

The inclusion criteria are:

- At least one of the following:
    - Virology PCR positive laboratory diagnosis, or positive point of care test for COVID-19
    - Positive lateral flow test (confirmation from notes or participant)
    - Positive COVID antibody test.
- Persistent symptoms for at least four weeks from onset of symptoms (group A and B only).
- Presentation type consistent with one of group A, B, or C.

The exclusion criteria are:

- Inpatient physiotherapy as part of current standard care post-ICU.
- No expectation of being able to walk within 3 months.

- Unable to provide informed consent.
- Unable to comply with study protocol.
- Known pregnancy.

Descriptive analyses of the study population will be provided, including the number of patients screened and randomised, summaries of inclusion/exclusion criteria, as well as details of why participants were not randomised. The screened population is all participants who attended the screening visit as reported in the screening log, while the randomised population is all participants who went on to participate in the trial. The full analysis set will consist of the randomised population who attended both study visits A CONSORT diagram will be created to illustrate progression of the participants through the study.

2.2. Study Status

At the time of writing, there are 233 participants randomised, and no further participants will be recruited or randomised. Follow-up is ongoing. Study status will be presented in tables with numbers who completed the study, withdrew, and were lost to follow-up. Counts for how many patients attended each visit will also be presented.

2.3. Baseline Characteristics

The following information will be summarised as a whole and in relation to randomised group for participants at baseline:

- Demographics (including age, sex, ethnicity, healthcare worker status, ICU worker status, and smoking status)
- Clinical presentation (group A, B, or C, as described in Study Population)
- COVID-19 details (including details of index COVID episode care (diagnosis and treatment), Index Illness severity scores (ISARIC 4c COVID Risk Score, WHO Clinical Severity Score), post-COVID lung disease, ACS associated with COVID-19, vaccination status, and re-infection)
- Medical history (cardiovascular and respiratory), and clinical status;
- Pre-existing CV therapy
- Charlson Comorbidity index
- MRC Dyspnoea Score
- Vital signs (including heart rate, rhythm, BP, height, weight, waist circumference, and oxygen saturation);
- Cardiovascular risk factors and risk score (QRISK3, Heart Age Score);

Docusign Envelope ID: AF5020A8-A0EF-446D-839B-4F824F00D5A0

- Routine blood samples (as per standard of care);
- Spirometry (peak expiratory flow rate, forced vital capacity, and forced expiratory volume in one second);
- Handgrip strength (peak force in kilograms);
- Short Physical Performance Battery;
- Accelerometer (Glasgow sites only, QUEH and GRI);
- Patient recorded outcome measures (including EuroQol-5D, brief Illness Perception Questionnaire, Duke Activity Status Index, short form International Physical Activity Questionnaire, Fatigue Severity Scale, Visual Analog Fatigue Scale, and DePaul Symptom Questionnaire);
- Frailty measures (Fried 5 criteria phenotype and the clinical frailty scale);
- Incremental Shuttle Walk Test;
- Current exercise capacity (Exercise Dose category - bedbound, up-to-sit, ambulatory).

For a supplementary document these characteristics may also be summarised according to the clinical presentation group of participants.

2.4. Efficacy Outcomes

2.4.1. Primary Outcome

The primary outcome is the incremental shuttle walk test (ISWT) at 3 months. This is a validated measure of functional capacity, with evidence of being responsive to rehabilitation interventions. This will be summarised overall and by randomised treatment group at baseline, 3 months, and the change between these two time points. The outcome will be analysed using a linear regression model which will be adjusted for baseline value of ISWT, the randomised treatment group, clinical strata groups A, B, and C (see study population), history of COVID-19 pneumonia, age, and sex. If the outcome is non-Normally distributed, standard transformations will be used to improve model fit. To check the fit of this model, a Normal Q-Q plot will be produced and inspected, and covariates will be assessed for collinearity.

2.4.2. Secondary Outcomes

1) The secondary outcomes are listed below. These will be summarised in the overall study population and randomised treatment groups. Spirometry (measurements taken are listed in Baseline Characteristics).
2) Handgrip strength.

3) Short Physical Performance Battery (SPPB).

4) EQ-5D, Patient Health Questionnaire (PHQ), illness perception (brief IPQ), Duke Activity Status Index (DASI), International Physical Activity Questionnaire (IPAQ-SF), fatigue questionnaires, and MRC dyspnoea score.

5) Frailty, as assessed by the Fried frailty phenotype (five criteria – weight loss, exhaustion, grip strength, low physical activity, and slow walking pace) and the Clinical Frailty Scale (both current frailty and the best frailty within the previous month).

6) Episodes of care (primary, secondary, physiotherapy, rehabilitation). These events will be summarised but analysis of this outcome is not covered by this SAP.

7) Hospitalisation for any reason. Hospitalisation events will be summarised but analysis of this outcome will be documented separately.

Where continuous data are approximately Normally distributed, and baseline levels are available for adjustment, baseline-adjusted linear regression models (which are also adjusted for randomisation group, clinical strata groups A, B, and C, history of COVID-19 pneumonia, age, and sex) will be used to analyse these outcomes. For continuous data which are not Normally distributed, standard transformations will be used to achieve approximate Normality prior to analysis. Checks of the model fit will include inspection of Normal Q-Q plots and assessing collinearity.

Ordinal outcomes (e.g. frailty measures) will be analysed using an ordinal logistic regression model. The proportional odds assumption will be checked using a likelihood ratio test which compares the model used to one where treatment group is a nominal effect. The model will be summarised with odds ratios and corresponding 95% confidence intervals. To check the fit of the models, plots of observed versus fitted values be inspected.

2.4.3. Exploratory Outcomes

The exploratory outcomes are listed below. Each of these will be analysed with an appropriate regression method unless otherwise stated.

1) Exercise dose achieved as detailed by activity level attained, and within-subject change from baseline. This information is only available for subjects randomised to the intervention so the outcome will be presented with descriptive analysis for the intervention group only.

2) Vital parameters of cardio-respiratory function (e.g. oxygen saturation, heart rate, respiratory rate) at baseline and follow-up.

3) Adherence with exercise. This information is only available for subjects randomised to the intervention so the outcome will be analysed for the intervention group only.

Docusign Envelope ID: AF5020A8-A0EF-446D-839B-4F824F00D5A0

4) Accelerometry (Glasgow sites only). Note that as this outcome relates to a substudy, the analysis may be reported separately to the main study publication.

5) Biobank tissue samples for biomedical research. Note that this outcome is not covered by this SAP.

6) Optional observational substudy of small vessel function using myography in arterioles isolated from gluteal skin biopsies and laboratory analysis of adipose tissue, cells, and molecules from the biopsy. Note that this outcome is not covered by this SAP.

7) Exploratory graphical analyses of COVID-19 immune serology (as assessed at 3 month follow-up) and its association with illness severity, as reflected by biomarkers (e.g. NTproBNP, HbA1C, BMI), functional markers (e.g. handgrip strength, SPPB, spirometry), and patient reported outcome measures (e.g. EQ-5D, iPAQ, DASI).

2.4.4 Sub-group Analysis

The primary outcome analysis will be repeated to assess if the treatment effect varies between levels in the following subgroups:

- Clinical presentation groups
- Sex
- SIMD decile
- Ethnic group
- COVID-19 serology.

2.5. Safety Outcomes

2.5.1. Premature Withdrawal

Patient withdrawal is recorded in the eCRF. Dates and reasons for withdrawal from the intervention or long-term follow-up are recorded. A listing will be created which will include details of withdrawals, and withdrawal data will be summarised as part of the final report.

2.5.2. Serious Adverse Events

Serious adverse events are captured within the eCRF for review by the chief investigator. Such events have date of event, level of severity, and diagnosis recorded. Any concomitant medications, relevant tests, or medical history may also be recorded. There are no specific adverse events of interest for this study. A listing will be created including details of all SAEs, and SAE data will be summarised and presented as part of the final report, split by treatment group.

Docusign Envelope ID: AF5020A8-A0EF-446D-839B-4F824F00D5A0

3. Data Conventions

A separate assumptions document detailing data rules (e.g. partial dates, missing data imputation) will be created and saved to the following location: \\RCB-STORAGE\filestore\Studies\CISCO21\StudyDiary\StatsDocs\Assumptions.

4. Tables and Figures

A table shells document will be prepared and stored in the following location: \\RCB-STORAGE\filestore\Studies\CISCO21\StudyDiary\StatsDocs\SAP\TableShells.

5. Document History

This is the first version (v1.0) of this document (initial creation).

docusign

Certificate Of Completion

Envelope Id: AF5020A8A0EF446D839B4F824F00D5A0		Status: Completed
Subject: Complete with Docusign: CISCO21_SAP_v1_0_CBsigned.docx		
Source Envelope:		
Document Pages: 10	Signatures: 2	Envelope Originator:
Certificate Pages: 2	Initials: 0	Gemma McKinley
AutoNav: Enabled		University of Glasgow, University Avenue
EnvelopeId Stamping: Enabled		Glasgow, North Lanarkshire G12 8QQ
Time Zone: (UTC) Dublin, Edinburgh, Lisbon, London		Gemma.McKinley@glasgow.ac.uk
		IP Address: 130.209.6.41

Record Tracking

Status: Original<br>07-11-2024 | 08:51	Holder: Gemma McKinley<br>Gemma.McKinley@glasgow.ac.uk	Location: DocuSign

Signer Events	Signature	Timestamp
Alex McConnachie<br>alex.mcconnachie@glasgow.ac.uk<br>Professor of Clinical Trial Biostatistics<br>University of Glasgow<br>Security Level: Email, Account Authentication (None)	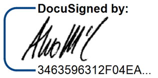<br>Signature Adoption: Uploaded Signature Image<br>Using IP Address: 130.209.6.41	Sent: 07-11-2024 | 08:52<br>Viewed: 07-11-2024 | 08:53<br>Signed: 07-11-2024 | 08:53<br>Freeform Signing

Electronic Record and Signature Disclosure:
Not Offered via DocuSign

Gemma McKinley<br>gemma.mckinley@glasgow.ac.uk<br>Junior Biostatistician<br>University of Glasgow<br>Security Level: Email, Account Authentication (None)	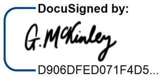<br>Signature Adoption: Uploaded Signature Image<br>Using IP Address: 130.209.6.41	Sent: 07-11-2024 | 08:52<br>Viewed: 07-11-2024 | 08:52<br>Signed: 07-11-2024 | 08:53<br>Freeform Signing

Electronic Record and Signature Disclosure:
Not Offered via DocuSign

In Person Signer Events	Signature	Timestamp

Editor Delivery Events	Status	Timestamp

Agent Delivery Events	Status	Timestamp

Intermediary Delivery Events	Status	Timestamp

Certified Delivery Events	Status	Timestamp

Carbon Copy Events	Status	Timestamp

Witness Events	Signature	Timestamp

Notary Events	Signature	Timestamp

Envelope Summary Events	Status	Timestamps
Envelope Sent	Hashed/Encrypted	07-11-2024 | 08:52
Certified Delivered	Security Checked	07-11-2024 | 08:52
Signing Complete	Security Checked	07-11-2024 | 08:53

Envelope Summary Events	Status	Timestamps
Completed	Security Checked	07-11-2024 | 08:53

Payment Events	Status	Timestamps
