# Supplementary material for: Resistance Exercise Therapy After COVID-19 Infection: A Randomized Clinical Trial
Source: JAMA Netw Open. 2025 Nov 10;8(11):e2534304. doi: 10.1001/jamanetworkopen.2025.34304 (PMC12603858; doi:10.1001/jamanetworkopen.2025.34304)
Supplement: Supplement 2. — eAppendix 1. Abbreviations eAppendix 2. Protocol Amendments eMethods. eDiscussion. eTable 1. Schedule of Enrollment, Interventions and Assessments eTable 2. Number of Participants Randomized Under Each Protocol Version at Each Site eTable 3. Baseline Characteristics: COVID-19 Treatment eTable 4. COVID-19 Reinfection and Vaccination eTable 5. Primary Outcome: Incremental Shuttle Walk Test Distance (m) at Baseline eTable 6. Primary Outcome: Incremental Shuttle Walk Test Distance (m) at 3 Months eTable 7. Primary Outcome Analysis: Incremental Shuttle Walk Test Distance (m) With Imputed Data eTable 8. Primary Outcome - Causal Effect Analysis at Various Compliance Levels eTable 9. Primary Outcome: Incremental Shuttle Walk Test in subjects With Good Exercise Adherence (>70%, Intervention Group Only) by Clinical Presentation Group eTable 10. Secondary Outcome Analysis: Spirometry - Peak Expiratory Flow Rate (L/m) eTable 11. Secondary Outcome Analysis: Spirometry - Forced Vital Capacity (L) eTable 12. Secondary Outcome Analysis: Spirometry - Forced Expiratory Volume in One Second (L) eTable 13. Secondary Outcome Analysis: Spirometry - FEV1/FVC Ratio eTable 14. Secondary Outcome Analysis: Handgrip Strength (kg) eTable 15. Secondary Outcome Analysis: Short Physical Performance Battery Score Category eTable 16. Secondary Outcome Analysis: EQ-5D-5L Utility Score (UK Crosswalk Value Set) eTable 17. Secondary Outcome Analysis: EQ-5D-5L Visual Analogue Scale eTable 18. Secondary Outcome Analysis: Patient Health Questionnaire Category eTable 19. Secondary Outcome Analysis: Brief Illness Perception Questionnaire Score eTable 20. Secondary Outcome Analysis: Duke Activity Status Index Score eTable 21. Secondary Outcome Analysis: Duke Activity Status Index Predicted VO2max eTable 22. Secondary Outcome Analysis: International Physical Activity Questionnaire (Short Form) eTable 23. Secondary Outcome Analysis: Fatigue Severity Score eTable 24. Secondary Outcome Analysis: MRC Dyspnea Score [file jamanetwopen-e2534304-s002.pdf]

Supplementary Online Content

Berry C, McKinley G, Bayes HK, et al. Resistance exercise therapy after COVID-19 infection: a randomized clinical trial. *JAMA Netw Open.* 2025;8(9):e2534304. doi:10.1001/jamanetworkopen.2025.34304

eAppendix 1. Abbreviations

eAppendix 2. Protocol Amendments

eMethods.

eDiscussion.

eTable 1. Schedule of Enrollment, Interventions and Assessments

eTable 2. Number of Participants Randomized Under Each Protocol Version at Each Site

eTable 3. Baseline Characteristics: COVID-19 Treatment

eTable 4. COVID-19 Reinfection and Vaccination

eTable 5. Primary Outcome: Incremental Shuttle Walk Test Distance (m) at Baseline

eTable 6. Primary Outcome: Incremental Shuttle Walk Test Distance (m) at 3 Months

eTable 7. Primary Outcome Analysis: Incremental Shuttle Walk Test Distance (m) With Imputed Data

eTable 8. Primary Outcome - Causal Effect Analysis at Various Compliance Levels

eTable 9. Primary Outcome: Incremental Shuttle Walk Test in subjects With Good Exercise Adherence (>70%, Intervention Group Only) by Clinical Presentation Group

eTable 10. Secondary Outcome Analysis: Spirometry - Peak Expiratory Flow Rate (L/m)

eTable 11. Secondary Outcome Analysis: Spirometry - Forced Vital Capacity (L)

eTable 12. Secondary Outcome Analysis: Spirometry - Forced Expiratory Volume in One Second (L)

eTable 13. Secondary Outcome Analysis: Spirometry - FEV1/FVC Ratio

eTable 14. Secondary Outcome Analysis: Handgrip Strength (kg)

eTable 15. Secondary Outcome Analysis: Short Physical Performance Battery Score Category

eTable 16. Secondary Outcome Analysis: EQ-5D-5L Utility Score (UK Crosswalk Value Set)

eTable 17. Secondary Outcome Analysis: EQ-5D-5L Visual Analogue Scale

eTable 18. Secondary Outcome Analysis: Patient Health Questionnaire Category

© 2025 Berry C et al. *JAMA Network Open.*

eTable 19. Secondary Outcome Analysis: Brief Illness Perception Questionnaire Score

eTable 20. Secondary Outcome Analysis: Duke Activity Status Index Score

eTable 21. Secondary Outcome Analysis: Duke Activity Status Index Predicted VO2max

eTable 22. Secondary Outcome Analysis: International Physical Activity Questionnaire (Short Form)

eTable 23. Secondary Outcome Analysis: Fatigue Severity Score

eTable 24. Secondary Outcome Analysis: MRC Dyspnea Score

eTable 25. Secondary Outcome Analysis: Fried Frailty (Sum of Phenotypes)

eTable 26. Post-Exertional Malaise at 3-Months, Part 1 (DePaul Symptom Questionnaire, Short Form)

eTable 27. Post-Exertional Malaise at 3-Months, Part 2 (DePaul Symptom Questionnaire, Short Form) Additional Questions

eTable 28. Post-Exertional Malaise at 3-Months, Part 3 (DePaul Symptom Questionnaire, Short Form) Composite Measures

eTable 29. 3-Month Follow-up Data: Secondary Outcomes: Accelerometry - Changes From Baseline

eTable 30. Secondary Outcome Analysis: hospitalization for any Reason (as Recorded in SAEs)

eTable 31. Visits Involving Physiotherapy and Rehabilitation in the Community

eFigure 1. The Distribution in the Distances (m) Achieved During the Incremental Shuttle Walk Test at Baseline, 3-Months Post-Randomization, and the Change at 3-Months From Baseline

eFigure 2. Cohen's D Forest Plot Depicting Standardized Intervention Effect Estimates and 95% Confidence Intervals for Primary and Secondary Outcome Measures

eFigure 3. Primary Outcome - Incremental Shuttle Walk Test Distance Intervention Effect estimates From Complier Average Causal Effects Analyses

eFigure 4. EuroQol-5D-5L Life Utility Score (EQ-5D-5L)

eFigure 5. Patient Health Questionnaire-4 (PHQ-4) Category

eFigure 6. Handgrip Strength

This supplementary material has been provided by the authors to give readers additional information about their work.

eAppendix 1. Abbreviations

Incremental shuttle walk test (ISWT)

Short performance physical battery (SPPB)

National Health Service (NHS)

Research Ethics Committee (REC)

Severe angiotensin converting enzyme-2 (ACE2)

Acute respiratory syndrome coronavirus-2 (SARS-CoV-2)

Coronavirus disease-19 (COVID-19)

Cardiovascular disease (CVD)

National Institute for Health and Care Excellence (NICE)

Scottish Intercollegiate Guidelines Network (SIGN)

Royal College of General Practitioners (RCGP)

Cardiac Imaging in SARS-CoV-2 (COVID-19) (CISCO-19)

Polymerase Chain Reaction (PCR)

Intensive care unit (ICU)

NHS Greater Glasgow and Clyde (NHSGGC)

Rating of Perceived Exertion (RPE)

Patient and Public Involvement (PPI)

Adverse event (AE)

© 2025 Berry C et al. *JAMA Network Open.*

Serious adverse event (SAE)

Electronic case report form (eCRF)

Post-hospitalization COVID-19 (PHOSP-COVID)

Brief Illness perception questionnaire (Brief IPQ)

Duke Activity Status Index (DASI)

International Physical Activity Questionnaire (IPAQ-SF)

Euroqol-5 dimension (EQ5D)

Medical Research Council (MRC)

Clinical Trials Unit (CTU)

Standard operating procedures (SOPs)

Statistical Analysis Plan (SAP)

Consolidated Standards of Reporting Trials (CONSORT)

Principal Investigator (PI)

Related Unexpected Serious Adverse Event (RUSAE)

Clinical Trial of an Investigational Medicinal Product (CTIMP)

Post-exertional malaise (PEM)

Graded exercise therapy (GET)

Myalgic encephalomyelitis or chronic fatigue syndrome (ME/CFS)

Robertson Centre for Biostatistics (RCB)

© 2025 Berry C et al. *JAMA Network Open.*

eAppendix 2. Protocol Amendments

List of amendments

Amendment no.	Date	Reason
01	19.5.21	Addition of 2 researchers to the study.
02	07.10.21	Addition of Participant Identification Centers (PIC) for the first time.
03	10.02.22	Changes to protocol: removal of section for an independent data monitoring committee (IDMC), revision of the screening and recruitment section. Updates to inclusion & exclusion criteria.<br>Addition of PIC sites recruiting through Primary Care services.<br>Addition of Gluteal Biopsy Sub Study.
04	11.05.22	Changes to the Sub Study patient information sheet and Consent Forms.
05	23.05.22	Change of date on the sub study patient information sheet
06	08.07.22	Changes to protocol: update inclusion/exclusion criteria to meet changes in Covid testing in the general population.<br>Extend the time from being diagnosed with Covid-19 to enrolment in the study from 6 months to 12 months.<br>Addition of text regarding Post Exertion Malaise (PEM). Patient information sheet and consent updated to reflect these changes.

© 2025 Berry C et al. *JAMA Network Open.*

07	14.12.22	Update of wording in the study protocol regarding eligibility.
08	16.12.22	Update the date of the patient information sheet on the corresponding consent form.
09	03.04.23	No cost extension granter by the funder until 30th September 2022 to facilitate completion of recruitment.
10	29.05.23	Changes to the protocol: update to the inclusion criteria to meet changes in the Covid-19 testing of the general population – no longer routinely tested a request to allow recruitment of patients on the basis of either a positive polymerase chain reaction test, or of a lateral flow test, confirmed either from their medical records or by the patient confirming that they have had a positive lateral flow test, or of a positive antibody test. The time from having a positive Covid-19 test that the patient can be recruited was removed. The patient information sheet was revised to reflect these changes.
11	11	Addition of two further Participant Identification Centers. Updated wording on study poster to reflect.
12	02.77.23	No cost extension granted by the funder until 1 October 2024 to ensure recruitment to target.
13	11.01.24	Increase in sample size by 20 to ensure that the minimum number of randomized participants have primary outcome data at 3 months post-enrolment.

© 2025 Berry C et al. *JAMA Network Open.*

eMethods.

Study Design

A multicenter, parallel group, 1:1 randomized, controlled trial of resistance exercise in adults with a diagnosis of COVID-19 in the preceding 12 months and persisting symptoms was undertaken in Scotland. Ethical approval was granted by the National Health Service (NHS) Research Ethics Committee (reference: GN20CA537). The protocol schedule and amendments are described in the Supplement (Tables S1-S2, respectively) and the study design has been published[12]. The study has been reported according to the CONSORT reporting guideline for randomized, controlled trials [Figure 1][13].

All participants provided written informed consent. The study was publicly registered before the first participant was randomized (Clinicialtrials.gov ID NCT04900961).

Setting and Population

Setting

The sites were the Queen Elizabeth University Hospital and Royal Infirmary in Glasgow in the west of Scotland and Ninewells Hospital in Dundee in the east of Scotland.

Population

Participants were classified according to being not hospitalized due to COVID-19 but as having persisting symptoms for at least 4 weeks leading to medical review (*Group A*); discharged after hospitalization for COVID-19 and with persistent symptoms for at least 4 weeks (*Group B*); or convalescing in hospital after hospitalization for COVID-19 (*Group C*).

Participants enrolled in the community were included in Groups A and B whereas participants enrolled during hospital admission were in Group C.

Eligibility criteria

Inclusion criteria

1) Diagnosis of COVID-19 confirmed by:
    a) Virology PCR-positive laboratory diagnosis and/or point of care test positive for COVID-19, or,
    b) Positive lateral flow test, or,
    c) Positive COVID antibody test;
2) Within 12-months of diagnosis;
3) Persistent symptoms for at least 4 weeks from symptoms onset (Groups A & B only);
4) Presentation type - one of group A, B or C.

Exclusion criteria

1) Inpatient physiotherapy currently part of standard care post-ICU;
2) No expectation of being able to walk within three months;
3) Unable to provide informed consent;
4) Unable to comply with the protocol;
5) Known pregnancy.

© 2025 Berry C et al. *JAMA Network Open.*

On 14 December 2022, given the reduction in incident cases of COVID-19 in the community, the initial eligibility period of 6 months from the diagnosis of COVID-19 (protocol versions 1, 2 and 3) was extended to 12 months (protocol versions 4).

Control group

The comparator was usual care (treatment-as-usual) for Long Covid in line with guidelines from the National Institute for Healthcare Excellence 188 guideline[2], with no non-routine contacts from research staff.

Intervention

An exercise program was co-designed by exercise physiologists, physiotherapists and individuals with lived experience of Long COVID. The intervention was developed through discussions with patient groups around the needs of the individual during the exercise intervention, such as staff contact, need for a seated (chair-based) exercise option, and personalization of the exercises, followed by practical exercise sessions involving patients who were hospitalized with COVID-19.

For participants assigned to the intervention group, an instructional pack was provided by research staff supported by an exercise physiologist (S.G.). The pack included a guidance document, an exercise log and links to online videos (Supplement). The pack was given to participants during an initial face-to-face consultation where the nurse/therapist helped to select the most suitable category and level of exercise for the participant, demonstrate the exercises and ensure the participant was comfortable performing the exercise options. Every 2 weeks, each participant was contacted by the research team by telephone or video consultation to provide guidance and support to the participant. If the participant was in hospital then the contact was undertaken daily, as needed.

© 2025 Berry C et al. *JAMA Network Open.*

The intervention occurred in the community (Groups A and B) or in-hospital and then at home after discharge (Group C). Participants were asked to perform exercises daily. The number of exercise repetitions that led to a validated resistance exercise specific Rating of Perceived Exertion (RPE) of 8-10 (3-5 in the first week) was determined[14]. The use of RPE to prescribe and titrate resistance exercise is as efficacious as more complex methods but reported to be better tolerated[15]. The intervention was tailored according to the preferences of the participant and progress achieved. Additional information is described in the Supplement.

There were 3 exercise categories and guidance was provided according to the status of the participant:

- Bed-bound: lying chest-press, lying row, lying plantar flexion, lying leg press and bridging.
- Up-to-sit: seated chest-press, seated row, seated lateral raises, seated leg extension, seated plantar flexion, squats.
- Ambulatory: press-ups, standing lateral raises, seated rows, lunges, calf-raises, squats.

Participants were asked to perform upper body exercise initially and bring-in the lower body exercises in week 3. The exercise log was intended to be completed by the participant after an episode of exercise activity had been undertaken, whether it was completed and whether any adverse effects occurred.

Randomization and Blinding

The details of randomization and blinding are described in detail in the Supplement. Participants were allocated in a 1:1 ratio to usual care or usual care plus a personalized resistance exercise intervention for 12-weeks. Allocations were determined using a minimization algorithm with a small random element, designed to ensure balance with respect to study site, clinical presentation

group (A, B, or C), history of COVID pneumonia, age (<40, 40-49, 50-59, 60-69. 70+), and sex. Allocations were assigned after baseline data collection, via the study-specific online data collection tool, developed and maintained by staff at the Robertson Centre for Biostatistics, University of Glasgow. Participants and staff delivering the intervention were unblinded to group allocations, but statistical staff were blind until the point of database lock. The Statistical Analysis Plan was finalized, and all statistical programs were written and validated prior to database lock and unblinding.

Outcomes

Primary outcome

The primary outcome was the distance achieved (m) during the incremental shuttle walk test. This is an externally paced incremental walking test developed as a measure of exercise capacity[16].

Participants were required to walk around two marker cones, 9 m apart, placed 0.5 m from each endpoint (10m course) with an initial speed of 0.5m/s, increasing 0.17m/s every minute. Audio cues (beeps) signal the time at which the participant should turn at the marker. The test has 12 levels (walking speeds), and the maximum duration of the test is therefore 12 minutes. No encouragement was given during the test: the only verbal cues provided referred to an impending increase in walking speed[17]. The incremental shuttle walk test performance was defined as the distance achieved[17] and oxygen saturation and heart rate were measured[18].

The duration of the incremental shuttle walk test correlates with peak oxygen consumption (ml/min/kg) and has population reference values for distance walked (m)[19] . The test has been evaluated and validated in several populations, including in healthy women[20], young men[21], in obese individuals[22] and patients with chronic respiratory disease[14, 16-18]. The incremental shuttle

© 2025 Berry C et al. *JAMA Network Open.*

walk test is recognized for being safe and responsive to the effects of rehabilitation in populations with chronic respiratory disease[18] and stakeholder organizations support the use of this test as an efficacy measure in clinical trials[19].

Secondary outcomes

The following secondary outcomes were assessed:

Respiratory function

1. Spirometry

Physical function

2. Handgrip strength
3. Short Physical Performance Battery (SPPB)[23]

Patient reported outcome measures

4. Health-related quality of life (Euroqol-5 dimension (EQ5D-5L))[24]
5. Patient health questionnaire-4 (PHQ-4)[25]
6. Illness perception (Brief IPQ)[26]
7. Duke Activity Status Index (DASI)[27]
8. International Physical Activity Questionnaire (IPAQ-SF) short-form[28]

Fatigue

9. Medical Research Council (MRC) dyspnea score[29]

© 2025 Berry C et al. *JAMA Network Open.*

Frailty

10. Fried frailty phenotype: five criteria: weight loss; exhaustion; grip strength; low physical activity; and slow walking pace[30]

11. Clinical Frailty Scale[31]

Clinical outcomes and adverse events

12. Episodes of healthcare

13. Hospitalization for any reason.

Additional prespecified outcomes

1. Vital parameters of cardio-respiratory function e.g. oxygen saturation, heart rate, respiratory rate at baseline and during follow-up

2. Adherence with exercise (intervention group)

3. Post-exercise adverse events and malaise: adverse events during and after exercise were assessed in all participants. Following a protocol amendment, the DePaul Symptom Questionnaire (Short Form)[32] was assessed in a subgroup.

4. Accelerometry (Glasgow site) - Participants were issued with a GENEActiv (ActivInsights Ltd, United Kingdom) accelerometer and instructed to wear this 24 h per day for a 7-day period. The accelerometer was set to record at 100 Hz. Acceleration data were collected and calibrated to local gravity[33-37] and physical activity levels were quantified using GGIR, with methods previously described[35]. A valid day was defined as having >16 hours of data in it, and we excluded participants with less than 3 valid days of data or if wear data

© 2025 Berry C et al. *JAMA Network Open.*

were not present for every 15 minutes of the 24-hour cycle, to ensure data was representative of a complete seven-day measure[36].

5. COVID-19 serology using the SARS-CoV-2 IgG II Quant assay[38].

COVID-19 serology

The SARS-CoV-2 IgG II Quant assay is a chemiluminescent microparticle immunoassay (CMIA) used for the qualitative and quantitative determination of IgG antibodies to SARS-CoV-2 in human serum and plasma on the ARCHITECT i System [38].

The SARS-CoV-2 IgG II Quant assay was used to assess for prior SARS-CoV-2 infection in conjunction with the clinical history and other laboratory tests. The assay was also used as an aid in evaluating immune status of infected individuals. The antibody response also reflects prior COVID-19 vaccination, by quantitatively measuring IgG antibodies against the spike receptor-binding domain (RBD) of SARS-CoV-2.

The SARS-CoV-2 IgG II Quant assay is an automated, two-step immunoassay for the qualitative and quantitative determination of IgG antibodies to SARS-CoV-2 in human serum and plasma using chemiluminescent microparticle immunoassay (CMIA) technology.

Sample, SARS-CoV-2 antigen coated paramagnetic microparticles, and assay diluent are combined and incubated. The IgG antibodies to SARS-CoV-2 present in the sample bind to the SARS-CoV-2 antigen coated microparticles. The mixture was then washed. Anti-human IgG acridinium-labeled conjugate was added to create a reaction mixture and then incubated. Following a wash cycle, Pre-Trigger and Trigger Solutions were added. The resulting chemiluminescent reaction was measured as a relative light unit (RLU). There is a direct relationship between the amount of IgG antibodies to SARS-CoV-2 in the sample and the RLU .

© 2025 Berry C et al. *JAMA Network Open.*

Bias minimization

Bias minimization procedures involved random allocation of the participants, masking of participants at the time of follow-up to baseline results, and retention strategies, such as by providing follow-up contacts to support the participants and minimize withdrawals. Outcome assessors were not blinded to group allocation.

Recruitment

Patients presenting at participating acute hospitals in the cities of Glasgow and Dundee and in Primary Care settings e.g. General Practice, community assessment centers, Community Health Centers or Community Test Centers, with COVID-19 could have been considered for inclusion. A screening log with the reasons for not being enrolled, was prospectively completed.

Recruitment from Primary Care was facilitated by advertising in existing networks (e.g. Long COVID Scotland groups). Recruitment from General Practice was facilitated by providing information about the study i.e. poster, patient information sheet, consent form.

The study had an open approach to participation to best ensure being representative of patients with post-COVID-19 conditions receiving usual care. The results are intended to have external relevance and be transferable to clinical practice. The open approach to enrolment was intended to facilitate timely delivery of the trial.

Assignment of interventions: allocation

Sequence generation

Participants were allocated to the control group (usual care) or the intervention group (usual care plus resistance exercise) using a mixed minimization/randomization procedure. Within each study

site, out of every ten participants, 8 were allocated according to a minimization algorithm, designed to maintain balance with respect to study site, age (<40, 40-49, 50-59, 60-69, or ≥70 years), sex, clinical presentation group (A, B, or C), and history of COVID-19 pneumonia. The remaining two participants in each block of ten were allocated at random. The sequence in which participants were allocated by minimization or randomization was computer-generated in advance and known only to those responsible for maintaining the allocation system.

Concealment mechanism

Allocations were performed via a study-specific web portal, at the end of the enrolment visit, after collection of baseline data. The research nurse requesting each allocation was unaware of the allocation group in advance of enrolling and randomizing the group allocation.

Implementation

Participants were enrolled on the randomization system by research nurses who were automatically and immediately informed of the intervention allocation by email.

Assignment of interventions: Blinding

The trial statisticians were blind to allocation. As this is an exercise intervention blinding of the participants or research staff, who instructed the participants in the intervention, was not possible. Since the allocation was open-label for the participants and site staff, there was no unblinding system.

Data collection and management

Plans for assessment and collection of outcomes

Data management followed the processes of the Glasgow Clinical Trials Unit: http://glasgowclinicaltrials.org/sops/. Data were collected via a study-specific electronic case report form (eCRF), developed by the Robertson Centre for Biostatistics (RCB), University of Glasgow (within the Glasgow Clinical Trials Unit). The eCRF included built-in logic steps and point of entry validation for selected fields. Data management staff ran validation checks on the data during the trial, according to a pre-specified Data Management Plan. The statistical staff also had an oversight of data quality. The database was only locked for the final analysis after all data management and statistical data validation checks had been resolved.

Participant retention

Participant retention was promoted by research staff at the outset of participating in the study and again during the follow-up contacts.

Data management

All data handling procedures were detailed in a study-specific data management plan. All quantitative data collected as part of the study were securely transferred to the Robertson Centre for Biostatistics Clinical Trials Unit (CTU) for data entry and checking in accordance with their standard operating procedures (SOPs).

Data were validated at regular intervals during the study. Data discrepancies were flagged to the clinical research site, and any data changes were recorded to maintain an audit trail and the reasons

© 2025 Berry C et al. *JAMA Network Open.*

for changes in the data, including the date of the change and who made change, were prospectively recorded.

Statistics

Sample size calculation

A pre-determined sample size calculation was devised by biostatistician co-authors. Taking the minimum clinically important between-group difference in the incremental shuttle walk test at follow-up (3-months) to be 46 m, with a SD of 105m[16], the sample size required for 80% power, at 5% significance, with no loss to follow-up (LTFU) was 85 per group; allowing for LTFU and incomplete data, the target sample size was 110 per group (220 total) randomized..

Statistical analyses

The primary and secondary outcomes were analyzed using linear regression (continuous outcomes), or proportional odds logistic regression (ordinal outcomes). All models were adjusted for prespecified characteristics at baseline including age, sex, clinical presentation, history of COVID-19 pneumonia, site, and the baseline value of the analysis variable where this was recorded. Regression models were used to assess treatment effects within pre-specified subgroups outlined in the Statistical Analysis Plan, using treatment-by-subgroup interactions. For the primary outcome, a sensitivity analysis was performed with multiple imputations for the missing outcomes.

Tests of proportional odds assumption (for treatment group) were used for categorical models. All tests were two-tailed and assessed at the 5% significance level. Missing outcome data were not imputed for the main analysis. As multiplicity was not adjusted for in the analyses of secondary outcomes, these outcomes should be interpreted as exploratory. The statistical analyses were

© 2025 Berry C et al. *JAMA Network Open.*

conducted using R Studio and R version 4.0.0 (R Foundation for Statistical Computing, Vienna, Austria) according to a pre-specified Statistical Analysis Plan (SAP).

Confidentiality

Access to the collated participants' data was restricted to the principal investigator and designated research study staff at the sites. All laboratory samples, completed forms, reports and other records were identified using a unique participant ID number to maintain participant confidentiality.

Blood samples

A single blood was collected at 3 months according to a sample handling manual approved by the sponsor. The plasma was stored at -80◦C in a secure biorepository for future analysis.

Statistical methods

Statistical methods for primary and secondary outcomes

This trial had a comprehensive Statistical Analysis Plan, which governed all statistical aspects of the study, and was authored by the Trial Statistician before the database lock and unblinding of the statistical team. The Statistical Analysis Plan was based on intention to treat principles in line with the Consolidated Standards of Reporting Trials (CONSORT) guidelines. The analysis focused on estimation of treatment effect differences with 95% confidence intervals and p-values. All the pre-specified secondary outcome analyses will be reported in study publications.

© 2025 Berry C et al. *JAMA Network Open.*

Primary efficacy analysis

Alternative hypothesis

In patients with persisting symptoms during the convalescence phase after COVID-19, resistance exercise increases exercise capacity measured by the incremental shuttle walk test. The null hypothesis reflects no difference in exercise capacity between the groups.

The primary outcome (incremental shuttle walk test distance at 3 months) was analyzed using a linear regression model, with randomized group, baseline incremental shuttle walk test distance, age, sex, clinical presentation group, and history of COVID-19 pneumonia as covariates. Similar methods were applied to secondary outcomes, using linear, binary logistic, or ordinal logistic regression methods, as appropriate.

Interim analyses

No interim analyses occurred during the study.

Methods for additional analyses

Analyses of the primary outcome were done in relation to subgrouping variables including age, sex, clinical presentation group, and history of COVID-19 pneumonia. This involved extending the primary analysis regression model to include interaction terms. Complier Average Causal Effects analysis will be employed to estimate the effect of the exercise intervention amongst those who were able to comply with it.

Methods to handle protocol non-adherence and statistical methods to handle missing data

Participants found to be ineligible for the study, or who were randomized in error, were excluded from all analyses. Other forms of non-compliance that can be identified from the study data (e.g.

© 2025 Berry C et al. *JAMA Network Open.*

visits out with pre-specified windows) were reviewed (blind to group allocation) and classified as major or minor. Similarly, any other protocol non-compliances were assessed prior to locking the database and classified as major or minor.

Missing data were not imputed for the primary outcome analysis. As sensitivity analyses, these analyses were repeated, using multiple imputations with chained equations for missing (Table S7. Primary outcome analysis: Incremental shuttle walk test distance (m) with imputed data).

Plans to give access to the full protocol, participant level-data and statistical code

The data sets and codes that were used for these analyses are available from the corresponding author on reasonable request.

Oversight and monitoring

Composition of the coordinating center and trial steering committee

The Trial Steering Committee comprised of:

Professor Susan Dawkes (Chair)

Professor Sally Singh (Independent member)

Dr Hannah Bayes (Non-Independent member)

Professor Colin Berry (Chief Investigator)

Professor Stuart Gray (Non-Independent member)

Dr Tracy Ibbotson (Independent member; patient and public involvement representative)

Professor Alex McConnachie (Study Statistician)

Dr Maureen Travers (Sponsor representative); Dr Alison Hamilton (Sponsor representative)

© 2025 Berry C et al. *JAMA Network Open.*

Ms Diann Taggart (Study Project Manager)

Role of sponsor

The sponsor had no role in the study design; collection, management, analysis, and interpretation of data; writing of the report; or the decision to submit the report for publication, and no ultimate authority over any of these activities.

Data monitoring committee

Since the intervention was deemed low-risk and the trial did not involve an investigational medicinal product the sponsor deemed that a data monitoring committee was not needed.

Adverse event reporting and harms

Where a serious adverse event (SAE) occurred then full details were recorded, including the nature of the event, the start and end dates, the severity grade, the relationship to the trial procedure, and the outcome of the event were recorded in the participant's medical notes and the electronic case report form (eCRF). These events were monitored and followed up until the event had resolved and stabilized. All SAEs were recorded in the eCRF within 24 hours of the site staff becoming aware of the event and the staff provided additional follow-up information as soon as this became available. The Site Principal Investigator (PI) used his/her medical judgement in assigning seriousness, causality, severity and expectedness with reference to the trial protocol. Relatedness and expectedness were determined by the site team and site principal investigator. The sponsor verified data collection and SAEs, according to the trial protocol. The sponsor reported safety information to the Chief Investigator for the ongoing assessment of the risk / benefit and in collaboration with the Chief Investigator the annual safety report to the Research Ethics Committee and until the End of the Study, at which time an End of Study form, were submitted. The Trial

© 2025 Berry C et al. *JAMA Network Open.*

Steering Committee reviewed safety in accordance with a predefined charter, periodically reviewing recruitment and the overall progress of the trial and liaising with the sponsor regarding safety issues.

The study Data Center provided an eCRF for central data collection of AEs and SAEs and they were reported to the Trial Steering Committee which typically met on a 6-monthly basis until the end of the study.

Frequency and plans for audit

An audit of all entries into the eCRF was deemed to be a sufficient monitoring plan for a non-Clinical Trial of an Investigational Medicinal Product (CTIMP).

Communication of protocol amendments to relevant parties

All amendments to the protocol were approved by the local ethics committee prior to implementation and the Clinicatrials.gov registration was updated. All investigators were informed.

Any deviations from the Protocol were documented using a report form.

Dissemination

The study findings will be disseminated primarily via conference presentations, scientific papers, social media and public engagement activities. All authors read and approved of the final manuscript.

Funding

The study was funded by a grant from the Chief Scientist Office Scotland (Grant reference: COV/LTE/20/10) and the University of Glasgow British Heart Foundation Centre of Research Excellence (RE/18/634217)

Data availability

Data requests will be considered by the Steering Group, which includes representatives of the sponsor, the University of Glasgow, senior investigators independent of the research team and the chief investigator. The Steering Group took account of the scientific rationale, ethics, logistics and resource implications.

Data access requests should be initially submitted by email to the chief investigator (C.B., corresponding author). The source data include the de-identified numerical data used for the statistical analyses. Data access will be provided through the secure analytical platform of the Robertson Centre for Biostatistics. This secure platform enables access to de-identified data for analytical purposes, without the possibility of removing the data from the server. Requests for transfer of de-identified data will be considered by the Steering Group, and, if approved, a collaboration agreement will be expected. The Steering Group will consider any cost implications, and cost recovery would be expected on a not-for-profit basis.

Competing interests

The authors declare that they have no competing interests.

© 2025 Berry C et al. *JAMA Network Open.*

eDiscussion.

Comparisons with prior studies

In the Rehabilitation Exercise and psycholoGical support After COVID-19 InfectioN (REGAIN) trial[49], 585 adults (26-86 years) discharged from UK hospitals at least three months previously after COVID-19 and with ongoing physical and/or mental health sequelae (post-COVID-19 condition) were randomized 1:1 to receive the intervention (n=298) or usual care (n=287). The REGAIN intervention was delivered online over eight weeks and consisted of weekly home based, live, supervised, group exercise and psychological support sessions. Compared with usual care, the intervention improved health-related quality of life (adjusted mean difference in the patient reported outcomes measurement information system (PROMIS) preference score 0.03 (95% confidence interval 0.01 to 0.05), P=0.02) at three months and the effects were sustained at 12-months (0.03 (0.01 to 0.06), P=0.02). In a systematic review[50], rehabilitation interventions were associated with improvements in functional exercise capacity (standard mean differences, -0.56; 95% credible intervals, -0.87 to -0.22), however, there was uncertainty and imprecision regarding the probability of experiencing exercise-induced adverse events (odds ratio, 1.68; 95% credible intervals, 0.32 to 9.94).

More recently, the Post-Hospitalisation COVID-19 Rehabilitation (PHOSP-R) consortium reported a trial of exercise-based rehabilitation in 181 participants (55% male, mean [SD] age 59 [12] years, duration of hospitalization 12 [19] days)[51]. The incremental shuttle walk test distances following face-to-face rehabilitation (mean 52 [95% CI 19 to 85] m, p=0.002) and remote rehabilitation (mean 34 [95% CI 1 to 66] m, p=0.047) improved compared to usual care alone, but the interventions did not improve health-related quality of life or self-reported symptoms.

There are notable differences between PHOSP-R and our study. PHOSP-R enrolled individuals who had been hospitalized for COVID-19, the intervention included mixed types of exercise (including aerobic exercise), education and self-management, and the duration of the intervention was 8-weeks, and no information was described on post-exercise malaise[51]. In our trial, the population was larger, most (65%) of the participants were female (45% PHOSP-R), and most (61%) had experienced COVID-19 in the community (Table 1). The intervention involved a personalized resistance exercise intervention, the duration was longer (12-weeks), and the intervention did not increase post-exercise malaise. In PHOSP-R, the shuttle walk distance at baseline in the usual care group was 328 (277-380) m which is greater than the distance achieved by participants in our trial (270 (180, 430) m) reflecting a greater degree of physical limitation in our population.

© 2025 Berry C et al. *JAMA Network Open.*

eTable 1. Schedule of Enrollment, Interventions and Assessments

**Visit**	**1**	**2**	**3**
**Timeline**	**Day 1**	**3 months (+6weeks)**	**Electronic health record linkage**
**Trial Activity**<br><br>**Setting**	**Enrolment**<br><br>**Hospitalized, non-hospitalized COVID-19**	**Outcome evaluation**<br><br>**CRF**	**1 & 10 years**
Screening – Inclusion/Exclusion Criteria including DASI questionnaire	√		
Written Informed Consent	√		
General health status check	√		
Medical History / clinical status[2]	√	√	
Vital signs (heart rate, rhythm, BP, height, weight, waist circumference, oxygen saturation, at baseline and during follow-up)	√	√	
Cardiovascular risk factors, risk score	√	√	
Routine blood samples as per standard of care	√	√	
Spirometry	√	√	
Handgrip strength	√	√	

© 2025 Berry C et al. *JAMA Network Open.*

Short Physical Performance Battery (SPPB)	√	√	
Accelerometer (Glasgow only)	√	√	
PROMS including EQ5D, PHQ, Illness perception (Brief IPQ), Duke Activity Status Index (DASI), International Physical Activity Questionnaire (IPAQ-SF) short-form and Fatigue questionnaires	√	√	
Frailty 1) Fried 5-criteria phenotype, 2) Clinical Frailty Scale	√	√	
Training - Hospital, CRF, on-line, telephone			
Randomization	√		
Training (resistance exercise)	√		
Incremental shuttle walk test (ISWT)	√	√	
Exercise dose (log, level, adherence)	√	√	
Episodes of care (primary, secondary, physiotherapy, rehabilitation)		√	√
Research blood sample		√	
Gluteal Biopsy (optional vascular biology sub-study)		√	
Data collection – clinical endpoints (collected via hospital electronic health record systems) and e-record linkage			√

© 2025 Berry C et al. *JAMA Network Open.*

eTable 2. Number of Participants Randomized Under Each Protocol Version at Each Site

	All	Queen Elizabeth University Hospital, Glasgow	Glasgow Royal Infirmary	Ninewells Hospital, Dundee
N Randomized				
N	233	173	41	19
Protocol				
N ($N_{MISSING}$)	233 (0)	173 (0)	41 (0)	19 (0)
N (%) Version 1.0 and 1.1	104 (44.6%)	88 (50.9%)	14 (34.1%)	2 (10.5%)
N (%) Version 2.0	24 (10.3%)	12 (6.9%)	5 (12.2%)	7 (36.8%)
N (%) Version 3.0 and 4.0	33 (14.2%)	23 (13.3%)	3 (7.3%)	7 (36.8%)
N (%) Version 5.0 onwards	72 (30.9%)	50 (28.9%)	19 (46.3%)	3 (15.8%)

© 2025 Berry C et al. *JAMA Network Open.*

eTable 3. Baseline Characteristics: COVID-19 Treatment

	All	Standard Care	Intervention
N Randomised			
N	233	116	117
Supplemental oxygen			
N ($N_{MISSING}$)	232 (1)	115 (1)	117 (0)
N (%) Yes	53 (22.8%)	24 (20.9%)	29 (24.8%)
Non-invasive respiratory support			
N ($N_{MISSING}$)	232 (1)	115 (1)	117 (0)
N (%) Yes	6 (2.6%)	4 (3.5%)	2 (1.7%)
Invasive Ventilation			
N ($N_{MISSING}$)	232 (1)	115 (1)	117 (0)
N (%) Yes	1 (0.4%)	1 (0.9%)	0 (0.0%)

© 2025 Berry C et al. *JAMA Network Open.*

eTable 4. COVID-19 Reinfection and Vaccination

	All	Standard Care	Intervention
History of COVID-19 reinfections			
N ($N_{MISSING}$)	233 (0)	116 (0)	117 (0)
N (%) Yes	44 (18.9%)	21 (18.1%)	23 (19.7%)
Number of recorded COVID-19 reinfections			
N ($N_{MISSING}$)	44 (0)	21 (0)	23 (0)
Mean (SD)<br>Median (IQR)<br>[Min, Max]	1.4 (0.7)<br>1.0 (1.0, 2.0)<br>[1.0, 4.0]	1.5 (0.6)<br>1.0 (1.0, 2.0)<br>[1.0, 3.0]	1.3 (0.7)<br>1.0 (1.0, 1.0)<br>[1.0, 4.0]
Number of recorded reinfections which resulted in hospitalization			
N ($N_{MISSING}$)	44 (0)	21 (0)	23 (0)
Mean (SD)<br>Median (IQR)<br>[Min, Max]	0.0 (0.0)<br>0.0 (0.0, 0.0)<br>[0.0, 0.0]	0.0 (0.0)<br>0.0 (0.0, 0.0)<br>[0.0, 0.0]	0.0 (0.0)<br>0.0 (0.0, 0.0)<br>[0.0, 0.0]
COVID-19 vaccination - first dose			
N ($N_{MISSING}$)	232 (1)	116 (0)	116 (1)
N (%) Yes	221 (95.3%)	113 (97.4%)	108 (93.1%)
COVID-19 vaccination - second dose			
N ($N_{MISSING}$)	232 (1)	116 (0)	116 (1)
N (%) Yes	216 (93.1%)	112 (96.6%)	104 (89.7%)
COVID-19 vaccination - third dose			
N ($N_{MISSING}$)	232 (1)	116 (0)	116 (1)

© 2025 Berry C et al. *JAMA Network Open.*

eTable 4. COVID-19 Reinfection and Vaccination

	All	Standard Care	Intervention
N (%) Yes	197 (84.9%)	104 (89.7%)	93 (80.2%)
COVID-19 vaccination - fourth dose			
N ($N_{MISSING}$)	232 (1)	116 (0)	116 (1)
N (%) Yes	75 (32.3%)	40 (34.5%)	35 (30.2%)

© 2025 Berry C et al. *JAMA Network Open.*

eTable 5. Primary Outcome: Incremental Shuttle Walk Test Distance (m) at Baseline

	All	Standard Care	Intervention
N Randomised			
N	233	116	117
ISWT distance at baseline (m)			
N ($N_{MISSING}$)	224 (9)	112 (4)	112 (5)
Mean (SD)<br>Median (IQR)<br>[Min, Max]	328 (225)<br>270 (180, 430)<br>[10, 1230]	340 (242)<br>270 (180, 450)<br>[30, 1230]	315 (207)<br>270 (180, 422)<br>[10, 1030]
Reason for stopping at baseline: Tiredness			
N ($N_{MISSING}$)	224 (9)	112 (4)	112 (5)
N (%) Yes	29 (12.9%)	10 (8.9%)	19 (17.0%)
Reason for stopping at baseline: Breathlessness			
N ($N_{MISSING}$)	224 (9)	112 (4)	112 (5)
N (%) Yes	76 (33.9%)	35 (31.2%)	41 (36.6%)
Reason for stopping at baseline: Chest pain			
N ($N_{MISSING}$)	224 (9)	112 (4)	112 (5)
N (%) Yes	3 (1.3%)	1 (0.9%)	2 (1.8%)
Reason for stopping at baseline: Joint pain			
N ($N_{MISSING}$)	224 (9)	112 (4)	112 (5)
N (%) Yes	21 (9.4%)	9 (8.0%)	12 (10.7%)
Reason for stopping at baseline: Other			
N ($N_{MISSING}$)	224 (9)	112 (4)	112 (5)

© 2025 Berry C et al. *JAMA Network Open.*

	All	Standard Care	Intervention
N (%) Yes	169 (75.4%)	83 (74.1%)	86 (76.8%)

© 2025 Berry C et al. *JAMA Network Open.*

eTable 6. Primary Outcome: Incremental Shuttle Walk Test Distance (m) at 3 Months

	All	Standard Care	Intervention
N with follow-up data			
N	195	100	95
ISWT distance at 3m follow-up (m)			
N ($N_{MISSING}$)	193 (2)	99 (1)	94 (1)
Mean (SD)<br>Median (IQR)<br>[Min, Max]	389 (249)<br>340 (200, 520)<br>[40, 1290]	381 (254)<br>340 (195, 465)<br>[60, 1290]	396 (245)<br>350 (200, 528)<br>[40, 1080]
Reason for stopping at 3m follow-up: Tiredness			
N ($N_{MISSING}$)	194 (1)	100 (0)	94 (1)
N (%) Yes	25 (12.9%)	10 (10.0%)	15 (16.0%)
Reason for stopping at 3m follow-up: Breathlessness			
N ($N_{MISSING}$)	194 (1)	100 (0)	94 (1)
N (%) Yes	77 (39.7%)	36 (36.0%)	41 (43.6%)
Reason for stopping at 3m follow-up: Chest pain			
N ($N_{MISSING}$)	194 (1)	100 (0)	94 (1)
N (%) Yes	4 (2.1%)	2 (2.0%)	2 (2.1%)
Reason for stopping at 3m follow-up: Joint pain			
N ($N_{MISSING}$)	194 (1)	100 (0)	94 (1)
N (%) Yes	18 (9.3%)	8 (8.0%)	10 (10.6%)
Reason for stopping at 3m follow-up: Other			
N ($N_{MISSING}$)	194 (1)	100 (0)	94 (1)

© 2025 Berry C et al. *JAMA Network Open.*

	All	Standard Care	Intervention
N (%) Yes	150 (77.3%)	79 (79.0%)	71 (75.5%)

IQR = interquartile range; ISWT = incremental shuttle walk distance; m = months; max = maximum; min = minimum; SD = standard deviation.

© 2025 Berry C et al. *JAMA Network Open.*

eTable 7. Primary Outcome Analysis: Incremental Shuttle Walk Test Distance (m) With Imputed Data

		Data as collected, n=192	Imputed data, n=233
Intervention effect	Estimate	36.46	37.07
	95% CI	(6.58, 66.34)	(6.90, 67.24)
	p-value	p=0.017	p=0.016

The incremental shuttle walk test (ISWT) values at baseline and follow-up were imputed where missing for all randomized participants. Each randomized group was imputed separately. In each case, 100 imputed datasets were derived by predictive mean matching, with age, sex, clinical presentation group, history of COVID-19 pneumonia, handgrip strength, and FVC being used as predictors. The results from running the analysis on each imputed dataset are then pooled. The results presented are intervention effect estimates for ISWT distance at 3m follow-up, adjusted for baseline ISWT distance, clinical presentation, history of COVID-19 pneumonia, age, sex, and site.

eTable 8. Primary Outcome - Causal Effect Analysis at Various Compliance Levels

		Intervention effect	
Analysis description	N (%) compliant	Estimate (95% CI)	p-value
Original primary outcome analysis	-	36.46 (6.58, 66.34)	p=0.017
CACE analysis, compliance defined as adherence ≥ 10%	85 (92.4%)	41.39 (9.03, 73.75)	p=0.012
CACE analysis, compliance defined as adherence ≥ 20%	82 (89.1%)	42.91 (9.46, 76.35)	p=0.012
CACE analysis, compliance defined as adherence ≥ 30%	78 (84.8%)	45.13 (10.16, 80.10)	p=0.011
CACE analysis, compliance defined as adherence ≥ 40%	72 (78.3%)	48.11 (10.79, 85.43)	p=0.012
CACE analysis, compliance defined as adherence ≥ 50%	67 (72.8%)	51.93 (11.50, 92.36)	p=0.012
CACE analysis, compliance defined as adherence ≥ 60%	55 (59.8%)	62.87 (13.93, 111.81)	p=0.012
CACE analysis, compliance defined as adherence ≥ 70%	47 (51.1%)	73.60 (15.96, 131.25)	p=0.012
CACE analysis, compliance defined as adherence ≥ 80%	39 (42.4%)	88.10 (19.26, 156.93)	p=0.012
CACE analysis, compliance defined as adherence ≥ 90%	31 (33.7%)	111.40 (22.89, 199.91)	p=0.014

The original primary outcome analysis does not take adherence with the intervention into account. The CACE models below adjust for compliance at set cutoffs, note that adherence with intervention was self-reported by the participants.

© 2025 Berry C et al. *JAMA Network Open.*

eTable 9. Primary Outcome: Incremental Shuttle Walk Test in subjects With Good Exercise Adherence (>70%, Intervention Group Only) by Clinical Presentation Group

	All	Group A	Group B	Group C
N Randomised				
N	47	30	14	3
Baseline ISWT distance, m				
N ($N_{MISSING}$)	47 (0)	30 (0)	14 (0)	3 (0)
Mean (SD)<br>Median (IQR)<br>[Min, Max]	336.60 (232.30)<br>330.00 (180.00, 430.00)<br>[10.00, 1030.00]	379.00 (252.58)<br>335.00 (200.00, 430.00)<br>[10.00, 1030.00]	247.14 (183.28)<br>180.00 (102.50, 357.50)<br>[30.00, 580.00]	330.00 (121.24)<br>350.00 (275.00, 395.00)<br>[200.00, 440.00]
N with follow-up data				
N	47	30	14	3
3m follow-up ISWT distance, m				
N ($N_{MISSING}$)	47 (0)	30 (0)	14 (0)	3 (0)
Mean (SD)<br>Median (IQR)<br>[Min, Max]	440.85 (282.76)<br>430.00 (195.00, 585.00)<br>[40.00, 1080.00]	488.00 (279.62)<br>440.00 (262.50, 617.50)<br>[90.00, 1080.00]	322.14 (286.63)<br>260.00 (92.50, 452.50)<br>[40.00, 1040.00]	523.33 (170.10)<br>590.00 (460.00, 620.00)<br>[330.00, 650.00]

© 2025 Berry C et al. *JAMA Network Open.*

eTable 9. Primary Outcome: Incremental Shuttle Walk Test in subjects With Good Exercise Adherence (>70%, Intervention Group Only) by Clinical Presentation Group

	All	Group A	Group B	Group C
Change in ISWT distance, m				
N ($N_{MISSING}$)	47 (0)	30 (0)	14 (0)	3 (0)
Mean (SD)<br>Median (IQR)<br>[Min, Max]	104.26 (134.47)<br>100.00 (15.00, 165.00)<br>[-170.00, 460.00]	109.00 (139.69)<br>100.00 (12.50, 225.00)<br>[-170.00, 390.00]	75.00 (127.75)<br>50.00 (5.00, 105.00)<br>[-70.00, 460.00]	193.33 (92.92)<br>150.00 (140.00, 225.00)<br>[130.00, 300.00]

© 2025 Berry C et al. *JAMA Network Open.*

eTable 10. Secondary Outcome Analysis: Spirometry - Peak Expiratory Flow Rate (L/m)

	All	Standard Care	Intervention
N Randomised			
N	233	116	117
Baseline Peak expiratory flow rate (L/m)			
N ($N_{MISSING}$)	222 (11)	111 (5)	111 (6)
Mean (SD)	404.64 (135.54)	394.83 (139.92)	414.46 (130.92)
Median (IQR)	398.50 (312.25, 491.75)	392.00 (303.50, 474.00)	400.00 (330.00, 511.00)
[Min, Max]	[80.00, 758.00]	[80.00, 758.00]	[156.00, 737.00]
N with follow-up data			
N	195	100	95
3m follow-up Peak expiratory flow rate (L/m)			
N ($N_{MISSING}$)	189 (6)	98 (2)	91 (4)
Mean (SD)	413.46 (140.14)	409.02 (142.98)	418.23 (137.64)
Median (IQR)	407.00 (307.00, 497.00)	396.50 (304.75, 492.50)	422.00 (314.00, 501.50)
[Min, Max]	[104.00, 794.00]	[111.00, 794.00]	[104.00, 791.00]
Change in Peak expiratory flow rate (L/m)			
N ($N_{MISSING}$)	187 (8)	96 (4)	91 (4)
Mean (SD)	-0.80 (86.64)	3.01 (86.61)	-4.81 (86.97)
Median (IQR)	-5.00 (-46.00, 35.50)	3.50 (-41.25, 35.25)	-8.00 (-50.00, 34.00)
[Min, Max]	[-197.00, 300.00]	[-177.00, 300.00]	[-197.00, 242.00]

Linear regression intervention effect estimate, adjusted for baseline peak expiratory flow rate, clinical presentation, history of COVID-19 pneumonia, age, sex, and site.

Estimate		-0.80
(95% CI)		(-23.04, 21.45)
p-value		p=0.944

© 2025 Berry C et al. *JAMA Network Open.*

eTable 11. Secondary Outcome Analysis: Spirometry - Forced Vital Capacity (L)

	All	Standard Care	Intervention
N Randomised			
N	233	116	117
Baseline forced vital capacity (L)			
N ($N_{MISSING}$)	222 (11)	111 (5)	111 (6)
Mean (SD)<br>Median (IQR)<br>[Min, Max]	3.54 (0.96)<br>3.50 (2.90, 4.07)<br>[1.40, 6.80]	3.50 (1.00)<br>3.40 (2.80, 4.00)<br>[1.40, 6.80]	3.58 (0.92)<br>3.50 (2.90, 4.15)<br>[1.60, 6.00]
N with follow-up data			
N	195	100	95
3m follow-up forced vital capacity (L)			
N ($N_{MISSING}$)	189 (6)	98 (2)	91 (4)
Mean (SD)<br>Median (IQR)<br>[Min, Max]	3.61 (0.97)<br>3.60 (2.90, 4.20)<br>[1.40, 6.80]	3.58 (1.01)<br>3.50 (2.90, 4.27)<br>[1.40, 6.80]	3.65 (0.93)<br>3.60 (3.10, 4.15)<br>[1.50, 5.90]
Change in forced vital capacity (L)			
N ($N_{MISSING}$)	187 (8)	96 (4)	91 (4)
Mean (SD)<br>Median (IQR)<br>[Min, Max]	0.05 (0.40)<br>0.00 (-0.10, 0.20)<br>[-1.90, 1.40]	0.04 (0.39)<br>0.00 (-0.10, 0.20)<br>[-1.90, 1.10]	0.06 (0.42)<br>0.00 (-0.10, 0.20)<br>[-1.30, 1.40]
Linear regression intervention effect estimate, adjusted for baseline forced vital capacity, clinical presentation, history of COVID-19 pneumonia, age, sex, and site.			
Estimate<br>(95% CI)<br>p-value		0.02<br>(-0.08, 0.13)<br>p=0.699	

© 2025 Berry C et al. *JAMA Network Open.*

eTable 12. Secondary Outcome Analysis: Spirometry - Forced Expiratory Volume in One Second (L)

	All	Standard Care	Intervention
N Randomised			
N	233	116	117
Baseline forced expiratory volume in one second (L)			
N ($N_{MISSING}$)	222 (11)	111 (5)	111 (6)
Mean (SD)<br>Median (IQR)<br>[Min, Max]	2.67 (0.86)<br>2.60 (2.10, 3.20)<br>[0.70, 5.70]	2.65 (0.91)<br>2.60 (2.05, 3.25)<br>[0.70, 5.70]	2.69 (0.80)<br>2.70 (2.20, 3.20)<br>[0.70, 4.90]
N with follow-up data			
N	195	100	95
3m follow-up forced expiratory volume in one second (L)			
N ($N_{MISSING}$)	189 (6)	98 (2)	91 (4)
Mean (SD)<br>Median (IQR)<br>[Min, Max]	2.76 (0.87)<br>2.70 (2.20, 3.30)<br>[0.40, 5.60]	2.76 (0.88)<br>2.70 (2.20, 3.27)<br>[0.80, 5.60]	2.77 (0.86)<br>2.80 (2.25, 3.30)<br>[0.40, 5.20]
Change in forced expiratory volume in one second (L)			
N ($N_{MISSING}$)	187 (8)	96 (4)	91 (4)
Mean (SD)<br>Median (IQR)<br>[Min, Max]	0.06 (0.51)<br>0.00 (-0.10, 0.10)<br>[-2.30, 2.40]	0.05 (0.39)<br>0.00 (-0.10, 0.10)<br>[-0.90, 2.00]	0.06 (0.61)<br>0.00 (-0.10, 0.15)<br>[-2.30, 2.40]
Linear regression intervention effect estimate, adjusted for baseline forced expiratory volume in one second, clinical presentation, history of COVID-19 pneumonia, age, sex, and site.			
Estimate<br>(95% CI)<br>p-value		0.00<br>(-0.13, 0.14)<br>p=0.950	

© 2025 Berry C et al. *JAMA Network Open.*

eTable 13. Secondary Outcome Analysis: Spirometry - FEV1/FVC Ratio

	All	Standard Care	Intervention
N Randomised			
N	233	116	117
Baseline FEV1/FVC ratio			
N ($N_{MISSING}$)	222 (11)	111 (5)	111 (6)
Mean (SD)<br>Median (IQR)<br>[Min, Max]	0.75 (0.12)<br>0.78 (0.73, 0.82)<br>[0.17, 1.13]	0.75 (0.13)<br>0.78 (0.73, 0.83)<br>[0.17, 1.13]	0.75 (0.12)<br>0.78 (0.73, 0.82)<br>[0.17, 0.92]
N with follow-up data			
N	195	100	95
3m follow-up FEV1/FVC ratio			
N ($N_{MISSING}$)	189 (6)	98 (2)	91 (4)
Mean (SD)<br>Median (IQR)<br>[Min, Max]	0.76 (0.11)<br>0.78 (0.74, 0.82)<br>[0.12, 0.93]	0.77 (0.10)<br>0.79 (0.75, 0.82)<br>[0.27, 0.92]	0.76 (0.13)<br>0.78 (0.73, 0.82)<br>[0.12, 0.93]
Change in FEV1/FVC ratio			
N ($N_{MISSING}$)	187 (8)	96 (4)	91 (4)
Mean (SD)<br>Median (IQR)<br>[Min, Max]	0.00 (0.13)<br>0.00 (-0.03, 0.02)<br>[-0.70, 0.60]	0.00 (0.10)<br>0.00 (-0.02, 0.02)<br>[-0.30, 0.55]	0.00 (0.15)<br>0.00 (-0.03, 0.02)<br>[-0.70, 0.60]

Linear regression intervention effect estimate, adjusted for baseline FEV1/FVC ratio, clinical presentation, history of COVID-19 pneumonia, age, sex, and site.

Estimate<br>(95% CI)<br>p-value		-0.01<br>(-0.04, 0.02)<br>p=0.543	

© 2025 Berry C et al. *JAMA Network Open.*

eTable 14. Secondary Outcome Analysis: Handgrip Strength (kg)

	All	Standard Care	Intervention
N Randomised			
N	233	116	117
Baseline handgrip strength (kg)			
N ($N_{MISSING}$)	225 (8)	113 (3)	112 (5)
Mean (SD)<br>Median (IQR)<br>[Min, Max]	28.9 (12.0)<br>27.1 (20.0, 36.7)<br>[3.3, 69.7]	29.6 (12.2)<br>28.1 (20.2, 37.0)<br>[3.3, 69.7]	28.2 (11.9)<br>26.2 (20.0, 35.0)<br>[4.7, 65.3]
N with follow-up data			
N	195	100	95
3m follow-up handgrip strength (kg)			
N ($N_{MISSING}$)	194 (1)	99 (1)	95 (0)
Mean (SD)<br>Median (IQR)<br>[Min, Max]	30.6 (12.3)<br>29.5 (22.0, 37.8)<br>[5.7, 71.2]	29.8 (12.2)<br>29.6 (20.7, 34.8)<br>[5.7, 67.0]	31.4 (12.4)<br>29.3 (23.4, 40.4)<br>[6.3, 71.2]
Change in handgrip strength (kg)			
N ($N_{MISSING}$)	193 (2)	99 (1)	94 (1)
Mean (SD)<br>Median (IQR)<br>[Min, Max]	1.4 (6.4)<br>1.7 (-1.9, 4.6)<br>[-30.4, 26.7]	0.0 (6.9)<br>0.9 (-3.0, 3.5)<br>[-30.4, 19.6]	2.9 (5.6)<br>2.5 (-0.8, 5.8)<br>[-9.6, 26.7]
Linear regression intervention effect estimate, adjusted for baseline handgrip strength, clinical presentation, history of COVID-19 pneumonia, age, sex, and site.			
Estimate<br>(95% CI)<br>p-value		2.58<br>(0.92, 4.24)<br>p=0.002	

eTable 15. Secondary Outcome Analysis: Short Physical Performance Battery Score Category

	All	Standard Care	Intervention
N Randomised			
N	233	116	117
Baseline SPPB score category			
N ($N_{MISSING}$)	212 (21)	107 (9)	105 (12)
N (%) Poor	3 (1.4%)	2 (1.9%)	1 (1.0%)
N (%) Moderate	90 (42.5%)	45 (42.1%)	45 (42.9%)
N (%) Good	119 (56.1%)	60 (56.1%)	59 (56.2%)
N with follow-up data			
N	195	100	95
3m follow-up SPPB score category			
N ($N_{MISSING}$)	181 (14)	89 (11)	92 (3)
N (%) Poor	1 (0.6%)	0 (0.0%)	1 (1.1%)
N (%) Moderate	52 (28.7%)	27 (30.3%)	25 (27.2%)
N (%) Good	128 (70.7%)	62 (69.7%)	66 (71.7%)
Change in SPPB score category			
N ($N_{MISSING}$)	174 (21)	86 (14)	88 (7)
N (%) Improvement	38 (21.8%)	13 (15.1%)	25 (28.4%)
N (%) No change	124 (71.3%)	69 (80.2%)	55 (62.5%)
N (%) Deterioration	12 (6.9%)	4 (4.7%)	8 (9.1%)
Ordinal regression intervention effect odds ratio, adjusted for, baseline SPPB score category, clinical presentation, history of COVID-19 pneumonia, age, sex, and site.			
OR<br>(95% CI)<br>p-value		1.7<br>(0.7, 3.8)<br>p=0.227	
Test of proportional odds assumption (for treatment group)		p=0.187	

© 2025 Berry C et al. *JAMA Network Open.*

eTable 16. Secondary Outcome Analysis: EQ-5D-5L Utility Score (UK Crosswalk Value Set)

	All	Standard care	Intervention
N Randomised			
N	233	116	117
Baseline EQ-5D-5L utility score			
N ($N_{MISSING}$)	226 (7)	113 (3)	113 (4)
Mean (SD)<br>Median (IQR)<br>[Min, Max]	0.64 (0.22)<br>0.68 (0.56, 0.77)<br>[-0.11, 1.00]	0.66 (0.21)<br>0.69 (0.58, 0.80)<br>[-0.11, 1.00]	0.63 (0.24)<br>0.67 (0.53, 0.76)<br>[-0.09, 1.00]
N with follow-up data			
N	195	100	95
3m follow-up EQ-5D-5L utility score			
N ($N_{MISSING}$)	195 (0)	100 (0)	95 (0)
Mean (SD)<br>Median (IQR)<br>[Min, Max]	0.67 (0.25)<br>0.71 (0.55, 0.84)<br>[-0.09, 1.00]	0.65 (0.25)<br>0.69 (0.56, 0.80)<br>[-0.08, 1.00]	0.68 (0.25)<br>0.72 (0.55, 0.88)<br>[-0.09, 1.00]
Change in EQ-5D-5L utility score			
N ($N_{MISSING}$)	195 (0)	100 (0)	95 (0)
Mean (SD)<br>Median (IQR)<br>[Min, Max]	0.02 (0.19)<br>0.00 (-0.05, 0.14)<br>[-0.81, 0.68]	-0.01 (0.19)<br>0.00 (-0.07, 0.07)<br>[-0.81, 0.55]	0.06 (0.19)<br>0.01 (-0.02, 0.21)<br>[-0.42, 0.68]
Linear regression intervention effect estimate, adjusted for baseline EQ-5D-5L utility score, clinical presentation, history of COVID-19 pneumonia, age, sex, and site.			
Estimate<br>(95% CI)<br>p-value		0.062<br>(0.011, 0.113)<br>p=0.018	

© 2025 Berry C et al. *JAMA Network Open.*

eTable 17. Secondary Outcome Analysis: EQ-5D-5L Visual Analogue Scale

	All	Standard Care	Intervention
N Randomised			
N	233	116	117
Baseline EQ-5D-5L VAS			
N ($N_{MISSING}$)	226 (7)	113 (3)	113 (4)
Mean (SD)<br>Median (IQR)<br>[Min, Max]	63.1 (18.0)<br>65.0 (50.0, 75.0)<br>[10.0, 100.0]	64.7 (16.9)<br>70.0 (52.0, 75.0)<br>[10.0, 95.0]	61.4 (18.9)<br>65.0 (50.0, 75.0)<br>[10.0, 100.0]
N with follow-up data			
N	195	100	95
3m follow-up EQ-5D-5L VAS			
N ($N_{MISSING}$)	195 (0)	100 (0)	95 (0)
Mean (SD)<br>Median (IQR)<br>[Min, Max]	68.2 (19.5)<br>70.0 (55.0, 85.0)<br>[20.0, 100.0]	67.8 (19.4)<br>70.0 (55.0, 83.0)<br>[20.0, 100.0]	68.7 (19.7)<br>70.0 (55.0, 85.0)<br>[25.0, 100.0]
Change in EQ-5D-5L VAS			
N ($N_{MISSING}$)	195 (0)	100 (0)	95 (0)
Mean (SD)<br>Median (IQR)<br>[Min, Max]	4.3 (16.7)<br>5.0 (-2.5, 14.5)<br>[-50.0, 60.0]	2.0 (15.2)<br>0.0 (-3.5, 10.0)<br>[-35.0, 45.0]	6.8 (17.9)<br>5.0 (0.0, 15.0)<br>[-50.0, 60.0]
Linear regression intervention effect estimate, adjusted for baseline EQ-5D-5L VAS, clinical presentation, history of COVID-19 pneumonia, age, sex, and site.			
Estimate<br>(95% CI)<br>p-value		3.6<br>(-0.7, 8.0)<br>p=0.101	

© 2025 Berry C et al. *JAMA Network Open.*

eTable 18. Secondary Outcome Analysis: Patient Health Questionnaire Category

	All	Standard Care	Intervention
N Randomised			
N	233	116	117
Baseline PHQ score category			
N ($N_{MISSING}$)	223 (10)	111 (5)	112 (5)
N (%) Normal	80 (35.9%)	46 (41.4%)	34 (30.4%)
N (%) Mild	64 (28.7%)	24 (21.6%)	40 (35.7%)
N (%) Moderate	40 (17.9%)	23 (20.7%)	17 (15.2%)
N (%) Severe	39 (17.5%)	18 (16.2%)	21 (18.8%)
N with follow-up data			
N	195	100	95
3m follow-up PHQ score category			
N ($N_{MISSING}$)	195 (0)	100 (0)	95 (0)
N (%) Normal	92 (47.2%)	42 (42.0%)	50 (52.6%)
N (%) Mild	42 (21.5%)	23 (23.0%)	19 (20.0%)
N (%) Moderate	30 (15.4%)	18 (18.0%)	12 (12.6%)
N (%) Severe	31 (15.9%)	17 (17.0%)	14 (14.7%)
Change in PHQ score category			
N ($N_{MISSING}$)	193 (2)	99 (1)	94 (1)
N (%) Improvement	52 (26.9%)	18 (18.2%)	34 (36.2%)
N (%) No change	107 (55.4%)	61 (61.6%)	46 (48.9%)
N (%) Deterioration	34 (17.6%)	20 (20.2%)	14 (14.9%)

Ordinal regression intervention effect odds ratio, adjusted for, baseline PHQ score category, clinical presentation, history of COVID-19 pneumonia, age, sex, and site.

OR		0.5
(95% CI)		(0.2, 0.8)
p-value		p=0.013

© 2025 Berry C et al. *JAMA Network Open.*

	All	Standard Care	Intervention
Test of proportional odds assumption (for treatment group)		p=0.181	

© 2025 Berry C et al. *JAMA Network Open.*

eTable 19. Secondary Outcome Analysis: Brief Illness Perception Questionnaire Score

	All	Standard Care	Intervention
N Randomised			
N	233	116	117
Baseline brief IPQ score			
N ($N_{MISSING}$)	225 (8)	113 (3)	112 (5)
Mean (SD)<br>Median (IQR)<br>[Min, Max]	45.82 (14.26)<br>47.00 (37.00, 55.00)<br>[0.00, 76.00]	44.87 (14.93)<br>46.00 (36.00, 56.00)<br>[0.00, 76.00]	46.78 (13.54)<br>48.00 (39.00, 55.00)<br>[10.00, 75.00]
N with follow-up data			
N	195	100	95
3m follow-up brief IPQ score			
N ($N_{MISSING}$)	195 (0)	100 (0)	95 (0)
Mean (SD)<br>Median (IQR)<br>[Min, Max]	40.41 (17.42)<br>42.00 (29.50, 53.00)<br>[0.00, 74.00]	40.68 (17.09)<br>43.00 (27.75, 53.00)<br>[0.00, 74.00]	40.14 (17.84)<br>42.00 (30.00, 53.50)<br>[0.00, 73.00]
Change in brief IPQ score			
N ($N_{MISSING}$)	194 (1)	100 (0)	94 (1)
Mean (SD)<br>Median (IQR)<br>[Min, Max]	-4.86 (13.43)<br>-4.00 (-12.00, 3.00)<br>[-46.00, 50.00]	-2.98 (11.66)<br>-2.50 (-9.25, 3.25)<br>[-30.00, 50.00]	-6.86 (14.88)<br>-6.00 (-13.75, 1.00)<br>[-46.00, 39.00]
Linear regression intervention effect estimate, adjusted for baseline brief IPQ score, clinical presentation, history of COVID-19 pneumonia, age, sex, and site.			
Estimate<br>(95% CI)<br>p-value		-3.52<br>(-7.25, 0.22)<br>p=0.065	

© 2025 Berry C et al. *JAMA Network Open.*

eTable 20. Secondary Outcome Analysis: Duke Activity Status Index Score

	All	Standard Care	Intervention
N Randomised			
N	233	116	117
Baseline DASI score			
N ($N_{MISSING}$)	222 (11)	110 (6)	112 (5)
Mean (SD)<br>Median (IQR)<br>[Min, Max]	27.78 (15.74)<br>23.82 (15.45, 42.08)<br>[4.45, 58.20]	28.20 (15.34)<br>23.82 (15.45, 39.95)<br>[4.50, 58.20]	27.38 (16.18)<br>22.85 (15.39, 42.70)<br>[4.45, 58.20]
N with follow-up data			
N	195	100	95
3m follow-up DASI score			
N ($N_{MISSING}$)	194 (1)	100 (0)	94 (1)
Mean (SD)<br>Median (IQR)<br>[Min, Max]	32.26 (17.78)<br>28.70 (15.45, 50.70)<br>[0.00, 58.20]	32.59 (17.73)<br>28.70 (15.82, 50.70)<br>[0.00, 58.20]	31.91 (17.92)<br>27.82 (15.45, 50.70)<br>[4.45, 58.20]
Change in DASI score			
N ($N_{MISSING}$)	190 (5)	97 (3)	93 (2)
Mean (SD)<br>Median (IQR)<br>[Min, Max]	4.56 (10.59)<br>3.35 (0.00, 9.88)<br>[-23.75, 39.50]	4.15 (9.67)<br>3.50 (-2.70, 9.00)<br>[-18.20, 39.50]	4.98 (11.52)<br>2.75 (0.00, 10.70)<br>[-23.75, 35.50]
Linear regression intervention effect estimate, adjusted for baseline DASI score, clinical presentation, history of COVID-19 pneumonia, age, sex, and site.			
Estimate<br>(95% CI)<br>p-value		0.92<br>(-2.05, 3.89)<br>p=0.543	

© 2025 Berry C et al. *JAMA Network Open.*

eTable 21. Secondary Outcome Analysis: Duke Activity Status Index Predicted VO2max

	All	Standard Care	Intervention
N Randomised			
N	233	116	117
Baseline DASI predicted VO2max			
N ($N_{MISSING}$)	222 (11)	110 (6)	112 (5)
Mean (SD)<br>Median (IQR)<br>[Min, Max]	21.55 (6.77)<br>19.84 (16.24, 27.69)<br>[11.51, 34.63]	21.73 (6.60)<br>19.84 (16.24, 26.78)<br>[11.54, 34.63]	21.37 (6.96)<br>19.43 (16.22, 27.96)<br>[11.51, 34.63]
N with follow-up data			
N	195	100	95
3m follow-up DASI predicted VO2max			
N ($N_{MISSING}$)	194 (1)	100 (0)	94 (1)
Mean (SD)<br>Median (IQR)<br>[Min, Max]	23.47 (7.64)<br>21.94 (16.24, 31.40)<br>[9.60, 34.63]	23.62 (7.62)<br>21.94 (16.40, 31.40)<br>[9.60, 34.63]	23.32 (7.71)<br>21.56 (16.24, 31.40)<br>[11.51, 34.63]
Change in DASI predicted VO2max			
N ($N_{MISSING}$)	190 (5)	97 (3)	93 (2)
Mean (SD)<br>Median (IQR)<br>[Min, Max]	1.96 (4.55)<br>1.44 (0.00, 4.25)<br>[-10.21, 16.98]	1.79 (4.16)<br>1.50 (-1.16, 3.87)<br>[-7.83, 16.98]	2.14 (4.95)<br>1.18 (0.00, 4.60)<br>[-10.21, 15.27]
Linear regression intervention effect estimate, adjusted for baseline DASI predicted VO2max, clinical presentation, history of COVID-19 pneumonia, age, sex, and site.			
Estimate<br>(95% CI)<br>p-value		0.40<br>(-0.88, 1.67)<br>p=0.543	

© 2025 Berry C et al. *JAMA Network Open.*

eTable 22. Secondary Outcome Analysis: International Physical Activity Questionnaire (Short Form)

	All	Standard Care	Intervention
N Randomised			
N	233	116	117
Baseline IPAQ-SF activity level			
N ($N_{MISSING}$)	226 (7)	113 (3)	113 (4)
N (%) Low<br>N (%) Moderate<br>N (%) High	95 (42.0%)<br>90 (39.8%)<br>41 (18.1%)	49 (43.4%)<br>42 (37.2%)<br>22 (19.5%)	46 (40.7%)<br>48 (42.5%)<br>19 (16.8%)
N with follow-up data			
N	195	100	95
3m follow-up IPAQ-SF activity level			
N ($N_{MISSING}$)	195 (0)	100 (0)	95 (0)
N (%) Low<br>N (%) Moderate<br>N (%) High	77 (39.5%)<br>70 (35.9%)<br>48 (24.6%)	42 (42.0%)<br>32 (32.0%)<br>26 (26.0%)	35 (36.8%)<br>38 (40.0%)<br>22 (23.2%)
Change in IPAQ-SF activity level			
N ($N_{MISSING}$)	195 (0)	100 (0)	95 (0)
N (%) Improvement<br>N (%) No change<br>N (%) Deterioration	49 (25.1%)<br>110 (56.4%)<br>36 (18.5%)	22 (22.0%)<br>61 (61.0%)<br>17 (17.0%)	27 (28.4%)<br>49 (51.6%)<br>19 (20.0%)
Ordinal regression intervention effect odds ratio, adjusted for, baseline IPAQ-SF activity level, clinical presentation, history of COVID-19 pneumonia, age, sex, and site.			
OR<br>(95% CI)<br>p-value		1.1<br>(0.7, 2.0)<br>p=0.651	

	All	Standard Care	Intervention
Test of proportional odds assumption (for treatment group)		p=0.464	

eTable 23. Secondary Outcome Analysis: Fatigue Severity Score

	All	Standard Care	Intervention
N Randomised			
N	233	116	117
Baseline fatigue severity score			
N ($N_{MISSING}$)	225 (8)	113 (3)	112 (5)
Mean (SD)<br>Median (IQR)<br>[Min, Max]	49 (14)<br>52 (41, 61)<br>[9, 63]	47 (15)<br>51 (37, 60)<br>[9, 63]	50 (14)<br>54 (45, 62)<br>[9, 63]
N with follow-up data			
N	195	100	95
3m follow-up fatigue severity score			
N ($N_{MISSING}$)	195 (0)	100 (0)	95 (0)
Mean (SD)<br>Median (IQR)<br>[Min, Max]	45 (15)<br>48 (34, 59)<br>[9, 63]	45 (15)<br>48 (34, 59)<br>[9, 63]	45 (15)<br>48 (34, 58)<br>[9, 63]
Change in fatigue severity score			
N ($N_{MISSING}$)	194 (1)	100 (0)	94 (1)
Mean (SD)<br>Median (IQR)<br>[Min, Max]	-3 (15)<br>-2 (-10, 2)<br>[-54, 50]	-1 (14)<br>0 (-7, 3)<br>[-51, 50]	-6 (16)<br>-4 (-13, 1)<br>[-54, 42]

Linear regression intervention effect estimate, adjusted for baseline fatigue severity score, clinical presentation, history of COVID-19 pneumonia, age, sex, and site.

© 2025 Berry C et al. *JAMA Network Open.*

	All	Standard Care	Intervention
Estimate<br>(95% CI)<br>p-value		-2.60<br>(-6.38, 1.18)<br>p=0.178	

© 2025 Berry C et al. *JAMA Network Open.*

eTable 24. Secondary Outcome Analysis: MRC Dyspnea Score

	All	Standard Care	Intervention
N Randomised			
N	233	116	117
Baseline MRC dyspnea score			
N ($N_{MISSING}$)	230 (3)	115 (1)	115 (2)
N (%) Not troubled by breathlessness except on strenuous exercise	35 (15.2%)	18 (15.7%)	17 (14.8%)
N (%) Short of breath when hurrying on a level or when walking up a slight hill	91 (39.6%)	47 (40.9%)	44 (38.3%)
N (%) Walks slower than most people on the level, stops after a mile or so, or stops after 15 minutes walking at own pace	77 (33.5%)	38 (33.0%)	39 (33.9%)
N (%) Stops for breath after walking 100 yards, or after a few minutes on level ground	23 (10.0%)	11 (9.6%)	12 (10.4%)
N (%) Too breathless to leave the house, or breathless when dressing/undressing	4 (1.7%)	1 (0.9%)	3 (2.6%)
N with follow-up data			
N	195	100	95
3m follow-up MRC dyspnea score			
N ($N_{MISSING}$)	195 (0)	100 (0)	95 (0)
N (%) Not troubled by breathlessness except on strenuous exercise	50 (25.6%)	25 (25.0%)	25 (26.3%)
N (%) Short of breath when hurrying on a level or when walking up a slight hill	73 (37.4%)	38 (38.0%)	35 (36.8%)
N (%) Walks slower than most people on the level, stops after a mile or so, or stops after 15 minutes walking at own pace	49 (25.1%)	27 (27.0%)	22 (23.2%)
N (%) Stops for breath after walking 100 yards, or after a few minutes on level ground	20 (10.3%)	9 (9.0%)	11 (11.6%)
N (%) Too breathless to leave the house, or breathless when dressing/undressing	3 (1.5%)	1 (1.0%)	2 (2.1%)
Change in MRC dyspnea score			
N ($N_{MISSING}$)	195 (0)	100 (0)	95 (0)

© 2025 Berry C et al. *JAMA Network Open.*

	All	Standard Care	Intervention
N (%) Improvement	59 (30.3%)	28 (28.0%)	31 (32.6%)
N (%) No change	107 (54.9%)	57 (57.0%)	50 (52.6%)
N (%) Deterioration	29 (14.9%)	15 (15.0%)	14 (14.7%)
Ordinal regression intervention effect odds ratio, adjusted for, baseline MRC dyspnea score, clinical presentation, history of COVID-19 pneumonia, age, sex, and site.			
OR<br>(95% CI)<br>p-value		0.9<br>(0.5, 1.5)<br>p=0.639	
Test of proportional odds assumption (for treatment group)		p=0.780	

© 2025 Berry C et al. *JAMA Network Open.*

eTable 25. Secondary Outcome Analysis: Fried Frailty (Sum of Phenotypes)

	All	Standard Care	Intervention
N Randomised			
N	233	116	117
Baseline Fried Frailty			
N ($N_{MISSING}$)	226 (7)	113 (3)	113 (4)
N (%) Not Frail	9 (4.0%)	5 (4.4%)	4 (3.5%)
N (%) Pre-frail	161 (71.2%)	77 (68.1%)	84 (74.3%)
N (%) Frail	56 (24.8%)	31 (27.4%)	25 (22.1%)
N with follow-up data			
N	195	100	95
3m follow-up Fried Frailty			
N ($N_{MISSING}$)	195 (0)	100 (0)	95 (0)
N (%) Not Frail	26 (13.3%)	11 (11.0%)	15 (15.8%)
N (%) Pre-frail	141 (72.3%)	75 (75.0%)	66 (69.5%)
N (%) Frail	28 (14.4%)	14 (14.0%)	14 (14.7%)
Change in Fried Frailty			
N ($N_{MISSING}$)	195 (0)	100 (0)	95 (0)
N (%) Improvement	56 (28.7%)	28 (28.0%)	28 (29.5%)
N (%) No change	123 (63.1%)	65 (65.0%)	58 (61.1%)
N (%) Deterioration	16 (8.2%)	7 (7.0%)	9 (9.5%)
Ordinal regression intervention effect odds ratio, adjusted for, baseline Fried frailty, clinical presentation, history of COVID-19 pneumonia, age, sex, and site.			
OR<br>(95% CI)<br>p-value		0.8<br>(0.4, 1.5)<br>p=0.407	
Test of proportional odds assumption (for treatment group)		p=0.274	

© 2025 Berry C et al. *JAMA Network Open.*

Frequency and severity questions, over the past 6 months. Frequency: 0=none of the time, 1=a little of the time, 2=about half the time, 3=most of the time, 4=all the time. Severity: 0=symptom not present, 1=mild, 2=moderate, 3=severe, 4=very severe.

eTable 26. Post-Exertional Malaise at 3-Months, Part 1 (DePaul Symptom Questionnaire, Short Form)

	All	Standard Care	Intervention
N Randomised			
N	195	100	95
Dead, heavy feeling after starting to exercise: frequency			
N ($N_{MISSING}$)	99 (96)	51 (49)	48 (47)
Mean (SD)	2.2 (1.4)	2.3 (1.4)	2.2 (1.3)
Dead, heavy feeling after starting to exercise: severity			
N ($N_{MISSING}$)	99 (96)	51 (49)	48 (47)
Mean (SD)	2.1 (1.2)	2.1 (1.3)	2.0 (1.2)
Next day soreness or fatigue after non-strenuous, everyday activities: frequency			
N ($N_{MISSING}$)	99 (96)	51 (49)	48 (47)
Mean (SD)	2.3 (1.3)	2.4 (1.3)	2.3 (1.2)
Next day soreness or fatigue after non-strenuous, everyday activities: severity			
N ($N_{MISSING}$)	99 (96)	51 (49)	48 (47)
Mean (SD)	2.1 (1.2)	2.2 (1.3)	2.1 (1.1)
Mentally tired after the slightest effort: frequency			
N ($N_{MISSING}$)	99 (96)	51 (49)	48 (47)
Mean (SD)	2.0 (1.4)	1.9 (1.5)	2.0 (1.3)
Mentally tired after the slightest effort: severity			
N ($N_{MISSING}$)	99 (96)	51 (49)	48 (47)

© 2025 Berry C et al. *JAMA Network Open.*

	All	Standard Care	Intervention
Mean (SD)	1.9 (1.3)	1.9 (1.4)	2.0 (1.2)
Minimum exercise makes you physically tired: frequency			
N ($N_{MISSING}$)	99 (96)	51 (49)	48 (47)
Mean (SD)	2.2 (1.3)	2.3 (1.4)	2.2 (1.2)
Minimum exercise makes you physically tired: severity			
N ($N_{MISSING}$)	99 (96)	51 (49)	48 (47)
Mean (SD)	2.0 (1.2)	2.1 (1.3)	2.0 (1.1)
Physically drained or sick after mild activity: frequency			
N ($N_{MISSING}$)	99 (96)	51 (49)	48 (47)
Mean (SD)	1.8 (1.4)	1.8 (1.5)	1.7 (1.3)
Physically drained or sick after mild activity: severity			
N ($N_{MISSING}$)	99 (96)	51 (49)	48 (47)
Mean (SD)	1.8 (1.3)	1.8 (1.3)	1.7 (1.3)

Frequency and severity questions, over the past 6 months. Frequency: 0=none of the time, 1=a little of the time, 2=about half the time, 3=most of the time, 4=all the time. Severity: 0=symptom not present, 1=mild, 2=moderate, 3=severe, 4=very severe.

© 2025 Berry C et al. *JAMA Network Open.*

eTable 27. Post-Exertional Malaise at 3-Months, Part 2 (DePaul Symptom Questionnaire, Short Form) Additional Questions

	All	Standard Care	Intervention
N Randomised			
N	195	100	95
If you were to become exhausted after actively participating in extracurricular activities, sports, or outings with friends, would you recover within an hour or two after the activity ended?			
N ($N_{MISSING}$)	99 (96)	51 (49)	48 (47)
N (%) Yes	42 (42.4%)	26 (51.0%)	16 (33.3%)
N (%) No	57 (57.6%)	25 (49.0%)	32 (66.7%)
Do you experience a worsening of your fatigue/energy related illness after engaging in minimal physical effort?			
N ($N_{MISSING}$)	99 (96)	51 (49)	48 (47)
N (%) Yes	66 (66.7%)	32 (62.7%)	34 (70.8%)
N (%) No	33 (33.3%)	19 (37.3%)	14 (29.2%)
Do you experience a worsening of your fatigue/energy related illness after engaging in minimal mental effort?			
N ($N_{MISSING}$)	99 (96)	51 (49)	48 (47)
N (%) Yes	54 (54.5%)	25 (49.0%)	29 (60.4%)
N (%) No	45 (45.5%)	26 (51.0%)	19 (39.6%)
If you feel worse after activities, how long does this last?			
N ($N_{MISSING}$)	98 (97)	50 (50)	48 (47)
N (%) ≤1h	20 (20.4%)	11 (22.0%)	9 (18.8%)
N (%) 2-3h	21 (21.4%)	16 (32.0%)	5 (10.4%)
N (%) 4-10h	13 (13.3%)	5 (10.0%)	8 (16.7%)
N (%) 11-13h	2 (2.0%)	0 (0.0%)	2 (4.2%)
N (%) 14-23h	8 (8.2%)	2 (4.0%)	6 (12.5%)
N (%) ≥24h	34 (34.7%)	16 (32.0%)	18 (37.5%)
If you do not exercise, is it because exercise makes your symptoms worse?			

© 2025 Berry C et al. *JAMA Network Open.*

	All	Standard Care	Intervention
N ($N_{MISSING}$)	99 (96)	51 (49)	48 (47)
N (%) Yes	61 (61.6%)	31 (60.8%)	30 (62.5%)
N (%) No	38 (38.4%)	20 (39.2%)	18 (37.5%)

© 2025 Berry C et al. *JAMA Network Open.*

eTable 28. Post-Exertional Malaise at 3-Months, Part 3 (DePaul Symptom Questionnaire, Short Form) Composite Measures

	All	Standard Care	Intervention
N Randomised			
N	195	100	95
Indication of post-exertional malaise			
N ($N_{MISSING}$)	99 (96)	51 (49)	48 (47)
N (%) No	17 (17.2%)	9 (17.6%)	8 (16.7%)
N (%) Yes	82 (82.8%)	42 (82.4%)	40 (83.3%)
Indication of ME and/or CFS diagnosis			
N ($N_{MISSING}$)	99 (96)	51 (49)	48 (47)
N (%) Yes	38 (38.4%)	15 (29.4%)	23 (47.9%)
N (%) No	61 (61.6%)	36 (70.6%)	25 (52.1%)

© 2025 Berry C et al. *JAMA Network Open.*

eTable 29. 3-Month Follow-up Data: Secondary Outcomes: Accelerometry - Changes From Baseline

	All	Standard Care	Intervention
N Randomised			
N	195	100	95
Sleep Time (min/day)			
N ($N_{MISSING}$)	110 (85)	57 (43)	53 (42)
Mean (SD)<br>Median (IQR)<br>[Min, Max]	-2.4 (107.5)<br>-6.0 (-78.0, 73.5)<br>[-201.0, 306.0]	10.1 (109.6)<br>-3.0 (-49.0, 76.0)<br>[-201.0, 306.0]	-16.0 (104.6)<br>-12.0 (-91.0, 59.0)<br>[-194.0, 187.0]
Sleep Efficiency (%)			
N ($N_{MISSING}$)	110 (85)	57 (43)	53 (42)
Mean (SD)<br>Median (IQR)<br>[Min, Max]	-0.9 (7.2)<br>-0.7 (-4.7, 3.3)<br>[-22.3, 18.7]	0.3 (7.2)<br>0.4 (-4.7, 3.8)<br>[-20.8, 18.7]	-2.1 (7.0)<br>-1.4 (-4.3, 2.7)<br>[-22.3, 9.8]
Inactive Time (min/day)			
N ($N_{MISSING}$)	110 (85)	57 (43)	53 (42)
Mean (SD)<br>Median (IQR)<br>[Min, Max]	-10.7 (100.2)<br>-10.0 (-60.8, 35.5)<br>[-268.0, 321.0]	-16.0 (100.8)<br>-22.0 (-72.0, 36.0)<br>[-238.0, 321.0]	-4.9 (100.3)<br>-5.0 (-58.0, 34.0)<br>[-268.0, 227.0]
Light Activity Time (min/day)			
N ($N_{MISSING}$)	110 (85)	57 (43)	53 (42)
Mean (SD)<br>Median (IQR)<br>[Min, Max]	3.4 (44.6)<br>-2.0 (-22.8, 22.2)<br>[-94.0, 194.0]	4.5 (50.5)<br>-1.0 (-26.0, 20.0)<br>[-94.0, 194.0]	2.3 (37.8)<br>-2.0 (-14.0, 23.0)<br>[-63.0, 123.0]
Moderate Activity Time (min/day)			

© 2025 Berry C et al. *JAMA Network Open.*

	All	Standard Care	Intervention
N ($N_{MISSING}$)	110 (85)	57 (43)	53 (42)
Mean (SD)	-0.5 (26.0)	-2.4 (22.2)	1.5 (29.7)
Median (IQR)	0.0 (-16.8, 14.8)	0.0 (-18.0, 15.0)	1.0 (-12.0, 14.0)
[Min, Max]	[-97.0, 73.0]	[-61.0, 38.0]	[-97.0, 73.0]
Vigorous Activity Time (min/day)			
N ($N_{MISSING}$)	110 (85)	57 (43)	53 (42)
Mean (SD)	-0.1 (2.9)	-0.1 (2.0)	-0.1 (3.6)
Median (IQR)	0.0 (-1.0, 1.0)	0.0 (-1.0, 1.0)	0.0 (-1.0, 1.0)
[Min, Max]	[-10.0, 15.0]	[-9.0, 5.0]	[-10.0, 15.0]

© 2025 Berry C et al. *JAMA Network Open.*

eTable 30. Secondary Outcome Analysis: hospitalization for any Reason (as Recorded in SAEs)

N (%) show people with at least one event of each type.

	All	Standard Care	Intervention
Number of patients with any SAE requiring hospitalization (of those randomised)			
N	6	5	1
Number of SAEs requiring hospitalization			
N	10	9	1
Seriousness Criterion: Death			
N (%) Yes	0 (0.0%)	0 (0.0%)	0 (0.0%)
Seriousness Criterion: Life threatening			
N (%) Yes	0 (0.0%)	0 (0.0%)	0 (0.0%)
Seriousness Criterion: Disability			
N (%) Yes	0 (0.0%)	0 (0.0%)	0 (0.0%)
Seriousness Criterion: Congenital abnormality			
N (%) Yes	0 (0.0%)	0 (0.0%)	0 (0.0%)
Seriousness Criterion: Other important medical event			
N (%) Yes	0 (0.0%)	0 (0.0%)	0 (0.0%)
Duration of hospitalization (days)			
N ($N_{MISSING}$)	10 (0)	9 (0)	1 (0)
Mean (SD)<br>Median (IQR)<br>[Min, Max]	4.0 (1.8)<br>4.0 (2.2, 5.0)<br>[2.0, 7.0]	3.9 (1.8)<br>4.0 (2.0, 5.0)<br>[2.0, 7.0]	5.0 (-)<br>5.0 (5.0, 5.0)<br>[5.0, 5.0]

© 2025 Berry C et al. *JAMA Network Open.*

	All	Standard Care	Intervention
Severity			
N ($N_{MISSING}$)	10 (0)	9 (0)	1 (0)
N (%) Mild	2 (20.0%)	2 (22.2%)	0 (0.0%)
N (%) Moderate	7 (70.0%)	7 (77.8%)	0 (0.0%)
N (%) Severe	1 (10.0%)	0 (0.0%)	1 (100.0%)
Causality			
N ($N_{MISSING}$)	10 (0)	9 (0)	1 (0)
N (%) Yes	0 (0.0%)	0 (0.0%)	0 (0.0%)
N (%) No	10 (100.0%)	9 (100.0%)	1 (100.0%)
Outcome			
N ($N_{MISSING}$)	10 (0)	9 (0)	1 (0)
N (%) Recovered	7 (70.0%)	7 (77.8%)	0 (0.0%)
N (%) Recovered with sequelae	2 (20.0%)	1 (11.1%)	1 (100.0%)
N (%) Recovering	0 (0.0%)	0 (0.0%)	0 (0.0%)
N (%) Not recovered	0 (0.0%)	0 (0.0%)	0 (0.0%)
N (%) Unknown	1 (10.0%)	1 (11.1%)	0 (0.0%)
N (%) Fatal	0 (0.0%)	0 (0.0%)	0 (0.0%)
Duration (days)			
N ($N_{MISSING}$)	9 (0)	8 (0)	1 (0)
Mean (SD)	3.8 (1.7)	3.6 (1.8)	5.0 (-)
Median (IQR)	4.0 (2.0, 5.0)	3.5 (2.0, 4.2)	5.0 (5.0, 5.0)
[Min, Max]	[2.0, 7.0]	[2.0, 7.0]	[5.0, 5.0]
Fisher test for any SAE requiring hospitalization by randomised group		p=0.119	

© 2025 Berry C et al. *JAMA Network Open.*

eTable 31. Visits Involving Physiotherapy and Rehabilitation in the Community

	All	Standard Care	Intervention
**N with follow-up data**			
N	195	100	95
**N with any physiotherapy contact in past 3 months**			
N (%) Yes	27 (13.8%)	18 (18.0%)	9 (9.5%)
**Number of physiotherapy contacts in past 3 months**			
N ($N_{MISSING}$)	27 (0)	18 (0)	9 (0)
Mean (SD)<br>Median (IQR)<br>[Min, Max]	2.8 (2.9)<br>2.0 (1.0, 3.0)<br>[1.0, 15.0]	2.4 (1.6)<br>2.0 (1.0, 3.0)<br>[1.0, 6.0]	3.4 (4.5)<br>2.0 (1.0, 3.0)<br>[1.0, 15.0]
**Total duration of physiotherapy contact (minutes)**			
N ($N_{MISSING}$)	26 (1)	17 (1)	9 (0)
Mean (SD)<br>Median (IQR)<br>[Min, Max]	33.5 (17.4)<br>30.0 (22.5, 43.8)<br>[10.0, 90.0]	37.4 (19.5)<br>30.0 (30.0, 45.0)<br>[10.0, 90.0]	26.1 (9.6)<br>30.0 (20.0, 30.0)<br>[15.0, 45.0]
**N with any physiotherapy home visits**			
N	3	2	1
**Number of physiotherapy home visits**			
N ($N_{MISSING}$)	3 (0)	2 (0)	1 (0)
Mean (SD)<br>Median (IQR)<br>[Min, Max]	2.3 (0.6)<br>2.0 (2.0, 2.5)<br>[2.0, 3.0]	2.0 (0.0)<br>2.0 (2.0, 2.0)<br>[2.0, 2.0]	3.0 (-)<br>3.0 (3.0, 3.0)<br>[3.0, 3.0]
**N with any physiotherapy clinic visits**			
N	24	16	8

© 2025 Berry C et al. *JAMA Network Open.*

	All	Standard Care	Intervention
Number of physiotherapy clinic visits			
N ($N_{MISSING}$)	24 (0)	16 (0)	8 (0)
Mean (SD)<br>Median (IQR)<br>[Min, Max]	2.8 (3.0)<br>2.0 (1.0, 3.2)<br>[1.0, 15.0]	2.5 (1.7)<br>2.0 (1.0, 3.2)<br>[1.0, 6.0]	3.5 (4.8)<br>1.5 (1.0, 2.8)<br>[1.0, 15.0]
N with follow-up data			
N	195	100	95
N with any rehabilitation service contact in past 3 months			
N (%) Yes	5 (2.6%)	1 (1.0%)	4 (4.2%)
Number of rehabilitation service contacts in past 3 months			
N ($N_{MISSING}$)	5 (0)	1 (0)	4 (0)
Mean (SD)<br>Median (IQR)<br>[Min, Max]	7.4 (1.7)<br>7.0 (6.0, 8.0)<br>[6.0, 10.0]	8.0 (-)<br>8.0 (8.0, 8.0)<br>[8.0, 8.0]	7.2 (1.9)<br>6.5 (6.0, 7.8)<br>[6.0, 10.0]
Total duration of rehabilitation service contact (minutes)			
N ($N_{MISSING}$)	5 (0)	1 (0)	4 (0)
Mean (SD)<br>Median (IQR)<br>[Min, Max]	40.0 (29.2)<br>30.0 (20.0, 40.0)<br>[20.0, 90.0]	30.0 (-)<br>30.0 (30.0, 30.0)<br>[30.0, 30.0]	42.5 (33.0)<br>30.0 (20.0, 52.5)<br>[20.0, 90.0]
Number of rehabilitation service home visits			
N ($N_{MISSING}$)	5 (0)	1 (0)	4 (0)
Mean (SD)<br>Median (IQR)<br>[Min, Max]	0.0 (0.0)<br>0.0 (0.0, 0.0)<br>[0.0, 0.0]	0.0 (-)<br>0.0 (0.0, 0.0)<br>[0.0, 0.0]	0.0 (0.0)<br>0.0 (0.0, 0.0)<br>[0.0, 0.0]
Number of rehabilitation service clinic visits			

© 2025 Berry C et al. *JAMA Network Open.*

	All	Standard Care	Intervention
N ($N_{MISSING}$)	5 (0)	1 (0)	4 (0)
Mean (SD)<br>Median (IQR)<br>[Min, Max]	7.4 (1.7)<br>7.0 (6.0, 8.0)<br>[6.0, 10.0]	8.0 (-)<br>8.0 (8.0, 8.0)<br>[8.0, 8.0]	7.2 (1.9)<br>6.5 (6.0, 7.8)<br>[6.0, 10.0]

© 2025 Berry C et al. *JAMA Network Open.*

eFigure 1. The Distribution in the Distances (m) Achieved During the Incremental Shuttle Walk Test at Baseline, 3-Months Post-Randomization, and the Change at 3-Months From Baseline

(A). Histogram of the primary outcome – distribution of the incremental shuttle walk test distance (m) by randomised group

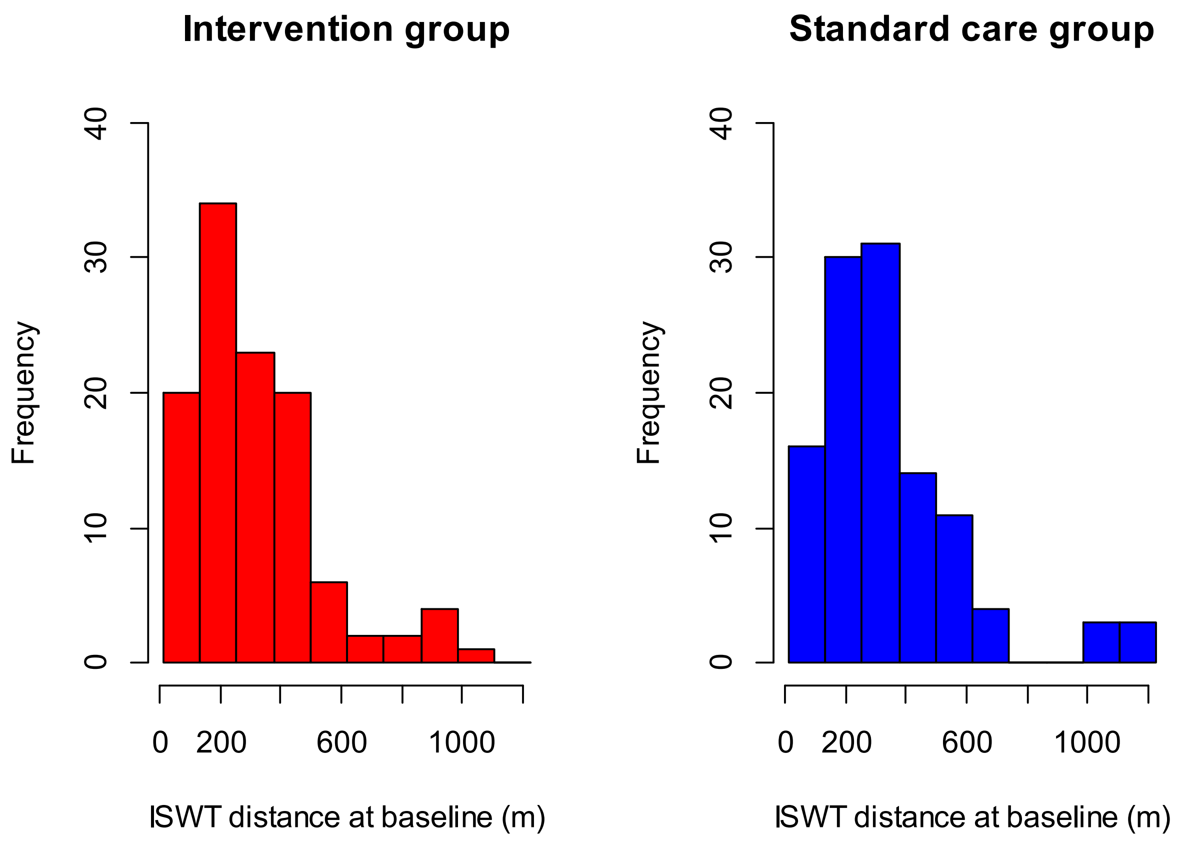

© 2025 Berry C et al. *JAMA Network Open.*

(B). Histogram of the primary outcome – distribution of the incremental shuttle walk test distance (m) by randomised group at 3-months post-randomization.

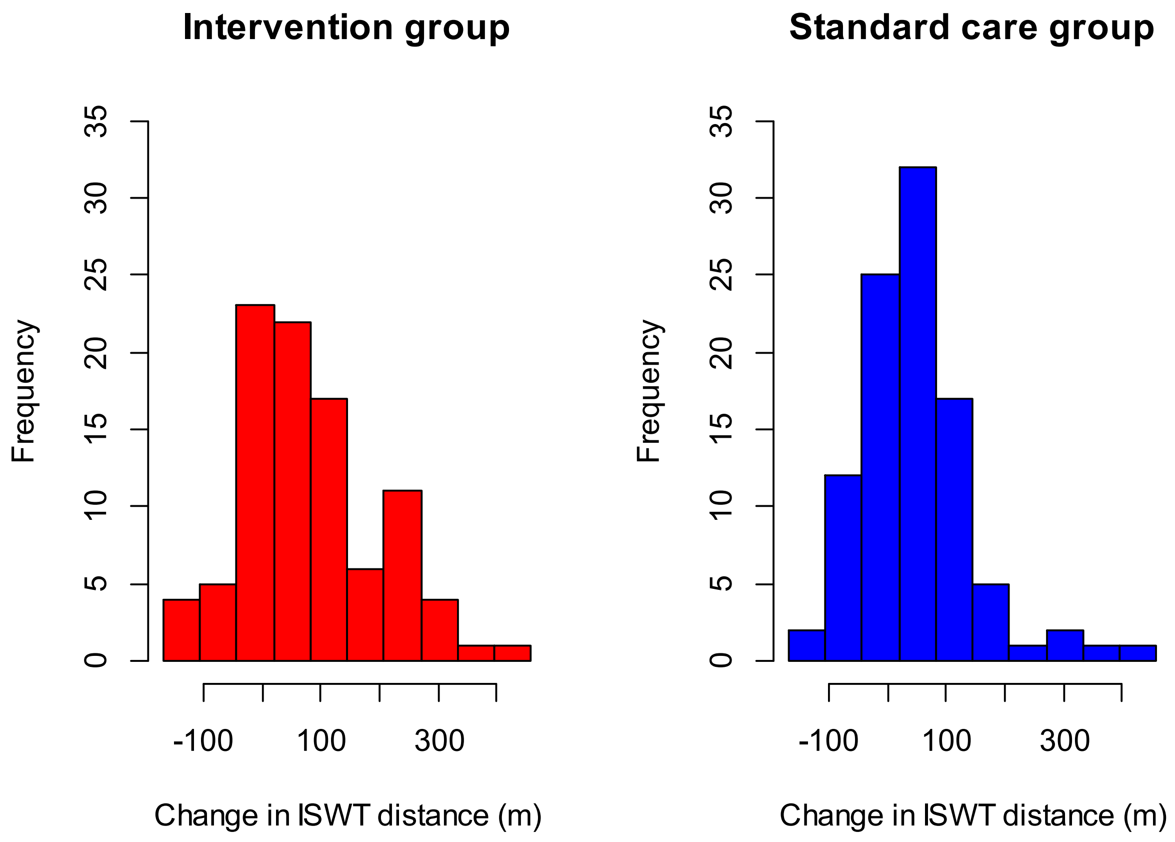

© 2025 Berry C et al. *JAMA Network Open.*

(C). Histogram of the primary outcome – distribution of the change in the incremental shuttle walk test distance (m) by randomised group at 3-months from baseline.

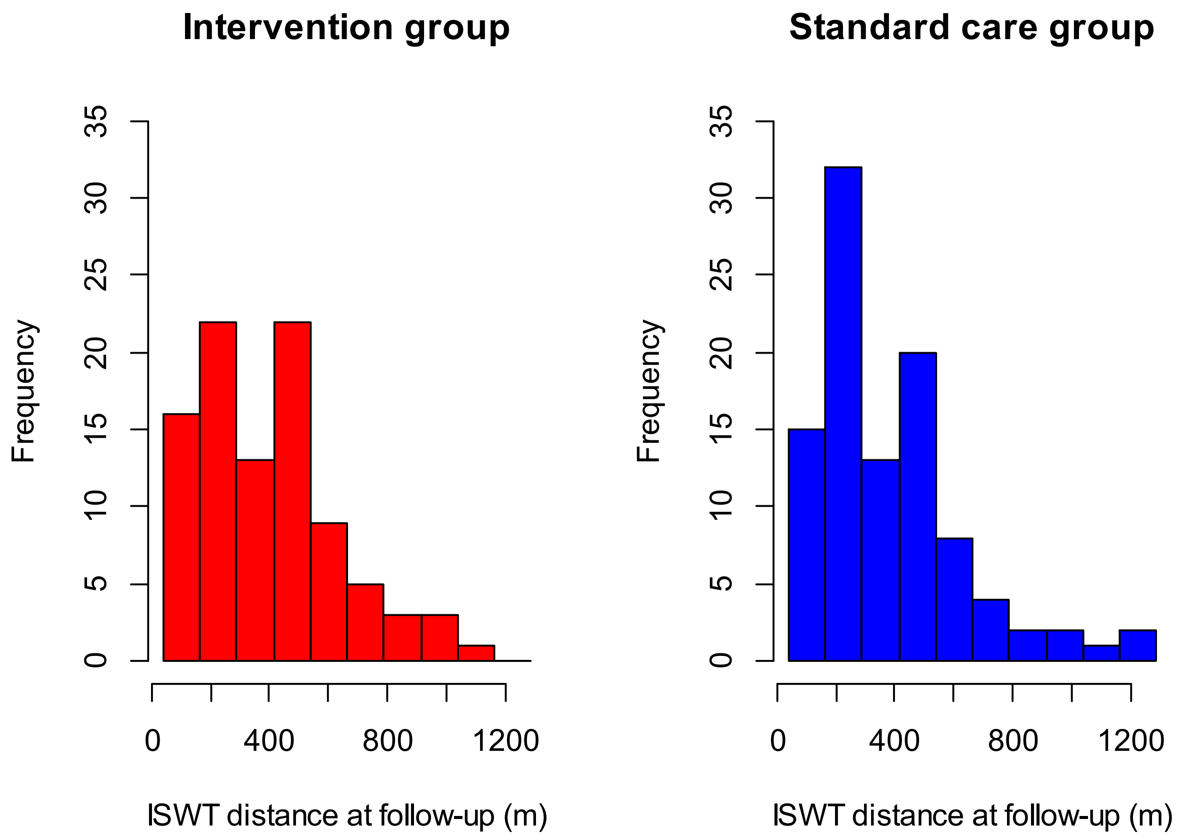

© 2025 Berry C et al. *JAMA Network Open.*

eFigure 2. Cohen's D Forest Plot Depicting Standardized Intervention Effect Estimates and 95% Confidence Intervals for Primary and Secondary Outcome Measures

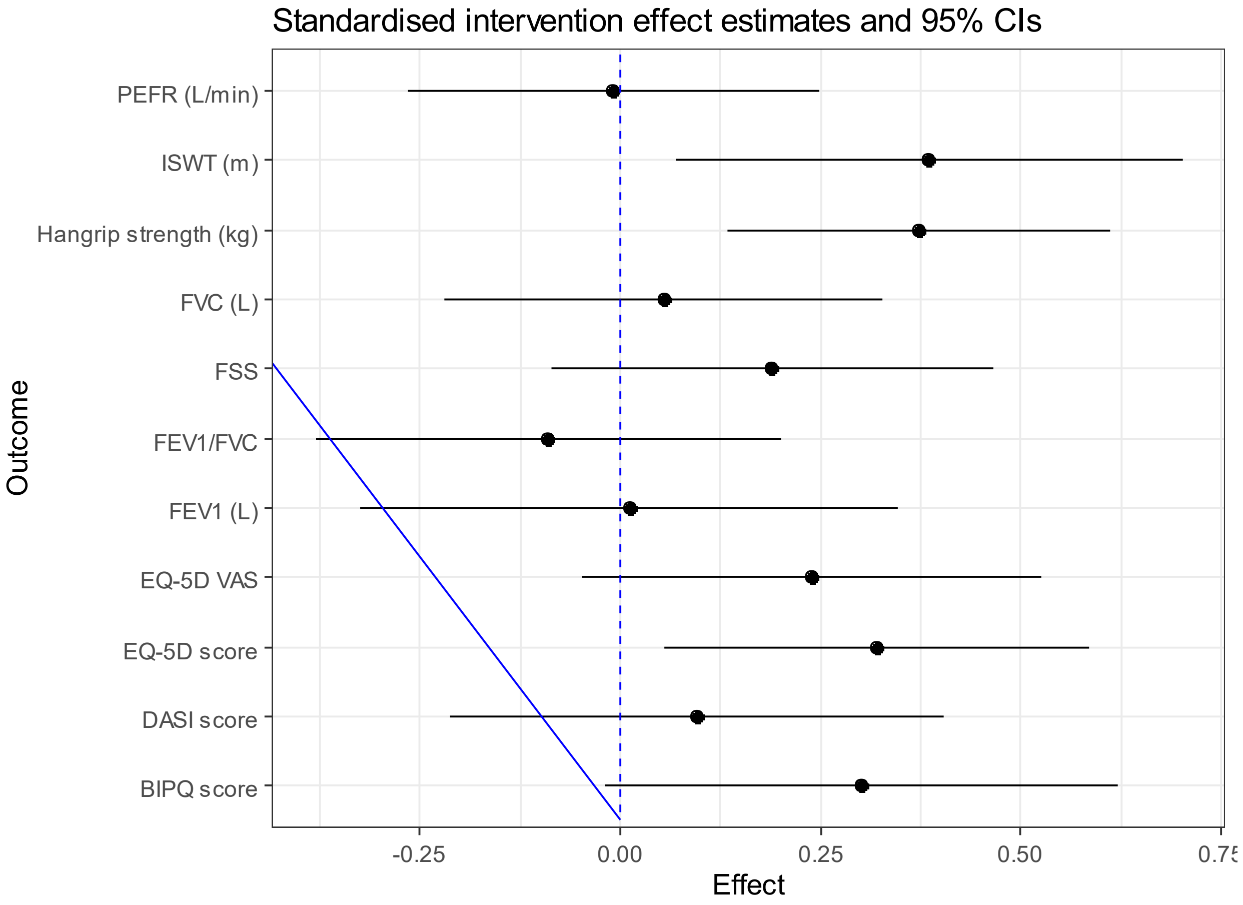

The effect estimates on each outcome measure and corresponding confidence intervals have been standardized via dividing by the standard deviation of the change in outcome at 3-months compared to baseline in the Standard Care population.

ISWT - Incremental shuttle walk test; PEFR – peak expiratory flow rat; FVC – forced vital capacity; FSS – fatigue severity score; EQ-5D VAS - Euroqol-5 dimension – 5-level visual analog score; DASI - Duke Activity Status Index; International Physical Activity Questionnaire (IPAQ-SF); Brief IPQ - Brief Illness perception questionnaire.

© 2025 Berry C et al. *JAMA Network Open.*

eFigure 3. Primary Outcome - Incremental Shuttle Walk Test Distance Intervention Effect estimates From Complier Average Causal Effects Analyses

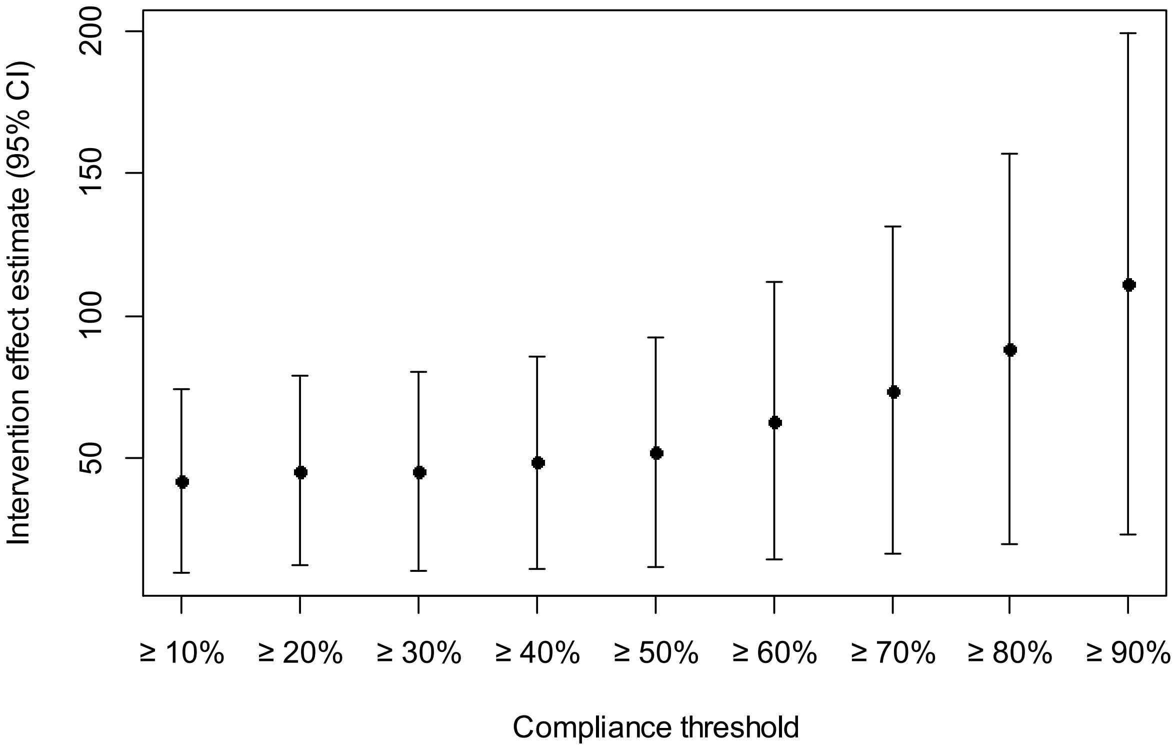

© 2025 Berry C et al. *JAMA Network Open.*

eFigure 4. EuroQol-5D-5L Life Utility Score (EQ-5D-5L)

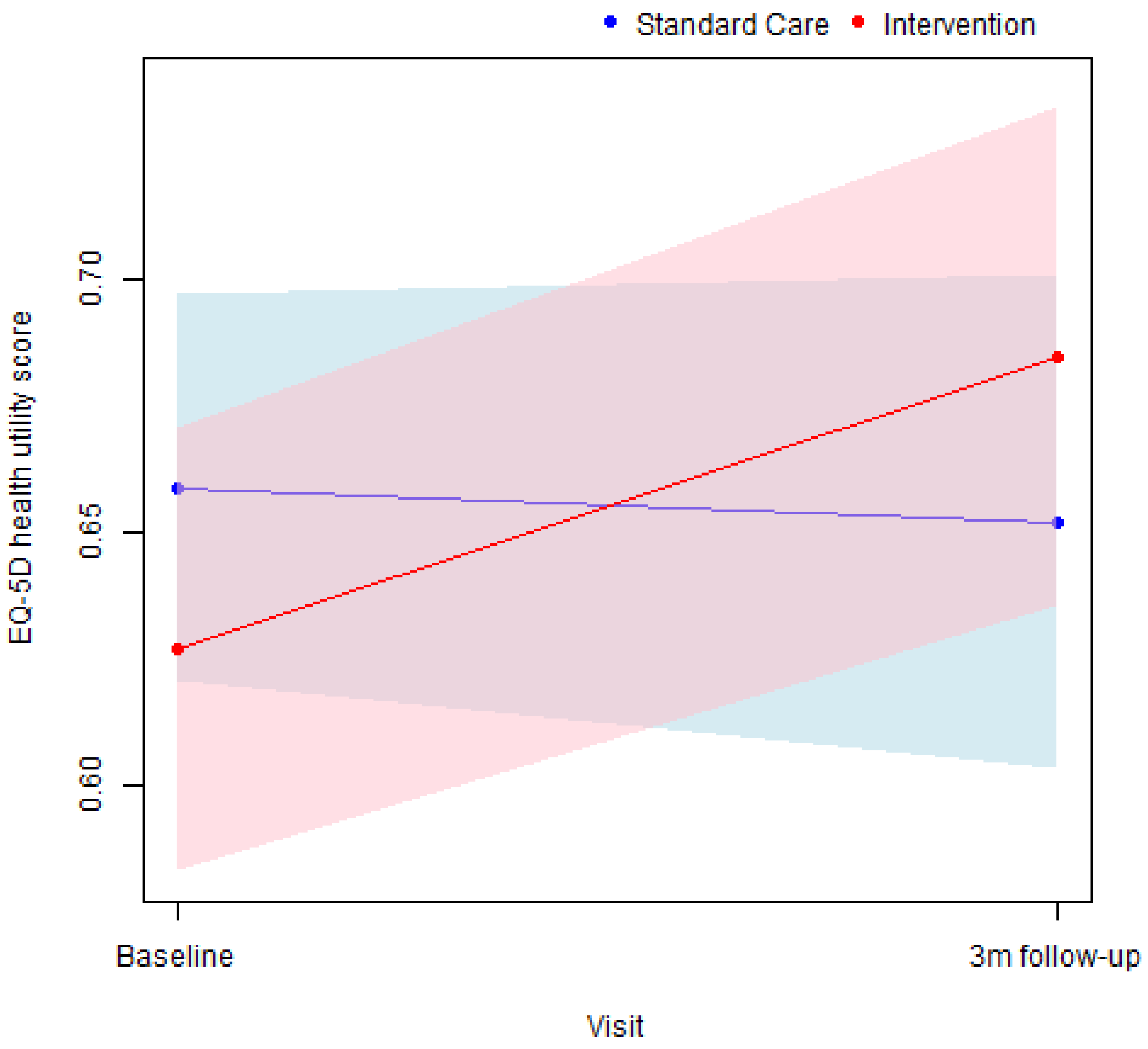

© 2025 Berry C et al. *JAMA Network Open.*

Health-related quality of life reflected by the patient-reported EuroQuol-5D-5L life utility score (mean ± 95% confidence interval) at baseline and 3-months follow-up in the intervention (red) and control (blue) groups, respectively. Intervention group = resistance exercise and standard care; Standard care group = no resistance exercise. Change from baseline in health-related quality of life at 3-months reflected by the patient-reported EuroQuol-5D-5L life utility score (EQ-5D-5L) (0.06 (0.01, 0.11); p=0.018).

© 2025 Berry C et al. *JAMA Network Open.*

eFigure 5. Patient Health Questionnaire-4 (PHQ-4) Category

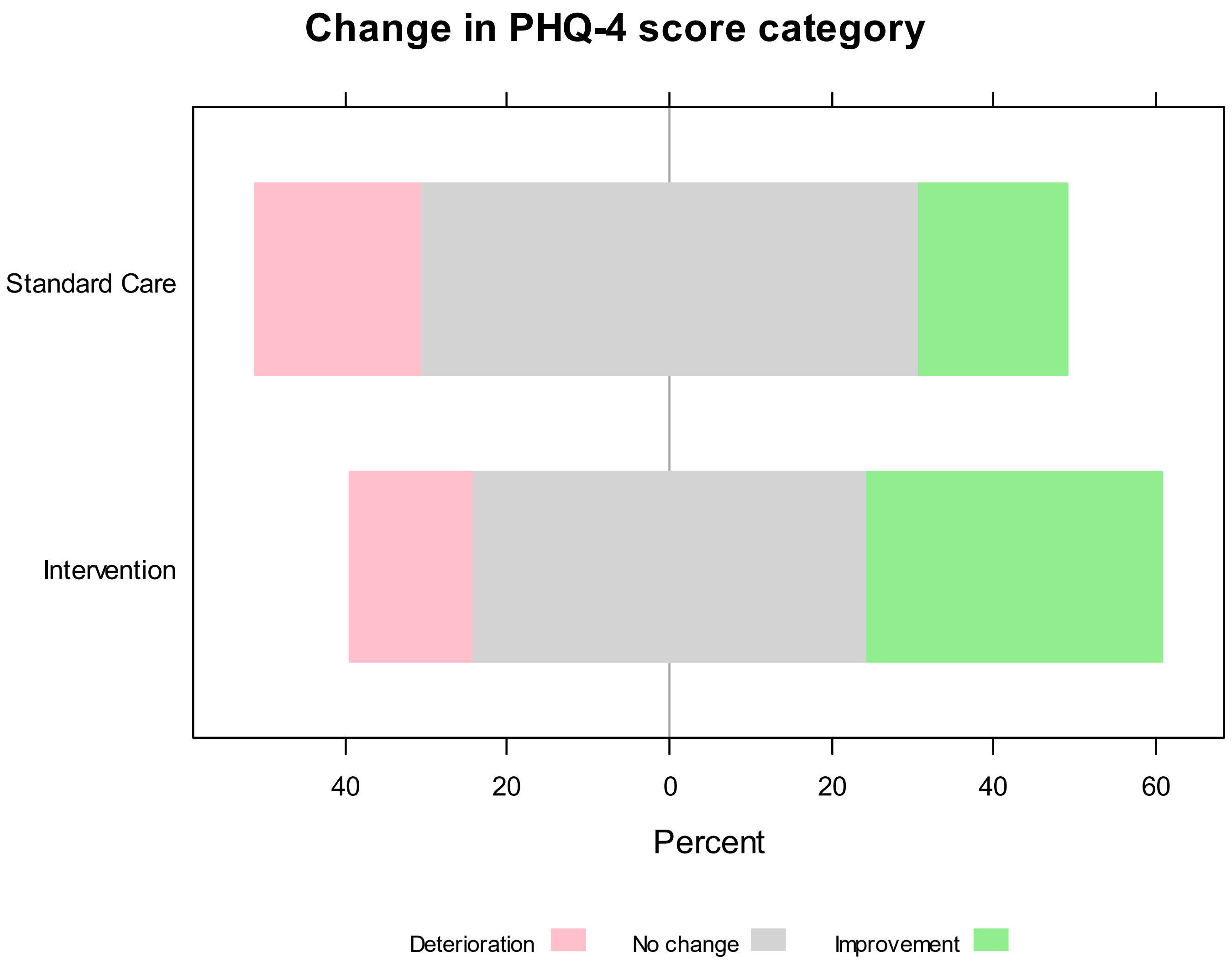

© 2025 Berry C et al. *JAMA Network Open.*

Change from baseline in PHQ-4 category at 3-months by randomised group. Intervention group = resistance exercise and standard care; Standard care group = no resistance exercise. Change from baseline in PHQ-4 category at 3-months by randomised group (0.5 (0.2, 0.8); p=0.013).

© 2025 Berry C et al. *JAMA Network Open.*

eFigure 6. Handgrip Strength. Figure S6. Handgrip strength (kg) (mean ± 95% confidence interval) at baseline and 3-months follow-up in the intervention (red) and control (blue) groups, respectively. Intervention group = resistance exercise and standard care; Standard care group = no resistance exercise. Change from baseline in handgrip strength (kg) at 3-months by randomised group (handgrip strength (2.58 (0.92, 4.24) kg; p=0.002).

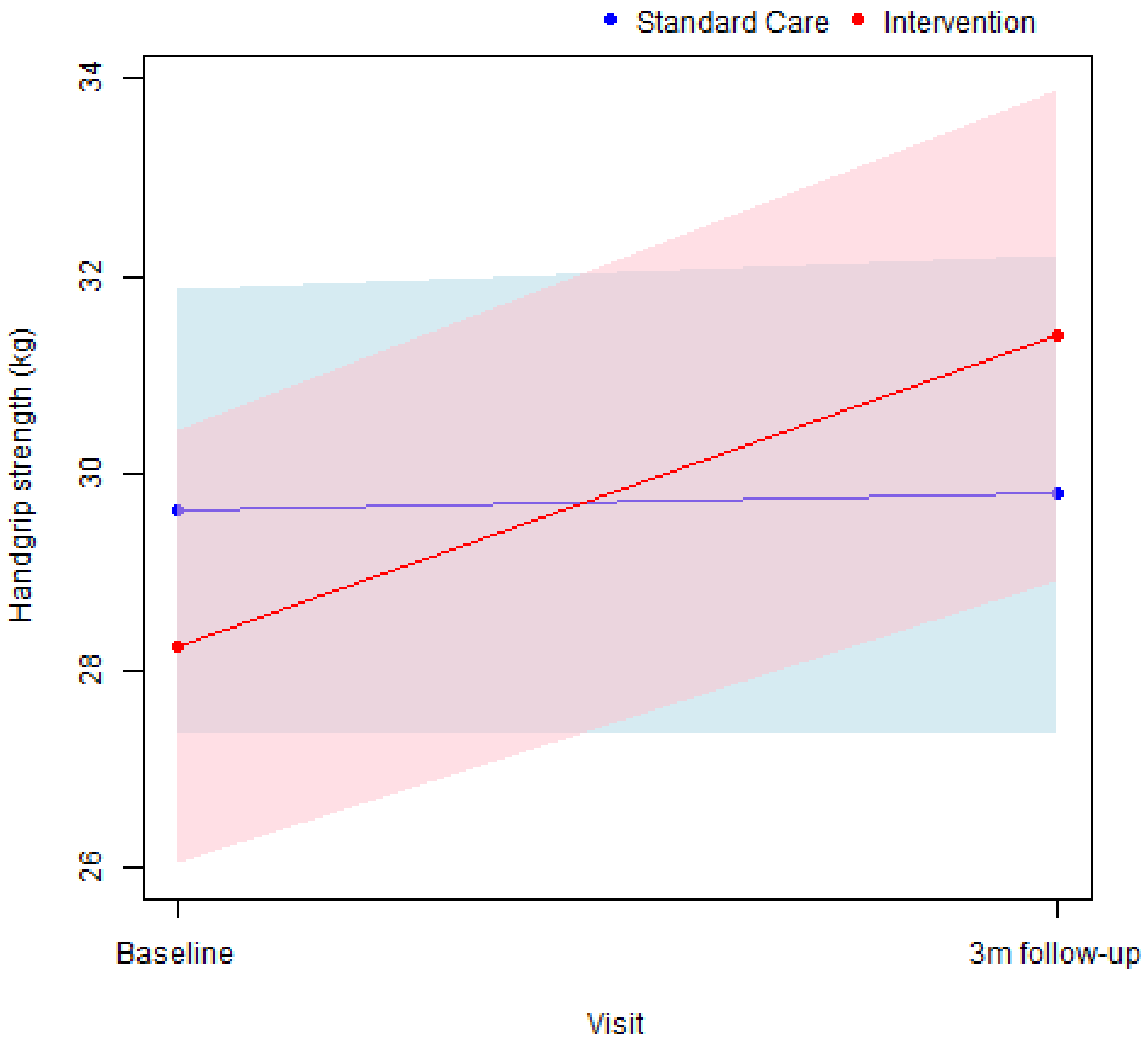

© 2025 Berry C et al. *JAMA Network Open.*
