# Supplementary material for: Resistance Exercise Therapy After COVID-19 Infection: A Randomized Clinical Trial
Source: JAMA Netw Open. 2025 Nov 10;8(11):e2534304. doi: 10.1001/jamanetworkopen.2025.34304 (PMC12603858; doi:10.1001/jamanetworkopen.2025.34304)
Supplement: Supplement 3. — Data Sharing Statement [file jamanetwopen-e2534304-s003.pdf]

# Data Sharing Statement

Berry. Resistance Exercise Therapy After COVID-19 Infection. *JAMA Netw Open*. Published September 29, 2025. doi:10.1001/jamanetworkopen.2025.34304

**Data**
**Additional Information:** NCT04900961
**Data available:** Yes
**Data types:** Data dictionary
**How to access data:** Variable list, statistical code, +/- and source data access pending sponsor approval
**When available:** With publication

**Supporting Documents**
**Document types:** Statistical/analytic code
**How to access documents:** Analysis feasible by access to a portal provided by the CTU pending sponsor approval.
**When available:** With publication

**Additional Information**
**Who can access the data:** researchers whose proposed use of the data has been approved
**Types of analyses:** Statistical code, web-based access
**Mechanisms of data availability:** web-based access
